# The characterization and antibiotic resistance profiles of clinical *Escherichia coli* O25b-B2-ST131 isolates in Kuwait

**DOI:** 10.1186/s12866-014-0214-6

**Published:** 2014-08-28

**Authors:** Ali A Dashti, Leila Vali, Sherief El-Shazly, Mehrez M Jadaon

**Affiliations:** Department of Medical Laboratory Sciences, Faculty of Allied Health Sciences, Kuwait University, P.O. Box 31470, Sulaibekhat, 90805 Sulaibekhat, Kuwait

**Keywords:** *Escherichia coli* ST131, Pulsed-field gel electrophoresis, Extended spectrum beta-lactamases, *qnr*B

## Abstract

**Background:**

*Escherichia coli* O25b-B2-ST131 are considered virulent extra-intestinal pathogens causing serious clinical complications such as urinary tract infection and bacteraemia. Our main objectives in this study were to characterise the multi-drug resistant (MDR) isolates of this lineage in Kuwait, and to demonstrate whether reduced susceptibility is spread clonally.

**Results:**

A subset of 83 (10%) non-duplicate and non-selective *E. coli* O25b-B2-ST131 out of 832 MDR *E. coli* was identified and collected. Minimum inhibitory concentrations of the isolates were determined and pulsed-field gel electrophoresis was used for typing.

The majority (95.2%) of the 83 *E. coli* O25b-B2-ST131 harboured at least one *bla* gene with *bla*_CTX-M-15_ being the most prevalent. *bla*_CTX-M-2_ was present in one isolate. Also one isolate harboured *bla*_CTX-M-56_, *qnr*B1 and *bla*_CMY-2_ genes and carried IncF1 plasmids of about 97 kb and160 kb. *qnr*B and *qnr*S were found in 8 other *bla*_CTX-M-15_ containing isolates. The *bla*_NDM_, *bla*_IMP_, *bla*_VIM_ and *qnr*A were not detected, however, the *bla*_OXA-48_ was present in two (2.4%).

**Conclusions:**

The majority of isolates harbouring *qnr* genes demonstrated relatedness (≥85%) by PFGE. However, the diversity in PFGE profiles for the other MDR isolates reflected the changes in population genetics of *E. coli* O25b-B2-ST131. We identified for the first time the appearance of *bla*_CTX-M-2_ in the Middle East and *bla*_CTX-M-56_ outside the Latin American countries. The isolate harbouring *bla*_CTX-M-56_ also contained *qnr*B1 and *bla*_CMY-2_ genes and carried IncF1 plasmids. The appearance of a highly virulent *E. coli* O25b-ST131 that is resistant to penicillins, most cephalosproins, β-lactamase inhibitors as well as fluoroquinolones is a cause for concern.

**Electronic supplementary material:**

The online version of this article (doi:10.1186/s12866-014-0214-6) contains supplementary material, which is available to authorized users.

## Background

*Escherichia coli* belonging to the phylogenic group B2, serotype O25b:H4 and Multi-Locus Sequence Type (ST) 131 (*E. coli* O25b-B2-ST131), producing extended-spectrum β-lactamase (ESBL) is regarded as a major pandemic clone in community and hospitals causing serious clinical infections such as urinary tract infections and bacteraemia [1]. It has been shown that *E. coli* O25b-B2-ST131 exhibits a high virulence score compared to other lineages [2] and is capable of acquiring antibiotic resistance by different mechanisms [3–6]. The fact that *E. coli* O25b-B2-ST131 is able to exhibit antibiotic resistance means that the clinical environment within a hospital or community may actively select certain resistant strains [7] making the treatment of these infections increasingly difficult. Analysis by pulsed field gel electrophoresis (PFGE) has identified a high degree of genetic diversity among the *E. coli* O25b-B2-ST131 isolates; however, some types appear to be more common in certain regions than others [4].

An important cause of resistance in *E. coli* O25b-B2-ST131 is the production of β-lactamase enzymes. Some of the most prevalent of these are CTX-M-like enzymes as well as other types specifically TEM-1, TEM-24, SHV-12 and the plasmid-mediated AmpC CMY-2 [8–10]. Furthermore, CTX-M-15 producing strains often co-produce both OXA-1 as well as variants of an aminoglycoside-modifying enzyme that is responsible for reduced susceptibility both to the aminoglycosides and to some fluoroquinolones expressed by *aac(6’)-Ib-cr* genes [5,6]. Fluoroquinolone (FQ) resistance in Enterobacteriaceae is usually caused by mutations in the chromosomal genes coding for type II topoisomerases and changes in the expression of efflux pumps and porins. The rise of plasmid-mediated FQ resistance protein Qnr [11] has caused concern in antimicrobial treatment of Enterobacteriaceae whereby carbapenems are considered the best therapeutic option [12]. Nevertheless some Enterobactericeae can produce clinically important carbapenemases; the Ambler class B metallo-β-lactamases (NDM, IMP, VIM), the class A enzymes (KPC) and the class D oxacillinase enzymes (OXA-48). Until recently *E. coli* was less often affiliated with carbapenemases than *Klebsiella pneumoniae*, however, the recent emergence of *bla*_NDM_ gene (New Delhi metallo-β-lactamase) on plasmids in *E.coli* ST131strains has caused concern [13–15]. The NDM-like enzymes have been identified in different regions [16] including in clinical *K. pneumoniae* isolates from Kuwait [17] and Oman [18] in the Middle East.

The *bla*_OXA-48_ carbapenemase is mainly associated with the Tn*1999*-like transposon inserted into a single 62-kb IncL/M-type plasmid [19]. It has been detected in sporadic cases; *E. coli* ST1196 (also containing resistance genes: *bla*_CMY-2_, *bla*_SHV-12_ and *bla*_TEM-1_) and *E. coli* ST1431 (containing β-lactamase genes: *bla*_CTX-M-1_, *bla*_OXA-2_ and *bla*_TEM-1_) isolated from pet dogs [20] and *E. coli* (containing *bla*_CTX-M-15_ and *bla*_TEM-1_ genes) isolated from a Belgian patient with ventilator-associated pneumonia travelling back from Egypt [21].

To date reports from the Middle East has been focused on the sporadic and selective *E. coli* O25b-B2-ST131 cases [22] and a comprehensive study on the epidemiology of this lineage was lacking. Therefore we aimed to address this issue by systematically characterising the multi-drug resistant (MDR) isolates of *E. coli* O25b-B2-ST131 recovered from patients in order to use these findings as a source for future reference studies and surveillances.

## Methods

### Bacterial isolates

A survey of Extended Spectrum β-lactamase (ESBL)-producing Enterobacteriaceae was undertaken from January 2010 to December 2012. A subset of 832 MDR *E. coli* strains was collected from the microbiology laboratories of three major hospitals that serve the six governorates of Kuwait. All the three hospitals are tertiary health care providers with bed capacities of 300 for Ahmadi, 500 for Amiri and 600 for Yiaco-Adan. The average number of specimens processed each day varies from 500 to 700 which includes samples from out-patient and in-patient specialists units. 832 original isolates represent a subset of the isolates submitted to the clinical diagnostic laboratories of these centres.

Each patient was included only once in this study. A database was created based on the patient’s records that contained information; such as age, sex, hospital, location of care on each site, type of specimen and date of sampling. Specimens were processed by clinical staff members of the diagnostic laboratories using standard protocols. Cultures were performed on blood agar, MacConkey, Cystine lactose electrolyte deficient agar (CLED) and incubated aerobically and anaerobically as required. All isolates were identified at the species level based on colony morphology, biochemical analysis and by using Vitek2 (Vitek AMS; bioMérieux Vitek Systems Inc., Hazelwood, MO, USA). The isolates were stored in 10% skim milk and at -70°C.

To confirm the phylogenic grouping of *E. coli* O25b-B2-ST131, PCR amplification of the *pabB, trpA, chuA*, *yjaA* genes [23] and DNA fragment of TSPE4.C2 were carried out as described before [24]. The products were sequenced from both directions and analysed.

### Antimicrobial susceptibility testing

Antimicrobial susceptibility testing was determined by automated broth microdilution method (Vitek2) (Vitek AMS; BioMérieux Vitek Systems Inc., Durham, NC, USA) and the results were analysed according to the Clinical and Laboratory Standards Institute, CLSI (2012) guidelines [25]. The antibiotics tested in this study were: Amikacin, amoxicillin/clavulanic acid, amp/sulbactam, ampicillin, cefazolin, cefepime, cefotaxime, ceftazidime, ceftriaxone, cefuroxime, cefoxitin, cefpodoxime, cephalothin, ceftriazone, ciprofloxacin, gentamicin, imipenem, meropenem, levofloxacin, nitrofurantoin, norfloxacin, tetracycline, tobramycin, trimethoprim/sulfamethoxazole, piperacillin/tazobactam, pipercillin and tigecycline.

ESBL production was confirmed by vitek2 analyzer and disk diffusion. Minimum inhibitory concentration (MICs) of quinolones, fluoro-quinolones and β-lactams including carbapenems were determined using the E-test method (CLSI 2012) [25]. Isolates that showed resistance to at least three classes of antibiotics were considered as MDR. Isolates that were detected as resistant to cefoxitin were further investigated for the presence of an *amp*C β-lactamase by using multiplex PCR [8,26].

### Double-disc synergy method

ESBLs were detected as previously described [27] using the disc approximation and double-disc synergy methods and confirmed with cefotaxime and ceftazidime E-test ESBL strips (AB Biodisk, Biomerieux-diagnostics, Durham, NC, USA). For the disc approximation test, clavulanate diffusion from an amoxicillin–clavulanate (AMC30) disc was used to test for synergy with cefotaxime, ceftazidime, cefuroxime, cefepime and cefixime (Oxoid) as described previously [28]. For the double-disc synergy test, a ceftazidime disc (30 μg) was placed 30 mm away from a disc containing amoxicillin–clavulanate (60/10 μg). ESBL production was considered positive when an enhanced zone of inhibition was visible between the β-lactam and β-lactamase inhibitor-containing discs. For the E-test, ESBL strips containing ceftazidime and ceftazidime–clavulanate and strips containing cefotaxime and cefotaxime–clavulanate were used to determine the MIC ratio according to the manufacturer’s instructions (AB Biodisk, Biomerieux-diagnostics, Durham, NC, USA). Cultures were incubated aerobically at 37°C for 18–24 h. CTX-M-15 β-lactamase enzyme displays a catalytic activity toward ceftazidime.

### Modified Hodge test

The test inoculum (0.5 McFarland turbidity) was spread onto Mueller-Hinton agar plates and disks containing 30 μg ceftazidime (with and without 10 μg clavulanic acid) and 10 μg imipenem (with and without 750 μg EDTA) were placed on the surface of the media. The plates were incubated at 37°C overnight. *P. aeruginosa* NCTC 10662, *E. coli* NCTC 10418, and *S. aureus* NCTC 6571 were used as controls on every plate.

### Identification of resistance genes

The presence of resistant genes listed below was investigated by PCR assays. PCR was conducted in a GeneAmp 9700 (Perkin-Elmer, Waltham Massachusetts, USA) system using the conditions specified for each primer; corresponding to the source references. *bla*_TEM-1_& *bla*_SHV_, *bla*_CTX-M-like_ [9], *bla*_NDM_ [13], *bla*_OXA-1_ [3], *qnr*A and *qnr*S [29], *qnr*B [30], *aac(6’)-Ib Ib-cr* [31], *gyr*A & *par*C [32], *gyr*B & *par*E [33]; *intI*1 [34] & *intI*2 [35], *bla*_VIM_*, bla*_IMP_, *bla*_OXA-48_ [19], *amp*C [8], *IS* [36].

Amplified PCR products were purified with Qiagen purification kit (Qiagen Valencia, CA, USA) according to the manufacturer’s instructions and both strands were sequenced by automated AB13100 DNA sequencer (Applied Biosystems, Carlsbad, CA, USA) system. The BLAST program of the National Centre for Biotechnology Information (http://www.ncbi.nlm.nih.gov) was used to search and compare databases for similar nucleotide acid sequences.

### Pulsed-field gel electrophoresis

Pulsed-Field Gel Electrophoresis (PFGE) analysis was based on techniques described elsewhere [37]. After PFGE, the gels were stained with ethidium bromide and scanned. The analysis of the gels was performed using BioNumerics software version 7.1 (Applied Maths, Ghent, Belgium). This software facilitates the development of the algorithms necessary for the comparison of profiles of isolates based on the Dice coefficient and the hierarchic unweighted pair arithmetic average algorithm. Cluster analysis and phylogenetic trees were subsequently analysed with an optimization of 1.0% and a tolerance of 0.7%. Isolates were considered to belong to the same PFGE clone if their Dice similarity index was ≥85%.

### Plasmid analysis

Plasmids were extracted (Promega, Fitchburg, WI, USA) and characterized by PCR as described previously [38]. Plasmids from clinical isolates were detected using PFGE. A single block was incubated at 55°C for 1 hour with 1 unit of S1 nuclease (New England Biolabs, Ipswich, MA, USA) in Zinc Buffer (200 μl of 50 mM NaCl, 30 mM sodium acetate and 5 mM ZnSO_4_). Electrophoresis was performed at 6 V, 5-50s for 20 h [39].

### Resistance transfer assays

Mating experiments were performed with *E. coli* J62-2(Rif^R^) as the recipient strain. Cultures of the donor (KOC-10 harbouring *bla*_CTX-M-56,_*qnr*B1 and *bla*_CMY-2_ genes) and the recipient strain were grown in Luria-Berani (LB) broth (10^9^ cfu/ml) and mixed in the ratio of 1:4 and incubated for 5 hours at 37°C. Transconjugates (0.1 ml) were selected on LB agar plates containing rifampicin (150 mg/L) and cefotaxime (2 mg/L). The transconjugates were tested for antibiotic resistance followed by PCR of the resistance determinants.

## Result

### Bacterial isolates and the detection of O25b-ST131

All three hospitals participated during our study period; however there were inconsistencies in the level of strain contribution for each year. Therefore under-representation of *E. coli* multi-drug resistant isolates might exist. We tested a subset of 832 *E. coli* MDR (Table 1). Of which 83 (10%) were identified as the O25b-sequence type (ST) 131 clone of B2 phylogenic group. The principal source of isolation (81%) was urine; mainly from patients older than 60 years of age, these comprised 49% of all the urine specimens. The distribution of these 83 isolates and the source of isolation are presented in Table 2. (Also see Additional file 1).

**Table 1:** **The distribution of 832 isolates by hospital, year, and sample from which the isolates were originally recovered**

**Hospital**	**Total**	**Year**	**Urine**	**Blood**	**Wound**	**Gastric Fluid**	**Catheter tip**	**Ascetic Fluid**	**Eye**	**Pleural Fluid**	**Sputum**	**Tissue**	**Pus**
**Amiri (ADA)**	240	2010											
		2011	49	1	2			2			1	1	1
		2012	177	5	1								
**Ahmadi (KOC)**	236	2010	87	7	13	2	8				7		3
		2011	48	1							1		
		2012	51	2			1				2		3
**Yiaco-Adan (Y)**	356	2010											
		2011											
		2012	305	13	24	1	2		1	2			8

**Table 2:** **The distribution of 83 isolates by hospital, year, and sample from which the isolates were originally recovered**

**Hospital**	**Total no.**	**Year**	**Urine**	**Blood**	**Wound**	**Pus**	**Catheter tip**	**Ascetic Fluid**	**Eye**	**Pleural Fluid**	**Sputum**
Amiri (ADA)	9	2010									
		2011						1			
		2012	8								
Ahamdi (KOC)	57	2010	38	5	2	2	2				1
		2011	3								
		2012	3			1					
Yiaco-Adan (Y)	17	2010									
		2011									
		2012	13		2				1	1	

### PCR amplification and sequencing

Table 3 shows the distribution of the *bla* genes among the 83 isolates of *E. coli* O25b-ST131. Four (4.8%) did not contain any of the β-lactamase enzymes while the majority (95.2%) harboured at least one β-lactamase resistance gene. Two isolates harboured *bla*_CTX-M-2_ and *bla*_CTX-M-56_. *bla*_NDM_, *bla*_IMP_ and *bla*_VIM_ genes were not found. IS*Ecp*1 was detected upstream region of 25 (33%) of the *bla*_CTX-M-15_ positive isolates. *bla*_CMY-2_ was only detected in four isolates (4.8%). IS elements were detected in 2 *bla*_CMY-2_ positive isolates, 1 contained class 1 integrons and 1 class II integrons.

**Table 3:** **Molecular characterization of** ***bla*** **genes among** ***E. coli*** **O25b-B2-ST131in Kuwait**

**Profiles of the antibiotic resistance genes**	**No. of isolates (%)**
*bla*_TEM-1_	2 (2.4)
*bla*_SHV-12_	1 (1.2)
*bla*_CTX-M-2_	1 (1.2)
*bla*_CTX-M-15_	32 (38.6)
*bla*_CTX-M-56_	1 (1.2)
*bla*_TEM-1_, *bla*_SHV-12_	1 (1.2)
*bla*_CTX-M-15_, *bla*_SHV-12_	9 (10.8)
*bla*_CTX-M-15_, *bla*_TEM-1_	21 (25.3)
*bla*_CTX-M-15_, *bla*_TEM-1_, *bla*_SHV-12_	12 (14.5)

Class 1 integrons were identified in 30 (36.1%) isolates and only 5 (6%) contained class II integrons. None of the isolates contained both classes of integrons.

### Quinolone resistance determinants

All but two isolates were resistant or had intermediate resistance to ciprofloxacin (MIC > 2 mg/l). Two sensitive isolates did not contain *aac(6’)-Ib Ib-cr* (isolates Y-116 and Y-159). We did not detect *qnr*A gene in any of the isolates tested. Three isolates harboured *qnr*B1 and 4 harboured *qnr*S1. *qnr*B1 and *qnr*S1 coexisted in only 2 isolates (Table 4).

**Table 4:** **The profile of quinolone resistant** ***E. coli*** **O25b-B2-ST131isolates**

**Profiles of the antibiotic resistance genes**	**No. of Isolates**
*bla*_CTX-M-56_, *bla*_cmy-2_, *qnr*B1	**1**
*bla*_CTX-M-15_, *aac(6*’*)-Ib-cr*, *bla*_TEM-1,_ *qnr*B1	**1**
*bla*_CTX-M-15_, *aac(6*’*)-Ib-cr*, *bla*_OXA-1_, *bla*_TEM-1_, *qnr*B1, IS*Ecp*1	**1**
*bla*_CTX-M-15_, *aac(6*’*)-Ib-cr*, *bla*_OXA-1_,, *bla*_TEM-1_, *qnr*S1, IS*Ecp*1	**1**
*bla*_CTX-M-15,_ *aac(6*’*)-Ib-cr*, *bla*_OXA-1_,, *qnr*B1, *qnr*S1	**2**
*bla*_CTX-M-15,_ *aac(6*’*)-Ib-cr*, *bla*_OXA-1_,, *qnr*S1, IS*Ecp*1	**2**
*bla*_CTX-M-15_, *qnr*S1, *bla*_OXA-1_,, IS*Ecp*1	**1**
**Total**	**9**

**Figure 1:**
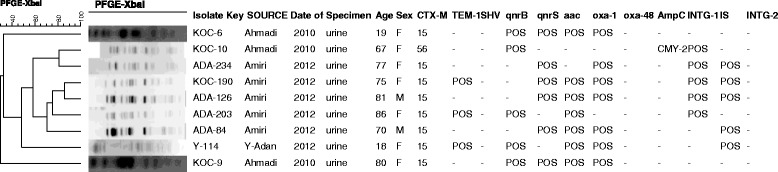
**PFGE profiles of** ***E. coli*** **O25b-B2-ST131isolates collected in this study harbouring** ***qnr*** **genes.** The degree of similarity is shown on the scale at the top left of the figure. **Isolate no. Specimen Age Gender.**

Fifty six (67.5%) isolates carried *aac(6’)-Ib Ib-cr*. Among the *aac(6’)-Ib Ib-cr* negative strains (27/83) 32.5%, 1 isolate carried *qnr*B1 and *bla*_CTX-M-56_ (KOC-10) and 1 isolate carried *qnr*S1 (ADA-234). The remaining 78% (7/9) *qnr* positive isolates also contained *aac(6’)-Ib Ib-cr* (Figure 1). None of the *qnr* positive isolates carried *bla*_SHV_.

No mutations were detected in the quinolone-resistance-determining regions of *gyr*A. However, there was a new mutation in isolate D-140 topoisomerase subunit IV at position 520 G to C that altered 174 Val (GTC) to Leu (CTC) possibly not leading to any additional chromosome encoded fluoroquinolone resistance. We also observed mutations in isolate Y-190 in topoisomerase subunit IV; the amino acid 560A → V and at position 840 V → A.

### PFGE

**Figure 2:**
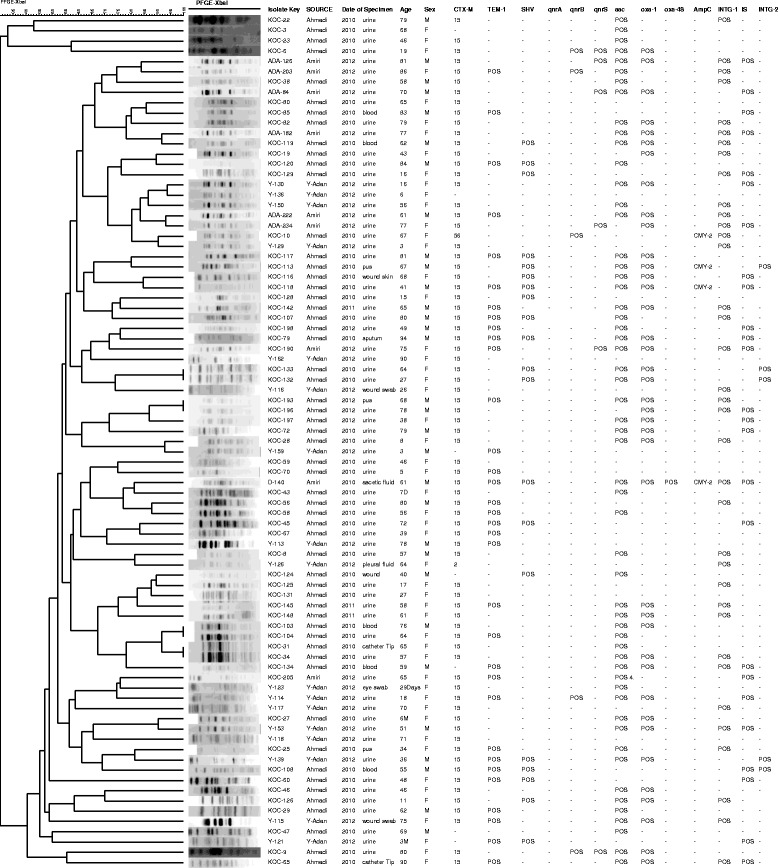
**Relationship between banding patterns after digestion with** ***Xba*** **I endonuclease enzyme showing the percentage similarity between group types and clusters for 83** ***E. coli*** **O25b-B2-ST131 isolates using DICE/UPGMA with an optimization of 1.0% and a tolerance of 0.5% generated by BioNumerics software (v.7.1).**

**Figure 3:**
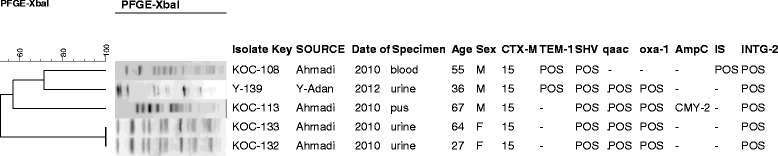
**PFGE profiles of** ***E. coli*** **O25b-B2-ST131isolates containing Class II integron.**

PFGE showed diverse genetic profiles (Figure 2). The isolates that harboured *qnr* genes; although resemble similar phenotypes; some displayed unrelated PFGE profiles suggesting that they were not epidemic cases (Figure 1). The genotyping results of the 5 isolates that contained class II integrons suggested that only two of these isolates have identical PF patterns and harboured similar antibiotic resistant profiles whereas the other three isolates were not closely related and contained different resistance genes including one isolate which contained the AmpC gene *bla*_CMY-2_. All 5 harboured *bla*_CTX-M-15_ (Figure 3).

### Antimicrobial susceptibility

We identified 3 (3.6%) of the *E. coli* O131 isolates did not contain β-lactam resistance genes which reflect the infection caused by cephalosporin-susceptible clones (KOC-3, KOC-47 and Y-136). These isolates were collected from two different hospitals, all from urine specimens and were not related by PFGE to each other but were closely related to other isolates that contained *bla*_CTX-M-15_ (Figure 2).

### Plasmid analysis

**Figure 4:**
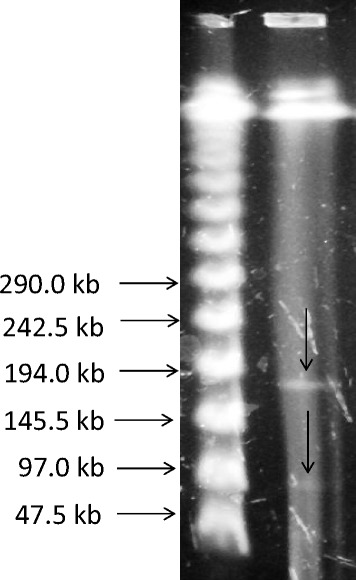
**Agarose gel showing S1 nuclease PFGE-based sizing of large plasmids from** ***E. coli*** **O25b-B2-ST131 harbouring** ***bla***_**CTX-M-56**_ **.**

IncFII plasmid that also contains β-lactamase gene *bla*_OXA-1_ that encodes for OXA-1 and the aminoglycoside/fluoroquinolone acetyl transferase aac(6’)-Ib-cr was present in 58 (70%) of isolates of which 33 (40%) contained both genes. The isolate (KOC10) harbouring *bla*_CTX-M-56_ gene also contained *qnr*B1 and *bla*_*CMY*-2_ genes and carried IncF1 plasmids of about 97 kb and 160 kb (Figure 4). Number of transconjugants in 1 ml for KOC10 was on average 40 to 6 × 10^2^ which comprised of 4 × 10^−8^ to 6 × 10^−7^ transconjugants per donor cell. PCR revealed that only one of the transconjugates contained *qnr*B1 and *bla*_CMY-2_ genes and one contained *qnr*B1 and *bla*_CTX-M-56_.

## Discussion

This manuscript reports the trend of *E. coli* O25b-ST131 isolated non-selectively in hospitals. During our two year study 10% of MDR *E. coli* isolated belonged to the *E. coli* O25b-ST131 clonal group indicating that the Middle East has joined the countries affected by this virulent pathogen posing a major public health concern. MDR *E. coli* O25b-ST131isolates were isolated from different age groups of patients (3-94 years old; with the average age of 54.4 years old).

The majority of isolates (38.6%) harboured only *bla*_CTX-M-15_ and 10.8% also contained *bla*_TEM_ and or *bla*_SHV_. Among ESBL producers; we detected the presence of *bla*_CTX-M-56_ for the first time in the Middle East and outside the South American continent [40]. The patient from which the isolate was recovered had an international travel history to an endemic region. Also we detected *bla*_CTX-M-2_, one of the dominant Asian β-lactamases [41] for the first time in the Middle East. *bla*_CTX-M-56_ gene is in the same context as *bla*_CTX-M-2_ by a single nucleotide mutation (G824A), resulting in a replacement of serine by asparagine at position 275 [42]. Previously no explanation was given as to what this change means, however we propose that based on other class A β-lactamases [43,44], as this modification takes place at the C terminal of the α-11 helix it is involved in the resistance to inactivation by β-lactamase inhibitors. The isolate harbouring *bla*_CTX-M-56_ also contained *qnr*B1 and *bla*_CMY-2_ genes and carried IncF1 plasmids of about 97 kb and160 kb. Production of plasmid AmpC such as *cmy* genes confers resistance to all penicillins, most cephalosporins and currently available β-lactamase inhibitors. Therefore the emergence of a clinical isolate that contains *bla*_CMY-2_ as well as *bla*_CTX-M-56_ poses a risk to combination β-lactam/ β-lactamase inhibitor therapy.

We also detected the presence of *qnr* genes in eight other *bla*_CTX-M-15_ harbouring isolates. Although Qnr enzyme by itself produces low-level resistance to quinolones, its presence facilitates the selection of higher-level resistance, thus contributing to the alarming increase in resistance to quinolones.

IS*Ecp*1-*bla*_CTX-M-15_ element was located in the upstream region of 33% of isolates harbouring *bla*_CTX-M-15_. Twenty seven per cent of which were associated with *bla*_SHV,_*bla*_TEM_ as well as *bla*_CTX-M-15_. IS*Ecp*1 plays a role in gene transfer or in providing a promoter for β-lactamase genes and supports their dissemination [45]. IncFII plasmid that also harboured *bla*_OXA-1_ and the aminoglycoside/fluoroquinolone acetyl transferase aac(6’)-Ib-cr gene (*aac(6’)-Ib Ib-cr*) was present in 59 (71%) of isolates of which 33 (40%) contained both genes. Two isolates containing *bla*_OXA-48_ contained IS*Ecp*1 and class 1 integrons. It has been reported [46] that a novel Tn1999 transposon inserted into a single 62-kb IncL/M-type plasmid is responsible for the dissemination of *bla*_OXA-48_ gene in *E. coli* strains.

The rate of carriage of MDR *E. coli* O25b-ST131 is estimated at 7% in healthy adults [47]; however the rate of *E. coli* O25b-ST131 susceptible to extended-spectrum cephalosporins has never been reported. We identified 3.6% of the *E. coli* O131 isolates did not contain any of the related resistance genes which reflect the infection caused by cephalosporin-susceptible clones.

## Conclusion

We did not find any association between resistance profiles and genotypes. However; we detected for the first time the appearance *bla*_CTX-M-2_ in the Middle East and *bla*_CTX-M-56_ outside Latin America. We also identified the spread of *qnr*B1 and *qnr*S1 in isolates harbouring *aac(6’)-Ib Ib-cr* and *bla*_CTX-M_. The isolate harbouring *bla*_CTX-M-56_ also contained *qnr*B1 and *bla*_CMY-2_ genes and carried IncF1 plasmids. In conclusion the appearance of a highly virulent *E. coli* O25b-ST131 that is resistant to penicillins, most cephalosproins, β-lactamase inhibitors as well as floroquinolones is a cause for concern and poses a risk to combination β-lactam/ β-lactamase inhibitor therapy.

## Additional file

Additional file 1: Table S1. Specimen types and Demographics of *E. coli* O25b-B2-ST131 isolates. Samples from pus, skin and wound have been illustrated under soft tissue.
