# Supplementary material for: The characterization and antibiotic resistance profiles of clinical Escherichia coli O25b-B2-ST131 isolates in Kuwait
Source: BMC Microbiol. 2014 Aug 28;14:214. doi: 10.1186/s12866-014-0214-6 (PMC4159528; doi:10.1186/s12866-014-0214-6)
Supplement: Additional file 1: Table S1. — Specimen types and Demographics of E. coli O25b-B2-ST131 isolates. Samples from pus, skin and wound have been illustrated under soft tissue. [file 12866_2014_214_MOESM1_ESM.zip › 12866_2014_214_MOESM1_ESM/12866_2014_214_add1.docx]

| Infection site of Specimens Male: Female | | | | | | | | |
| --- | --- | --- | --- | --- | --- | --- | --- | --- |
| Ascetic fluid | Pleural fluid | Soft tissue | Eye | Bacteraemia  Primary or secondary | Urine | Percentage % | No. Male:Female | Age(yr) |
|  |  |  | 0:1 |  | 0:3 | 4.8 | 0:4 | ≥1 |
|  |  |  |  |  | 1:4 | 6 | 1:4 | 2-10 |
|  |  |  |  |  | 0:7 | 8.4 | 0:7 | 11-20 |
|  |  | 2:1 |  |  | 1:4 | 9.6 | 3:5 | 21-40 |
|  |  |  |  | 2:0 | 5:9 | 19.3 | 7:9 | 41-60 |
| 1:0 | 1:0 | 3:4 |  | 3:0 | 14:17 | 51.8 | 22:21 | 61≤ |
