# Supplementary material for: The characterization and antibiotic resistance profiles of clinical Escherichia coli O25b-B2-ST131 isolates in Kuwait
Source: BMC Microbiol. 2014 Aug 28;14:214. doi: 10.1186/s12866-014-0214-6 (PMC4159528; doi:10.1186/s12866-014-0214-6)

```
  1   GGAAAGCATT TATGGAGCAC CCACCAACGA TATCGCGGTT ATCTTGGCCG GAAAACCACG CACCGCTGGT    70
 71   TCTGGTGACC TACTTTACCC AACCGGAGCA GAAGGCGGAA AGCCGTCGGG ATATTCTGGC TGCGGCGGCG   140
141   AAAATCGTAA CCCACGGTTT CTGATGCAAT AATCTAGAGC A                                  181
```

Applied Biosystems

S/N G:27 A:14 T:8 C:12
KB.bcp
KB 1.4.0 Cap:1

17_3130POP7_v3.1_2012-11-29
17
KB_3130_POP7_BDTv3.mob
Pts 2003 to 8532 Pk1 Loc:1972
Version 5.3 HiSQV Bases: 139

Inst Model/Name 3100/3130GeneticAnalyzer-19348-006
Nov 29,2012 12:43PM, AST
Nov 29,2012 12:54PM, AST
Spacing:9.78 Pts/Panel1500
Plate Name: 29112012

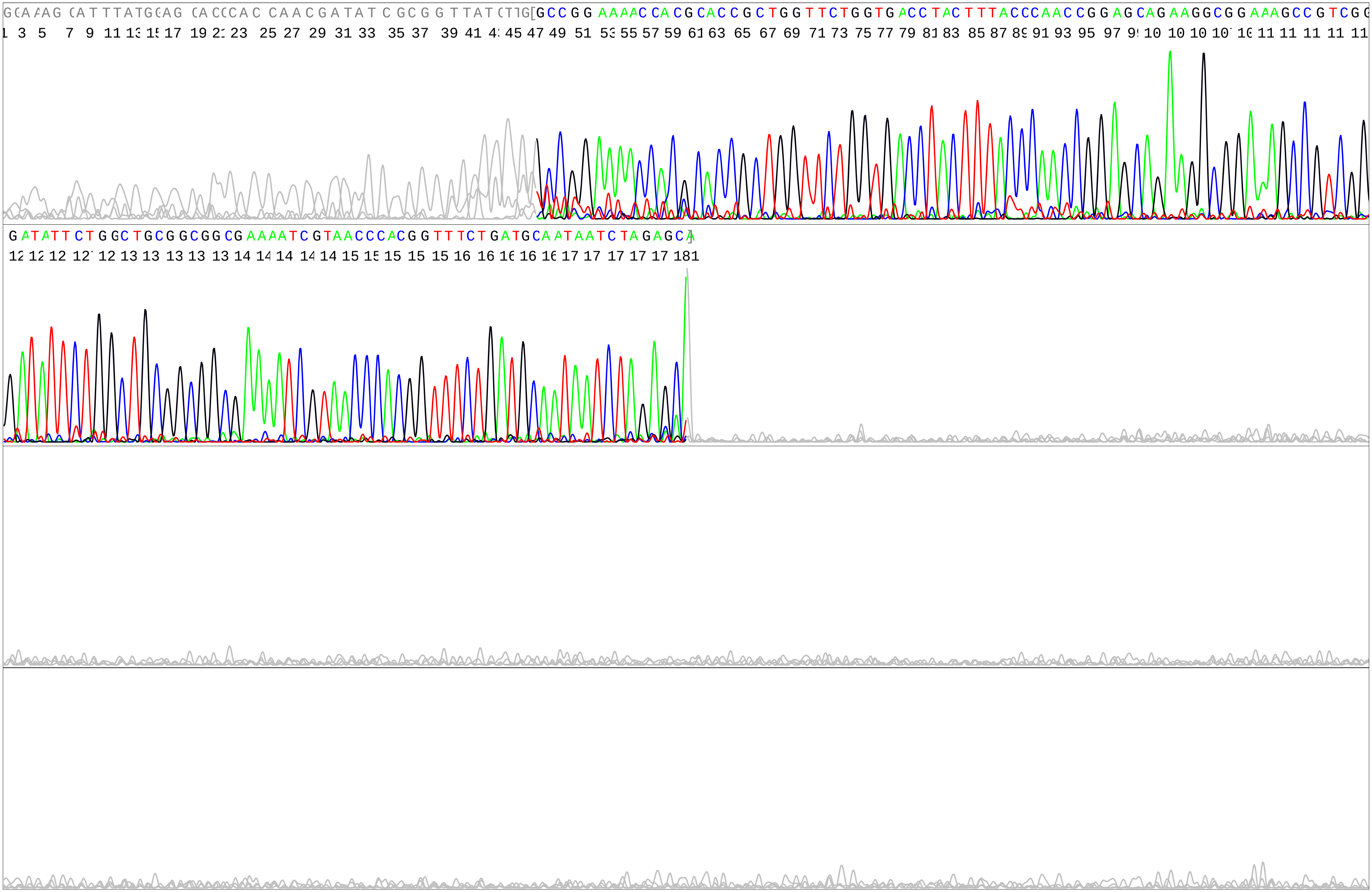

Printed on: Mon Dec 03,2012 10:22AM, AST

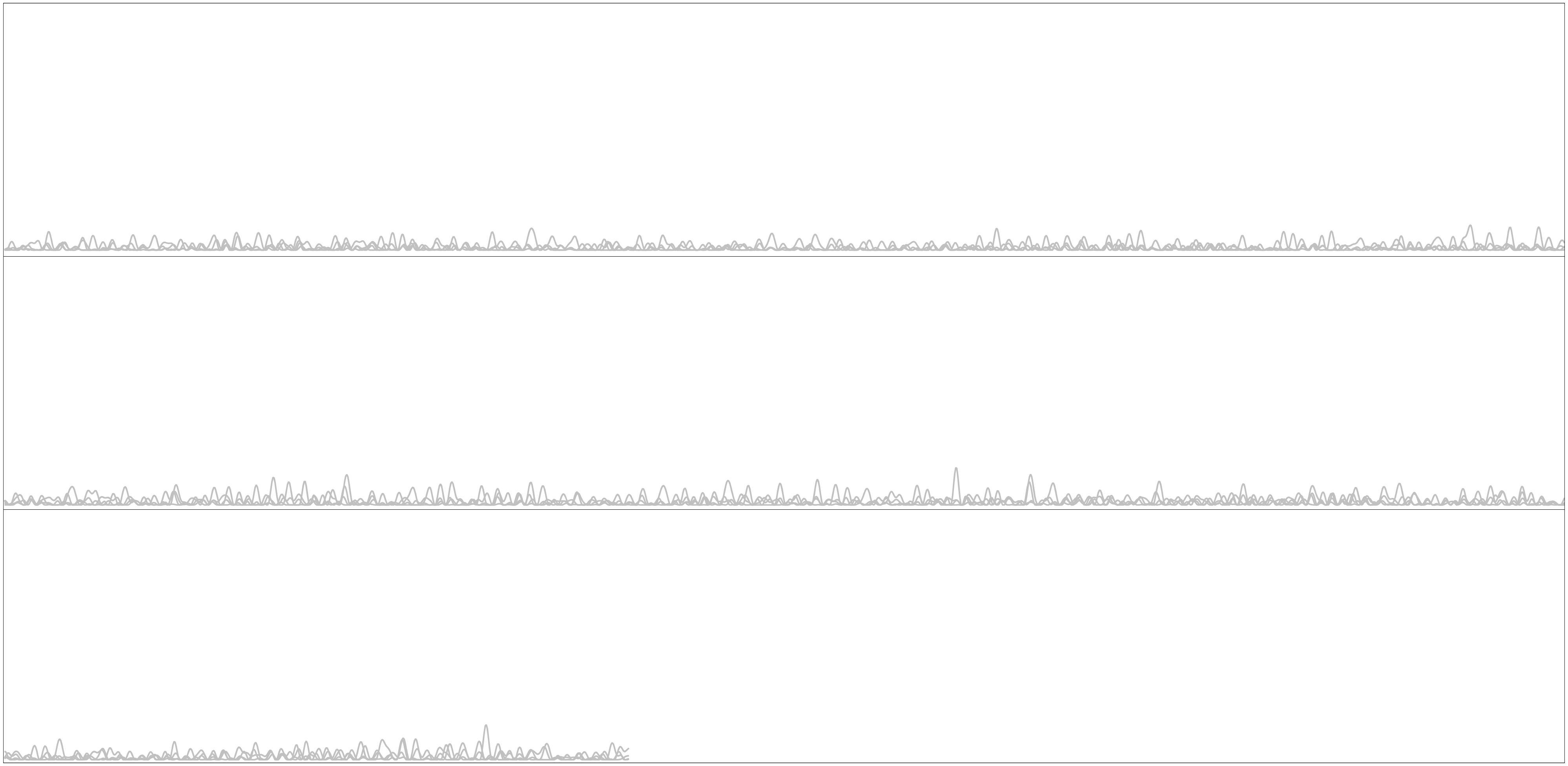

Supplement: Additional file 1: Table S1. — Specimen types and Demographics of E. coli O25b-B2-ST131 isolates. Samples from pus, skin and wound have been illustrated under soft tissue. [file 12866_2014_214_MOESM1_ESM.zip › 12866_2014_214_MOESM1_ESM/12866_2014_214_add10.pdf]
