# Supplementary material for: The characterization and antibiotic resistance profiles of clinical Escherichia coli O25b-B2-ST131 isolates in Kuwait
Source: BMC Microbiol. 2014 Aug 28;14:214. doi: 10.1186/s12866-014-0214-6 (PMC4159528; doi:10.1186/s12866-014-0214-6)

Applied Biosystems

	18_3130POP7_v3.1_2012-11-29	Inst Model/Name 3100/3130GeneticAnalyzer-19348-006
	18	Nov 29,2012 12:43PM, AST
S/N G:269 A:121 T:87 C:129	KB_3130_POP7_BDTv3.mob	Nov 29,2012 12:54PM, AST
KB.bcp	Pts 1711 to 8318 Pk1 Loc:1680	Spacing:9.75
KB 1.4.0 Cap:2	Version 5.3 HiSQV Bases: 186	Plate Name: 29112012

```
  1  TAATTTTTCG CCGCCGCAGC CAGAATATCC CGACGGCTTT CCGCCTTCTG CTCCGGTTGG GTAAAGTAGG   70
 71  TCACCAGAAC CAGCGGTGCG TGGTTTTCCG GCCAGATAAC CGCGATATCG TTGGTGGTGC CATAATCTCC  140
141  GCTGCCGGTT TTATCGCCCA CTACCCATGA TTTCGGCAGA CCCGCCCGAA TGCTCTGAGT CATCATGAAT  210
211  TCCG                                                                          214
```

Applied Biosystems

S/N G:269 A:121 T:87 C:129
KB.bcp
KB 1.4.0 Cap:2

18_3130POP7_v3.1_2012-11-29
18
KB_3130_POP7_BDTv3.mob
Pts 1711 to 8318 Pk1 Loc:1680
Version 5.3 HiSQV Bases: 186

Inst Model/Name 3100/3130GeneticAnalyzer-19348-006
Nov 29,2012 12:43PM, AST
Nov 29,2012 12:54PM, AST
Spacing:9.75 Pts/Panel1500
Plate Name: 29112012

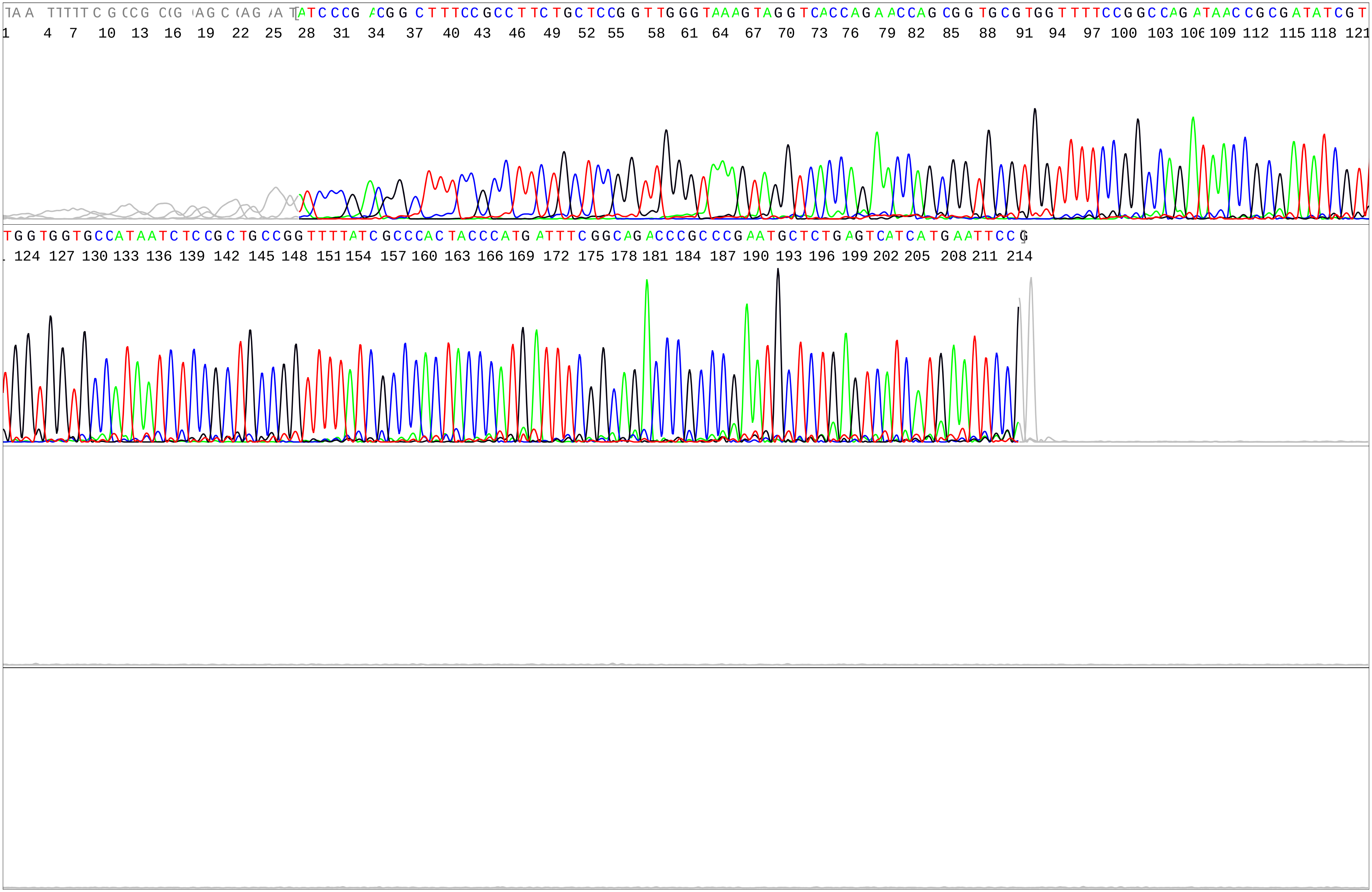

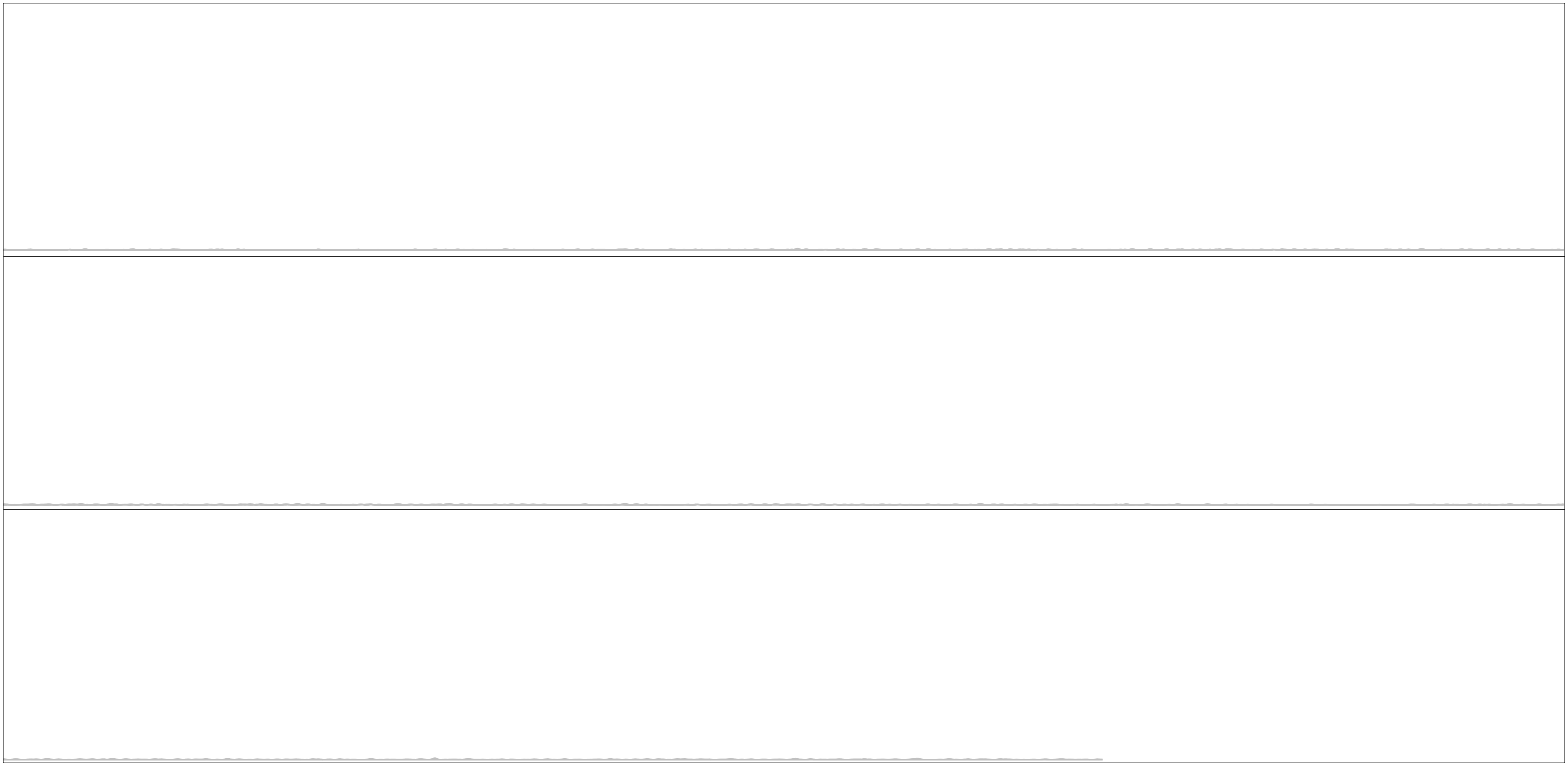

Supplement: Additional file 1: Table S1. — Specimen types and Demographics of E. coli O25b-B2-ST131 isolates. Samples from pus, skin and wound have been illustrated under soft tissue. [file 12866_2014_214_MOESM1_ESM.zip › 12866_2014_214_MOESM1_ESM/12866_2014_214_add11.pdf]
