# Supplementary material for: The characterization and antibiotic resistance profiles of clinical Escherichia coli O25b-B2-ST131 isolates in Kuwait
Source: BMC Microbiol. 2014 Aug 28;14:214. doi: 10.1186/s12866-014-0214-6 (PMC4159528; doi:10.1186/s12866-014-0214-6)

S6_3130POP7_v3.1_2014-03-11
S6
KB_3130_POP7_BDTv3.mob
Pts 1840 to 11071 Pk1 Loc:1817
Version 5.3 HiSQV Bases: 696

Applied Biosystems
S/N G:84 A:37 T:36 C:41
KB.bcp
KB 1.4.0 Cap:2

Inst Model/Name 3100/3130GeneticAnalyzer-19348-006
Mar 11,2014 11:26PM, AST
Mar 11,2014 11:48PM, AST
Spacing:13.42
Plate Name: Febine

1	GGATTAATTT	TCCGCCGCCG	CAGCCAGAAT	ATCCCGACGG	CTTTCCGCCT	TCTGCTCCGG	TTGGGTAAAG	70
71	TAGGTCACCA	GAACCAGCGG	TGCGTGGTTT	TCCGGCCAGA	TAACCGCGAT	ATCGTTGGTG	GTGCCATAAT	140
141	CTCCGCTGCC	GGTTTTATCG	CCCACTACCC	ATGATTTCGG	CAGACCCGCC	CGAATGCTCG	CGCTACCGGT	210
211	AGTATTGCCC	TTAAGCCACG	TCACCAACTG	TGCCCGCTGA	GTTTCCGCCA	GCGCTTTACC	CAGCGTCAGA	280
281	TTTTTCAGGG	TCTGCGCCAT	CGCGAGCGGC	GTGGTGGTAT	CACGCGGGTC	GCCTGGAATG	GCGGTATTGA	350
351	GCGTGGGCTC	GGTTCTGTCC	AGACGGAAGG	TCTCATCACC	CAACGAGCGA	GCAAACGCCG	TCACTTTATC	420
421	GGGACCACCC	AGATGGGCAA	TCAGCTTATT	CATGGCAGTA	TTGTCGCTAT	ACTGCAGCGC	CGCTGCGCCA	490
491	AGCTCAGCCA	GCGTCATCGT	GCCGTTAACG	TGTTTCTCCG	CAATGGGATT	GTAGTTAACC	AGGTCGCTCT	560
561	TCTTGATTTC	AACGCGCTGA	TTTAGCAGGT	GCTTATCGCT	CTCGCTCTGT	TTAAGCACCG	CCGCGGCCGC	630
631	CATCACCTTA	CTGGTACTGC	ACATCGCAAA	ACGTTCATCG	GCACGGTAGA	GAATCTGCGA	ATTATCGGGC	700
701	GGGTGTTAAT	CAGCGCAACG	CCAAGCCGAC	CTTCCC				736

Applied Biosystems

S/N G:84 A:37 T:36 C:41
KB.bcp
KB 1.4.0 Cap:2

S6_3130POP7_v3.1_2014-03-11
S6
KB_3130_POP7_BDTv3.mob
Pts 1840 to 11071 Pk1 Loc:1817
Version 5.3 HiSQV Bases: 696

Inst Model/Name 3100/3130GeneticAnalyzer-19348-006
Mar 11,2014 11:26PM, AST
Mar 11,2014 11:48PM, AST
Spacing:13.42 Pts/Panel1500
Plate Name: Febine

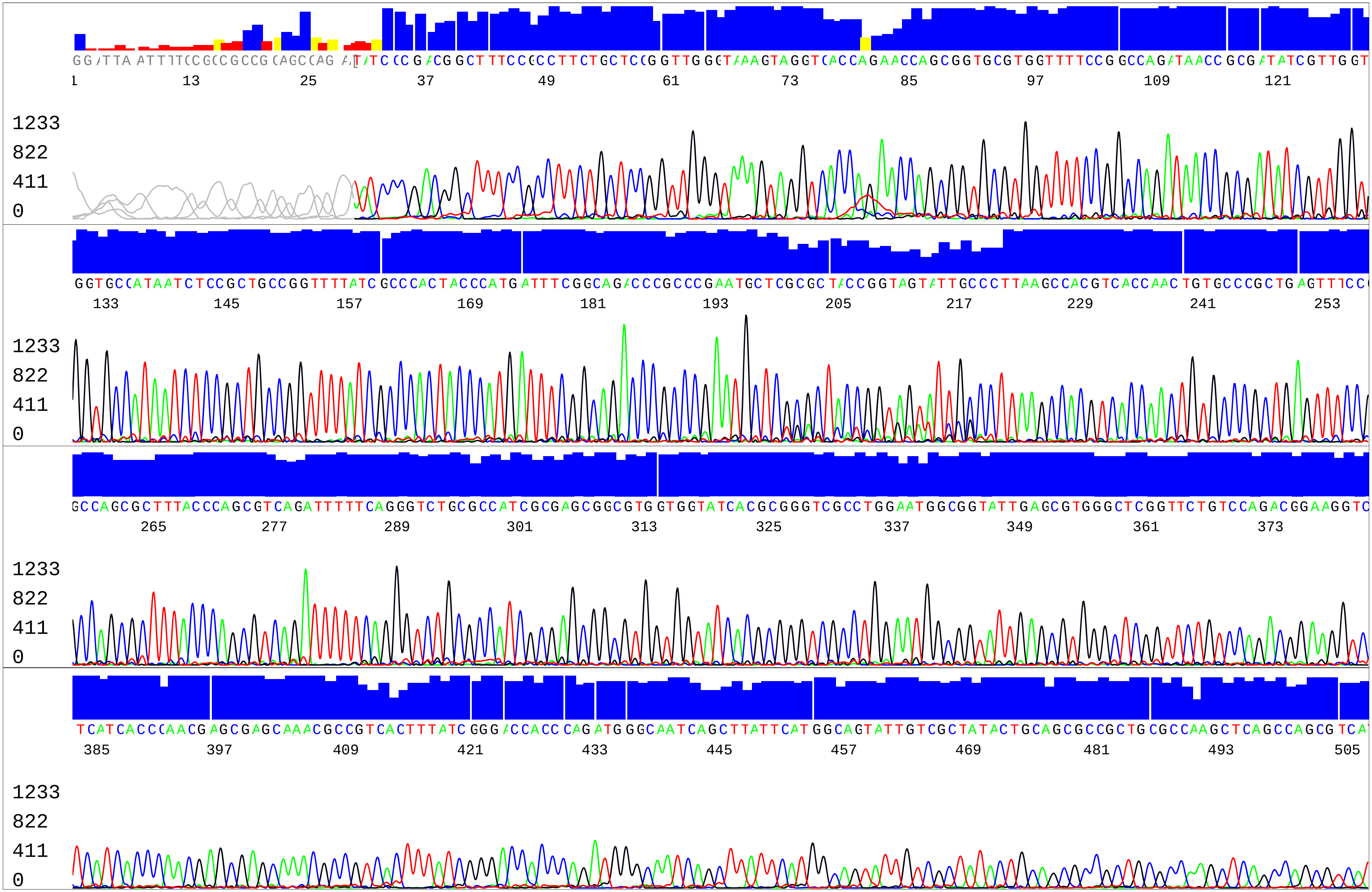

Applied Biosystems

S/N G:84 A:37 T:36 C:41
KB.bcp
KB 1.4.0   Cap:2

S6_3130POP7_v3.1_2014-03-11
S6
KB_3130_POP7_BDTv3.mob
Pts 1840 to 11071 Pk1 Loc:1817
Version 5.3   HiSQV Bases: 696

Inst Model/Name 3100/3130GeneticAnalyzer-19348-006
Mar 11,2014 11:26PM, AST
Mar 11,2014 11:48PM, AST
Spacing:13.42 Pts/Panel1500
Plate Name: Febine

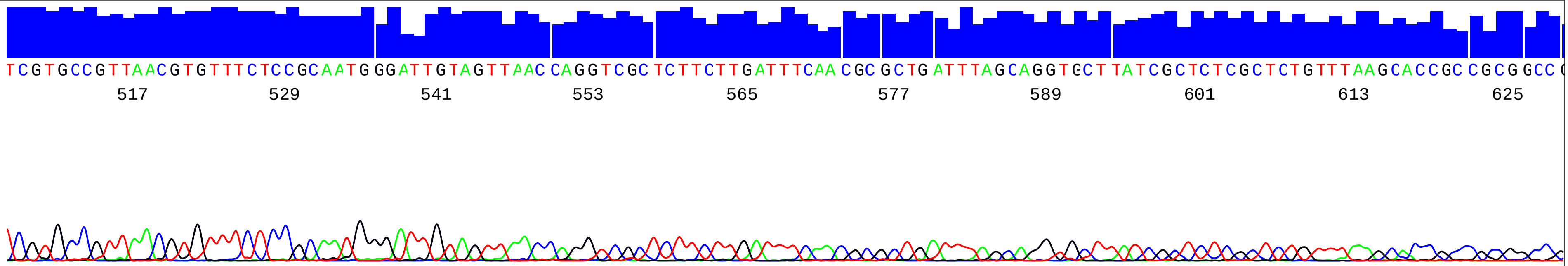

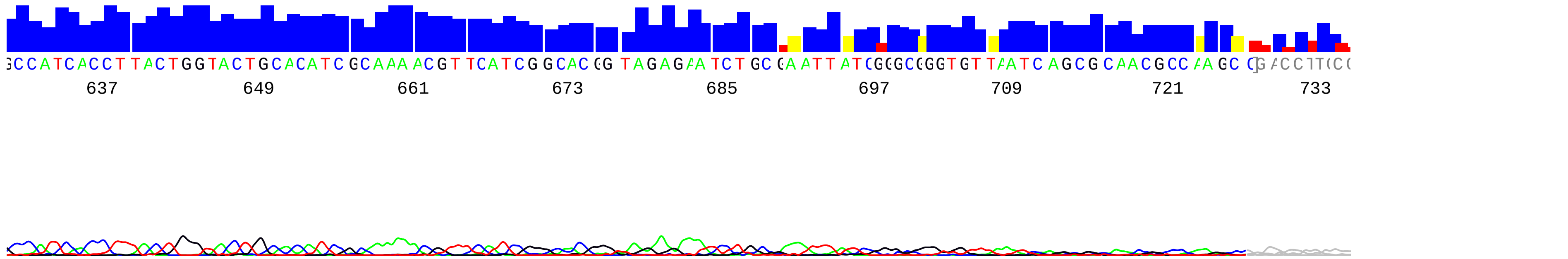

Supplement: Additional file 1: Table S1. — Specimen types and Demographics of E. coli O25b-B2-ST131 isolates. Samples from pus, skin and wound have been illustrated under soft tissue. [file 12866_2014_214_MOESM1_ESM.zip › 12866_2014_214_MOESM1_ESM/12866_2014_214_add13.pdf]
