# Supplementary material for: The characterization and antibiotic resistance profiles of clinical Escherichia coli O25b-B2-ST131 isolates in Kuwait
Source: BMC Microbiol. 2014 Aug 28;14:214. doi: 10.1186/s12866-014-0214-6 (PMC4159528; doi:10.1186/s12866-014-0214-6)

1	GCCCTATGTT	ACGGTGATGG	CGACGCTACC	CCTGCTATTT	AGCAGCGCAA	CGCTGCATGC	GCAGGCGAAC	70
71	AGCGTGCAAC	AGCAGCTGGA	AGCCCTGGAG	AAAAGTTCGG	GAGGTCGGCT	TGGCGTTGCG	CTGATTAACA	140
141	CCGCCGATAA	TTCGCAGATT	CTCTACCGTG	CCGATGAACG	TTTTGCGATG	TGCAGTACCA	GTAAGGTGAT	210
211	GGCGGCCGCG	GCGGTGCTTA	AACAGAGCGA	GAGCGATAAG	CACCTGCTAA	ATCAGCGCGT	TGAAATCAAG	280
281	AAGAGCGACC	TGGTTAACTA	CAATCCCATT	GCGGAGAAAC	ACGTTAACGG	CACGATGACG	CTGGCTGAGC	350
351	TTGGCGCAGC	GGCGCTGCAG	TATAGCGACA	ATACTGCCAT	GAATAAGCTG	ATTGCCCATC	TGGGTGGTCC	420
421	CGATAAAGTG	ACGGCGTTTG	CTCGCTCGTT	GGGTGATGAG	ACCTTCCGTC	TGGACAGAAC	CGAGCCCACG	490
491	CTCAATACCG	CCATTCCAGG	CGACCCGCGT	GATACCACCA	CGCCGCTCGC	GATGGCGCAG	ACCCTGAAAA	560
561	ATCTGACGCT	GGGTAAAGCG	CTGGCGGAAA	CTCAGCGGGC	ACAGTTGGTG	ACGTGGCTTA	AGGGCAATAC	630
631	TACCGGTAGC	GCGAGCATTC	GGGCGGGTCT	GCCGAAATCA	TGGGTAGTGG	GCGATAAAAC	CGGCAGCGGA	700
701	GATTATGGCA	CCACCAACGA	TATCGCGGTT					730

Applied Biosystems

S/N G:459 A:239 T:138 C:193
KB.bcp
KB 1.4.0   Cap:1

S1_3130POP7_v3.1_2014-03-11
S1
KB_3130_POP7_BDTv3.mob
Pts 1835 to 11071 Pk1 Loc:1812
Version 5.3   HiSQV Bases: 710

Inst Model/Name 3100/3130GeneticAnalyzer-19348-006
Mar 11,2014 09:57PM, AST
Mar 11,2014 10:19PM, AST
Spacing:13.25 Pts/Panel1500
Plate Name: Febine

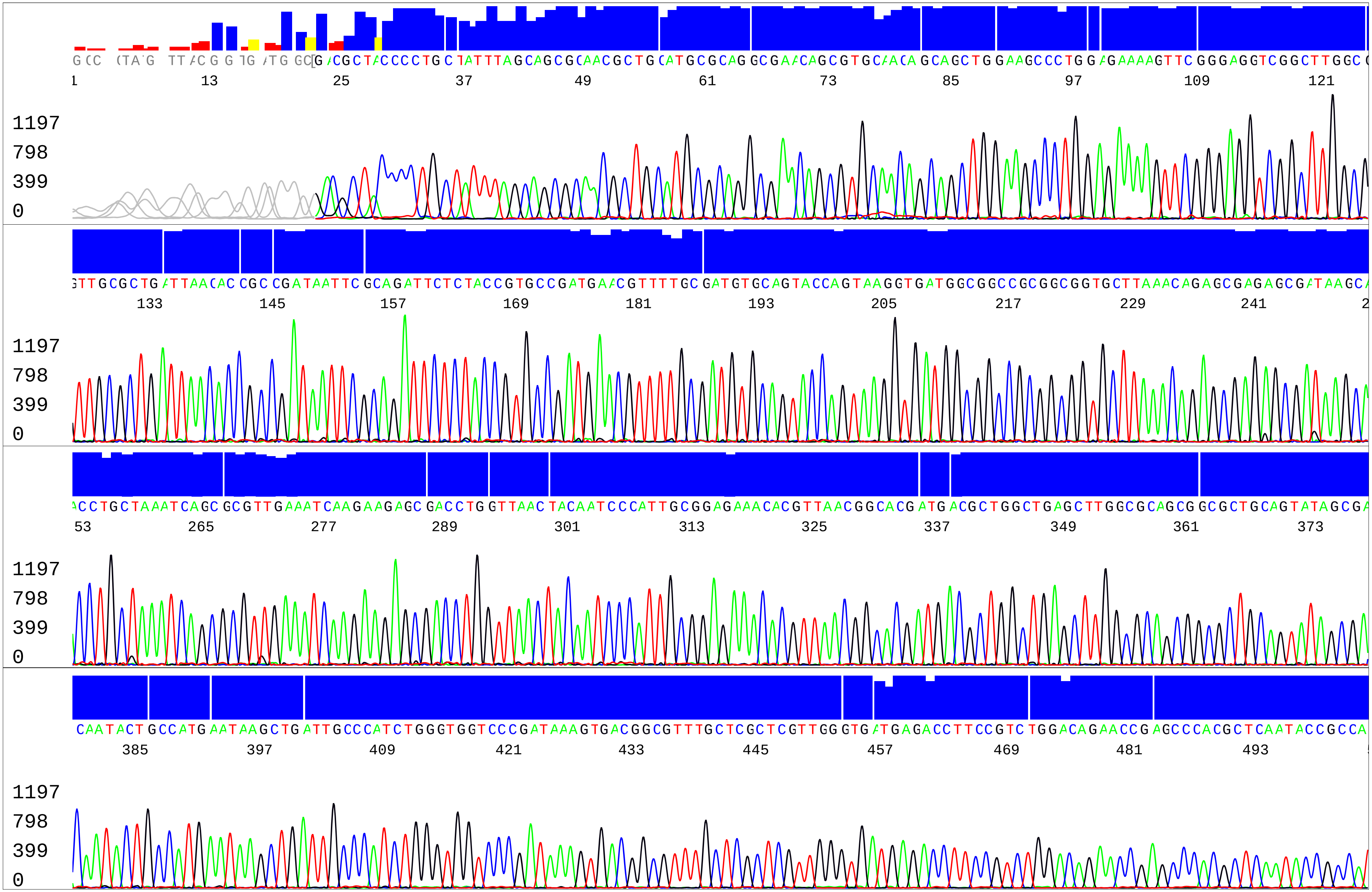

Applied Biosystems

S/N G:459 A:239 T:138 C:193
KB.bcp
KB 1.4.0 Cap:1

Inst Model/Name 3100/3130GeneticAnalyzer-19348-006
Mar 11,2014 09:57PM, AST
Mar 11,2014 10:19PM, AST
Spacing:13.25 Pts/Panel1500
Plate Name: Febine

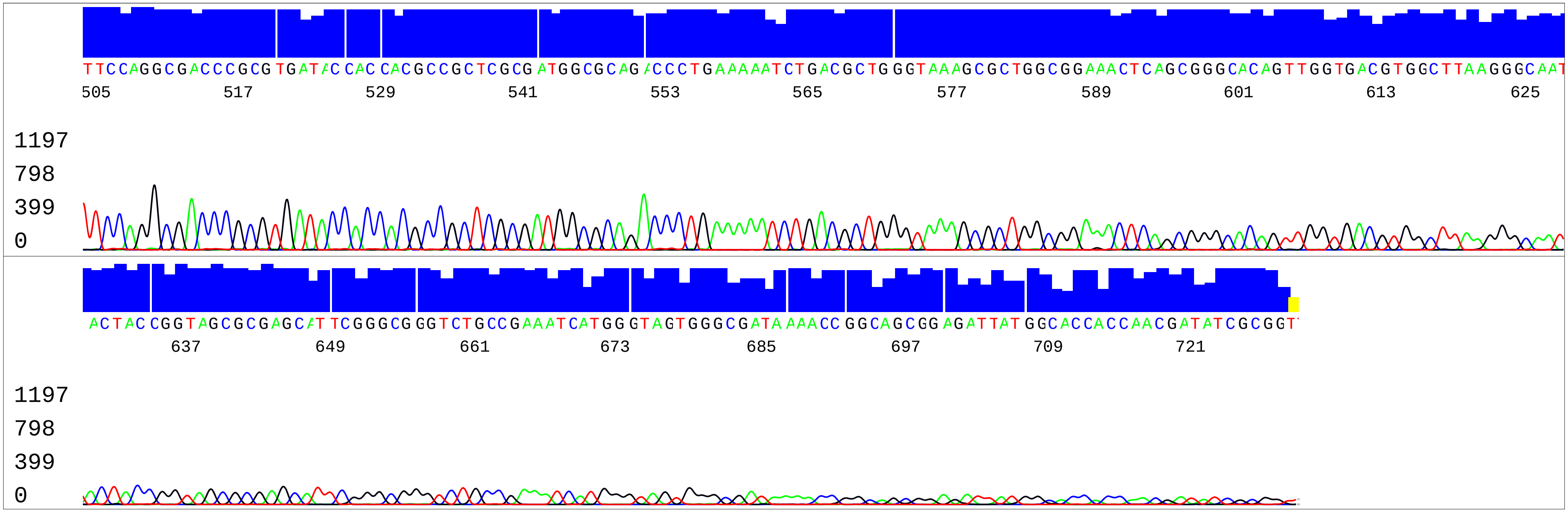

Supplement: Additional file 1: Table S1. — Specimen types and Demographics of E. coli O25b-B2-ST131 isolates. Samples from pus, skin and wound have been illustrated under soft tissue. [file 12866_2014_214_MOESM1_ESM.zip › 12866_2014_214_MOESM1_ESM/12866_2014_214_add14.pdf]
