# Supplementary material for: The characterization and antibiotic resistance profiles of clinical Escherichia coli O25b-B2-ST131 isolates in Kuwait
Source: BMC Microbiol. 2014 Aug 28;14:214. doi: 10.1186/s12866-014-0214-6 (PMC4159528; doi:10.1186/s12866-014-0214-6)

```
  1  GGCAAATTTT CGCGCGCAGC CAGAATATCC CGACGGCTTT CCGCCTTCTG CTCCGGTTGG GTAAAGTAGG   70
 71  TCACCAGAAC CAGCGGTGCG TGGTTTTCCG GCCAGATAAC CGCGATATCG TTGGTGGTGC CATAATCTCC  140
141  GCTGCCGGTT TTATCGCCCA CTACCCATGA TTTCGGCAGA CCCGCCCGAA TGCTCGCGCT ACCGGTAGTA  210
211  TTGCCCTTAA GCCACGTCAC CAACTGTGCC CGCTGAGTTT CCGCCAGCGC TTTACCCAGC GTCAGATTTT  280
281  TCAGGGTCTG CGCCATCGCG AGCGGCGTGG TGGTATCACG CGGGTCGCCT GGAATGGCGG TATTGAGCGT  350
351  GGGCTCGGTT CTGTCCAGAC GGAAGGTCTC ATCACCCAAC GAGCGAGCAA ACGCCGTCAC TTTATCGGGA  420
421  CCACCCAGAT GGGCAATCAG CTTATTCATG GCAGTATTGT CGCTATACTG CAGCGCCGCT GCGCCAAGCT  490
491  CAGCCAGCGT CATCGTGCCG TTAACGTGTT TCTCCGCAAT GGGATTGTAG TTAACCAGGT CGCTCTTCTT  560
561  GATTTCAACG CGCTGATTTA GCAGGTGCTT ATCGCTCTCG CTCTGTTTAA GCACCGCCGC GGCCGCCATC  630
631  ACCTTACTGG TACTGCACAT CGCAAAACGT TCATCGGCAC GGTAGAGAAT CTGCGAATTA TCGGCGGTGT  700
701  TAATCAGCGC AACGCCAAGC CGACCTC                                                 727
```

Applied Biosystems

S/N G:610 A:246 T:259 C:295
KB.bcp
KB 1.4.0 Cap:2

S2_3130POP7_v3.1_2014-03-11
S2
KB_3130_POP7_BDTv3.mob
Pts 1840 to 11071 Pk1 Loc:1817
Version 5.3 HiSQV Bases: 705

Inst Model/Name 3100/3130GeneticAnalyzer-19348-006
Mar 11,2014 09:57PM, AST
Mar 11,2014 10:19PM, AST
Spacing:13.38 Pts/Panel1500
Plate Name: Febine

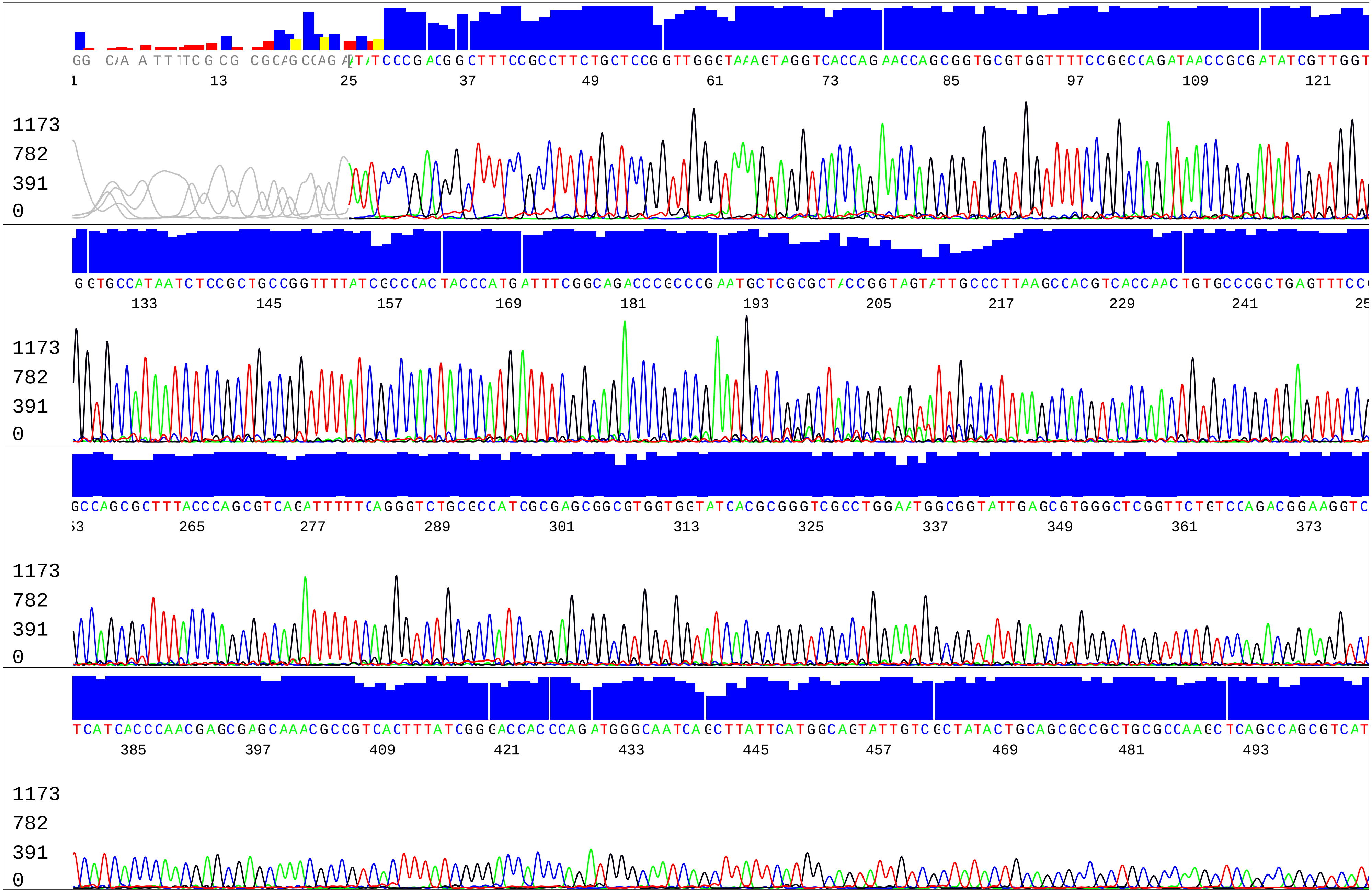

S2_3130POP7_v3.1_2014-03-11
S2
KB_3130_POP7_BDTv3.mob
Pts 1840 to 11071 Pk1 Loc:1817
Version 5.3   HiSQV Bases: 705

Inst Model/Name 3100/3130GeneticAnalyzer-19348-006
Mar 11,2014 09:57PM, AST
Mar 11,2014 10:19PM, AST
Spacing:13.38 Pts/Panel1500
Plate Name: Febine

S/N G:610 A:246 T:259 C:295
KB.bcp
KB 1.4.0   Cap:2

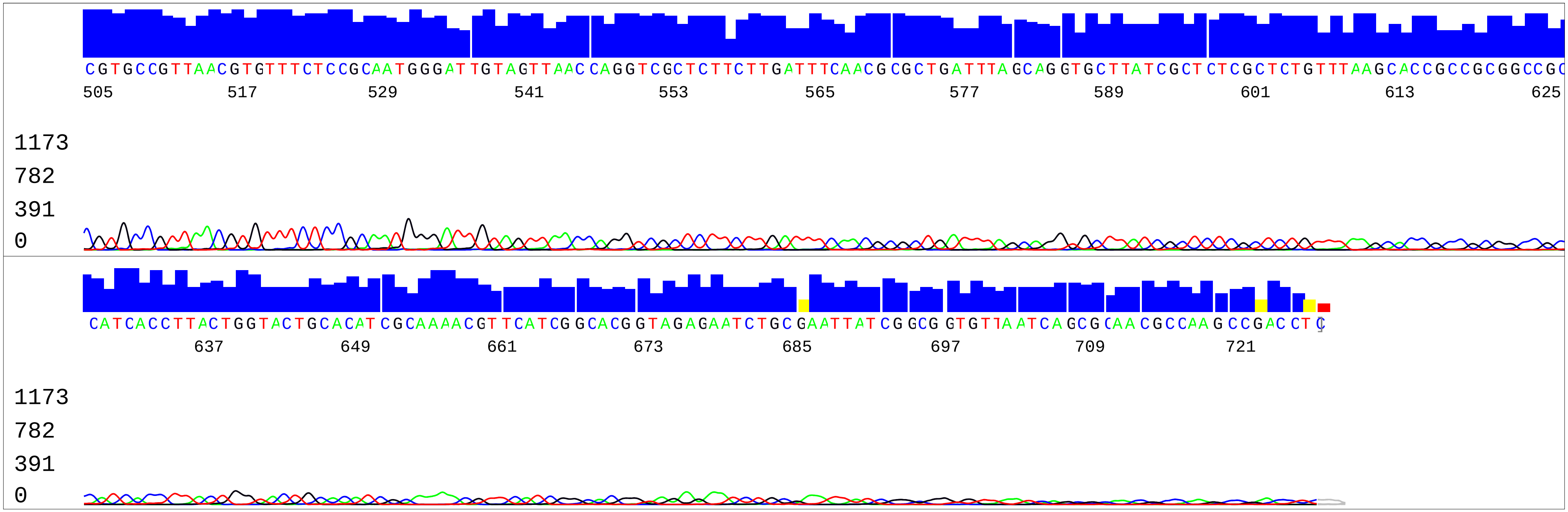

Supplement: Additional file 1: Table S1. — Specimen types and Demographics of E. coli O25b-B2-ST131 isolates. Samples from pus, skin and wound have been illustrated under soft tissue. [file 12866_2014_214_MOESM1_ESM.zip › 12866_2014_214_MOESM1_ESM/12866_2014_214_add15.pdf]
