# Supplementary material for: The characterization and antibiotic resistance profiles of clinical Escherichia coli O25b-B2-ST131 isolates in Kuwait
Source: BMC Microbiol. 2014 Aug 28;14:214. doi: 10.1186/s12866-014-0214-6 (PMC4159528; doi:10.1186/s12866-014-0214-6)

Applied Biosystems

CTXFM-F_3130POP7_v3.1_2012-10-21
CTXFM-F
KB_3130_POP7_BDTv3.mob
Pts 1813 to 8532 Pk1 Loc:1782
Version 5.3 HiSQV Bases: 557

Inst Model/Name 3100/3130GeneticAnalyzer-19348-006
Oct 21,2012 04:06PM, AST
Oct 21,2012 04:17PM, AST
Spacing:10.55
Plate Name: SS-21102012

S/N G:171 A:92 T:54 C:76
KB.bcp
KB 1.4.0 Cap:3

1	GCGCCCGGGC	CGGCCGGTTC	TTGAAGAAAA	GTGAAAGCGA	ACCGGAATCT	GTTAAATCAG	CGAGTTGAGA	70
71	TCAAAAAATC	TGACCTTGTT	AACTATAATC	CGATTGCGGA	AAAGCACGTC	AATGGGACGA	TGTCACTGGC	140
141	TGAGCTTAGC	GCGGCCGCGC	TACAGTACAG	CGATAACGTG	GCGATGAATA	AGCTGATTGC	TCACGTTGGC	210
211	GGCCCGGCTA	GCGTCACCGC	GTTCGCCCGA	CAGCTGGGAG	ACGAAACGTT	CCGTCTCGAC	CGTACCGAGC	280
281	CGACGTTAAA	CACCGCCATT	CCGGGCGATC	CGCGTGATAC	CACTTCACCT	CGGGCAATGG	CGCAAACTCT	350
351	GCGGAATCTG	ACGCTGGGTA	AAGCATTGGG	CGACAGCCAA	CGGGCGCAGC	TGGTGACATG	GATGAAAGGC	420
421	AATACCACCG	GTGCAGCGAG	CATTCAGGCT	GGACTGCCTG	CTTCCTGGGT	TGTGGGGGAT	AAAACCGGCA	490
491	GCGGTGGCTA	TGGCACCACC	AACGTTATCG	CGGTGCAGGA	CGCGCCGCGC	TCAGCTCAGC	CAGTGACATC	560
561	GTCCCATTGA	CGTGCTTTTC	CGCACTCGGA	TTATAGTTGA	ACAGGGTCAG	ATTTTTTGAT	CTCAACTCCG	630
631	CTGATGTTAA	CAGATTCGGT	TCGATTTTCA	CTTTTTCCTT	CAGCAACCGC	GGACCCGCGG	ACCTGTTAAC	700
701	C							701

CTXFM-F_3130POP7_v3.1_2012-10-21
CTXFM-F
KB_3130_POP7_BDTv3.mob
Pts 1813 to 8532 Pk1 Loc:1782
Version 5.3 HiSQV Bases: 557

Inst Model/Name 3100/3130GeneticAnalyzer-19348-006
Oct 21,2012 04:06PM, AST
Oct 21,2012 04:17PM, AST
Spacing:10.55 Pts/Panel1500
Plate Name: SS-21102012

S/N G:171 A:92 T:54 C:76
KB.bcp
KB 1.4.0 Cap:3

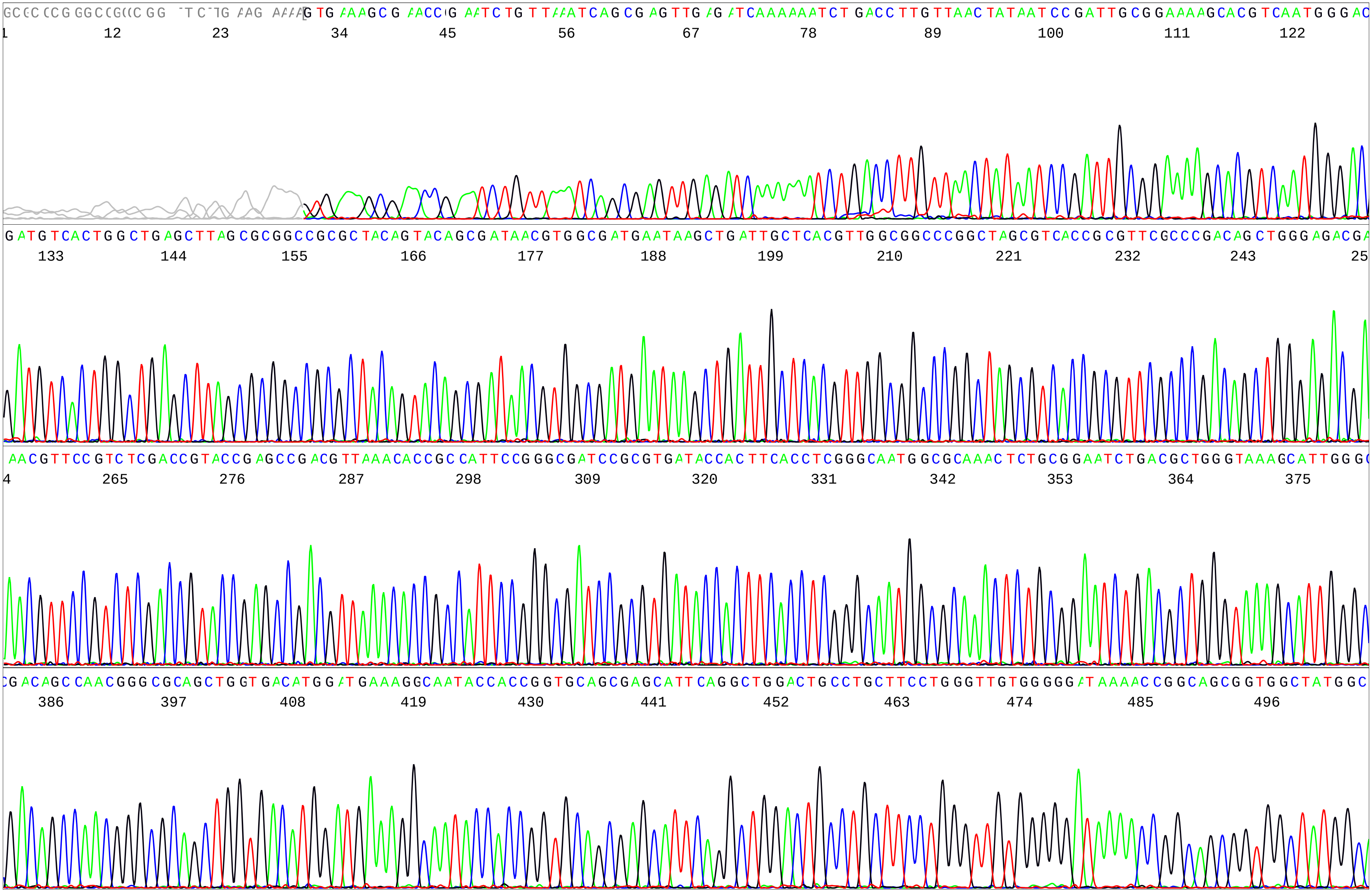

CTXFM-F_3130POP7_v3.1_2012-10-21
CTXFM-F
KB_3130_POP7_BDTv3.mob
Pts 1813 to 8532 Pk1 Loc:1782
Version 5.3 HiSQV Bases: 557

Inst Model/Name 3100/3130GeneticAnalyzer-19348-006
Oct 21,2012 04:06PM, AST
Oct 21,2012 04:17PM, AST
Spacing:10.55 Pts/Panel1500
Plate Name: SS-21102012

S/N G:171 A:92 T:54 C:76
KB.bcp
KB 1.4.0 Cap:3

ACCACCAACG]TTATCGCGGTGCAGGA CGCGCCGCG CTCAGCTCAGCCAG TGACATCGTCCCATTG AC GTGCTTTTCCGC/C TC G GATT ATAGTTGAACAGG G TCAG AT TTTTT TGATC

507 518 529 540 551 562 573 584 595 606 617

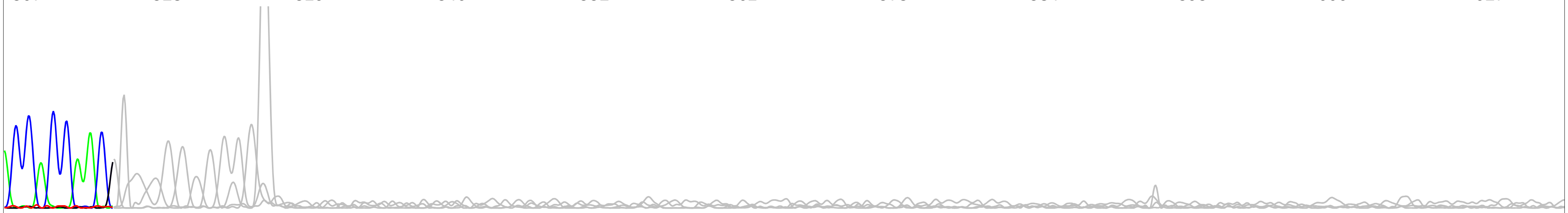

TCA ACTCCG CTGATGTT AAC A GA TTCG GT TCG ATTTTC /C TTTTTCC TT CAG CAAC CG CGG ACCCG C CGAC CTG TT AAC C

628 639 650 661 672 683 694

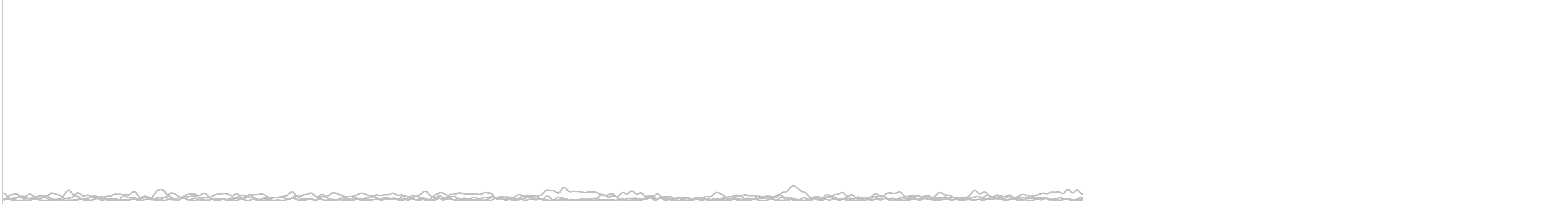

Supplement: Additional file 1: Table S1. — Specimen types and Demographics of E. coli O25b-B2-ST131 isolates. Samples from pus, skin and wound have been illustrated under soft tissue. [file 12866_2014_214_MOESM1_ESM.zip › 12866_2014_214_MOESM1_ESM/12866_2014_214_add16.pdf]
