# Supplementary material for: The characterization and antibiotic resistance profiles of clinical Escherichia coli O25b-B2-ST131 isolates in Kuwait
Source: BMC Microbiol. 2014 Aug 28;14:214. doi: 10.1186/s12866-014-0214-6 (PMC4159528; doi:10.1186/s12866-014-0214-6)

Applied Biosystems

CTXFM-R_3130POP7_v3.1_2012-10-21
CTXFM-R
KB_3130_POP7_BDTv3.mob
Pts 1852 to 8532 Pk1 Loc:1821
Version 5.3   HiSQV Bases: 498

Inst Model/Name 3100/3130GeneticAnalyzer-19348-006
Oct 21,2012 04:06PM, AST
Oct 21,2012 04:17PM, AST
Spacing:10.54
Plate Name: SS-21102012

S/N G:1097 A:626 T:452 C:606
KB.bcp
KB 1.4.0   Cap:4

1	CCGTCGGCTA	TCCCCACACC	CAGGAGCAGG	CAGTCCAGCC	TGAATGCTCG	CTGCACCGGT	GGTATTGCCT	70
71	TTCATCCATG	TCACCAGCTG	CGCCCGTTGG	CTGTCGCCCA	ATGCTTTACC	CAGCGTCAGA	TTCCGCAGAG	140
141	TTTGCGCCAT	TGCCCGAGGT	GAAGTGGTAT	CACGCGGATC	GCCCGGAATG	GCGGTGTTTA	ACGTCGGCTC	210
211	GGTACGGTCG	AGACGGAACG	TTTCGTCTCC	CAGCTGTCGG	GCGAACGCGG	TGACGCTAGC	CGGGCCGCCA	280
281	ACGTGAGCAA	TCAGCTTATT	CATCGCCACG	TTATCGCTGT	ACTGTAGCGC	GGCCGCGCTA	AGCTCAGCCA	350
351	GTGACATCGT	CCCATTGACG	TGCTTTTCCG	CAATCGGATT	ATAGTTAACA	AGGTCAGATT	TTTTGATCTC	420
421	AACTCGCTGA	TTTAACAGAT	TCGGTTCGCT	TTCACTTTTC	TTCAGCACCG	CGGCCGCGGC	CATCACTTTA	490
491	CTGGTGCTGA	ACCCCCCAAA	AAGGGAAGCA	ATCTGTTAAA	TCAGCGAGCT	GGGCCCCAAA	GCTGCCCTAG	560
561	TGTTGTATAA	TTCCCAACCC	TCAAAATCAT	AAAATGACAG	AGTTAGACTT	GTATGATCTC	AACTCGCCTG	630
631	ATTTAACGAC	ATTCGGGTGC	GAATTTCCCT	TTTCTTCTGC	ACCGCCGGCC	CCCGGCTGTC	CA	692

CTXFM-R_3130POP7_v3.1_2012-10-21
CTXFM-R
KB_3130_POP7_BDTv3.mob
Pts 1852 to 8532 Pk1 Loc:1821
Version 5.3 HiSQV Bases: 498

Inst Model/Name 3100/3130GeneticAnalyzer-19348-006
Oct 21,2012 04:06PM, AST
Oct 21,2012 04:17PM, AST
Spacing:10.54 Pts/Panel1500
Plate Name: SS-21102012

S/N G:1097 A:626 T:452 C:606
KB.bcp
KB 1.4.0 Cap:4

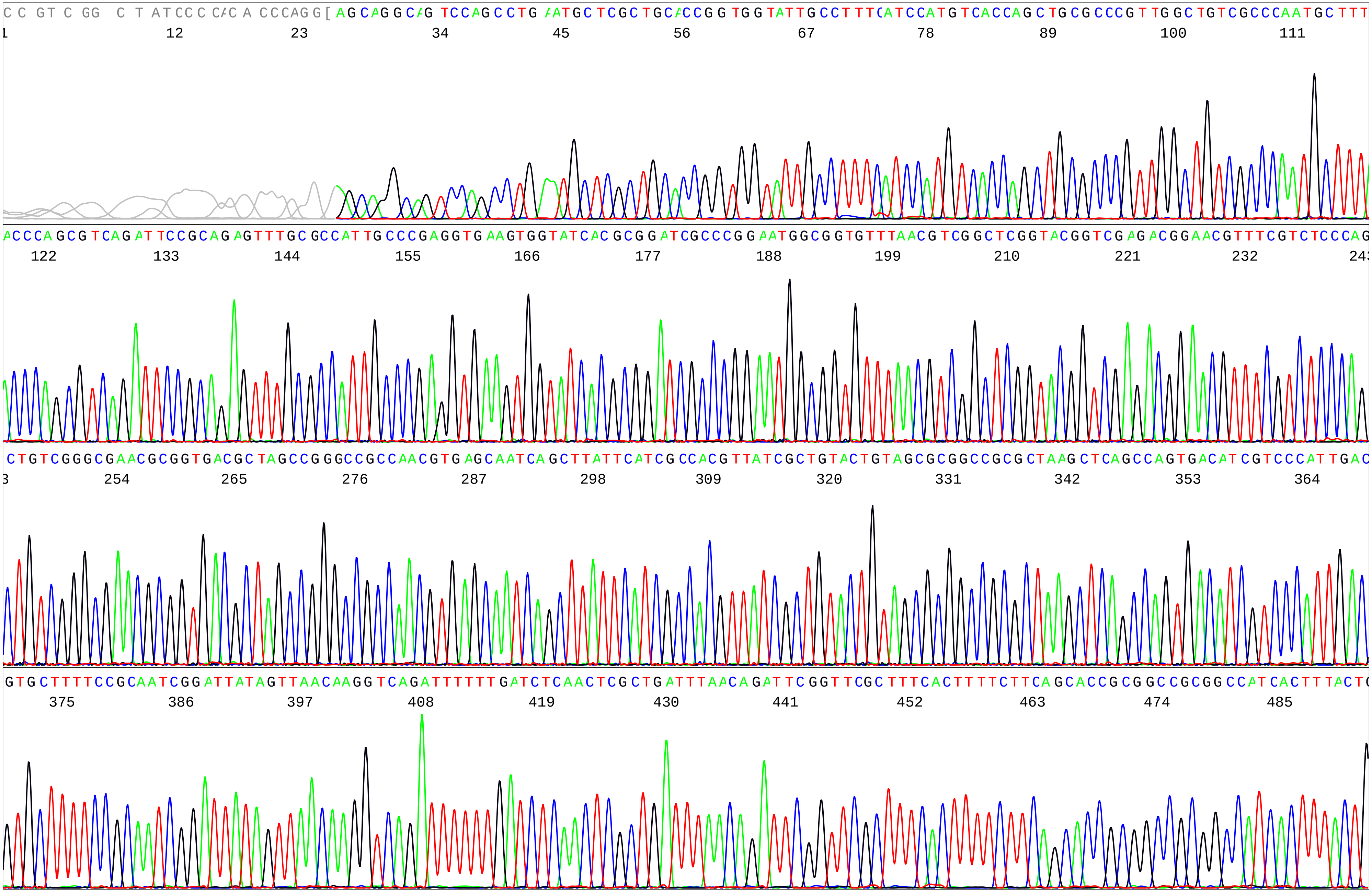

S/N G:1097 A:626 T:452 C:606
KB.bcp
KB 1.4.0   Cap:4

CTXFM-R_3130POP7_v3.1_2012-10-21
CTXFM-R
KB_3130_POP7_BDTv3.mob
Pts 1852 to 8532 Pk1 Loc:1821
Version 5.3   HiSQV Bases: 498

Inst Model/Name 3100/3130GeneticAnalyzer-19348-006
Oct 21,2012 04:06PM, AST
Oct 21,2012 04:17PM, AST
Spacing:10.54 Pts/Panel1500
Plate Name: SS-21102012

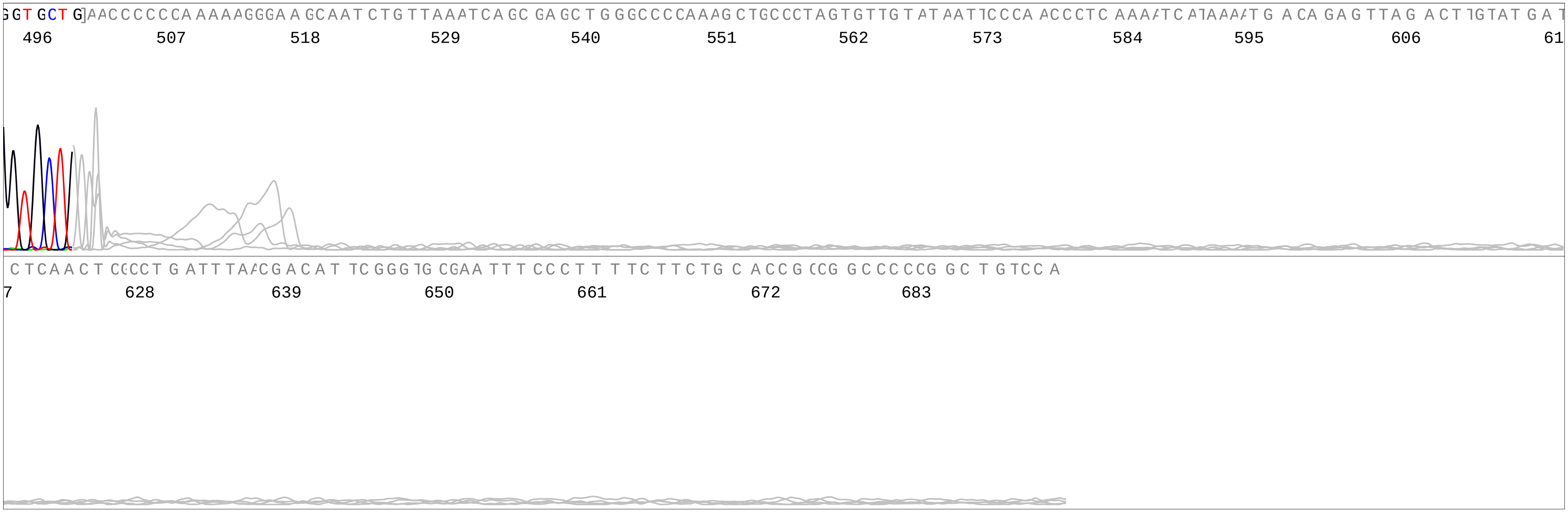

Supplement: Additional file 1: Table S1. — Specimen types and Demographics of E. coli O25b-B2-ST131 isolates. Samples from pus, skin and wound have been illustrated under soft tissue. [file 12866_2014_214_MOESM1_ESM.zip › 12866_2014_214_MOESM1_ESM/12866_2014_214_add17.pdf]
