# Supplementary material for: The characterization and antibiotic resistance profiles of clinical Escherichia coli O25b-B2-ST131 isolates in Kuwait
Source: BMC Microbiol. 2014 Aug 28;14:214. doi: 10.1186/s12866-014-0214-6 (PMC4159528; doi:10.1186/s12866-014-0214-6)
Supplement: Additional file 1: Table S1. — Specimen types and Demographics of E. coli O25b-B2-ST131 isolates. Samples from pus, skin and wound have been illustrated under soft tissue. [file 12866_2014_214_MOESM1_ESM.zip › 12866_2014_214_MOESM1_ESM/12866_2014_214_add18.pdf]

Applied Biosystems

| | | |
|---|---|---|
| | K10F_3130POP7_v3.1_2013-06-04 | Inst Model/Name 3100/3130GeneticAnalyzer-19348-006 |
| | K10F | Jun 04,2013 12:28PM, AST |
| S/N G:580 A:367 T:193 C:348 | KB_3130_POP7_BDTv3.mob | Jun 04,2013 01:00PM, AST |
| KB.bcp | Pts 1787 to 11071 Pk1 Loc:1764 | Spacing:11.28 |
| KB 1.4.0 Cap:3 | Version 5.3 HiSQV Bases: 726 | Plate Name: Febine04.06.2013 |

| | | | | | | | | |
|---|---|---|---|---|---|---|---|---|
| 1 | GCCAGCTTAC | GGTGATGGCG | ACGCTACCCC | TGCTATTTAG | CAGCGCAACG | CTGCATGCGC | AGGCGAACAG | 70 |
| 71 | CGTGCAACAG | CAGCTGGAAG | CCCTGGAGAA | AAGTTCGGGA | GGTCGGCTTG | GCGTTGCGCT | GATTAACACC | 140 |
| 141 | GCCGATAATT | CGCAGATTCT | CTACCGTGCC | GATGAACGTT | TTGCGATGTG | CAGTACCAGT | AAGGTGATGG | 210 |
| 211 | CGGCCGCGGC | GGTGCTTAAA | CAGAGCGAGG | GCGATAAGCA | CCTGCTAAAT | CAGCGCGTTG | AAATCAAGAA | 280 |
| 281 | GAGCGACCTG | GTTAACTACA | ATCCCATTGC | GGAGAAACAC | GTTAACGGCA | CGATGACGCT | GGCTGAGCTT | 350 |
| 351 | GGCGCAGCGG | CGCTGCAGTA | TAGCGACAAT | ACTGCCATGA | ATAAGCTGAT | TGCCCATCTG | GGTGGTCCCG | 420 |
| 421 | ATAAAGTGAC | GGCGTTTGCT | CGCTCGTTGG | GTGATGAGAC | CTTCCGTCTG | GACAGAACCG | AGCCCACGCT | 490 |
| 491 | CAATACCGCC | ATTCCAGGCG | ACCCGCGTGA | TACCACCACG | CCGCTCGCGA | TGGCGCAGAC | CCTGAAAAAT | 560 |
| 561 | CTGACGCTGG | GTAAAGCGCT | GGCGGAAACT | CAGCGGGCAC | AGTTGGTGAC | GTGGCTTAAG | GGCAATACTA | 630 |
| 631 | CCGGTAGCGC | GAGCATTCGG | GCGGGTCTGC | CGAAATCATG | GGTAGTGGGC | GATAAAACCG | GCAGCGGAGA | 700 |
| 701 | TTATGGCACC | ACCAACGATA | TCGCGGTTAT | CTGGGCGGAA | AACCACCCAC | CGGTGGTTCT | GGCGACCTAT | 770 |
| 771 | TTTACACCAG | CAGCAGCAGA | GGGGAAAACA | CCCCTCGATA | TTCTGGGTTT | GCCCGCCGAA | AATTTTGACC | 840 |
| 841 | CCCGGGTATG | GATGCATAAA | CAC | | | | | 863 |

Applied Biosystems

K10F_3130POP7_v3.1_2013-06-04
K10F
KB_3130_POP7_BDTv3.mob
Pts 1787 to 11071 Pk1 Loc:1764
Version 5.3    HiSQV Bases: 726

Inst Model/Name 3100/3130GeneticAnalyzer-19348-006
Jun 04,2013 12:28PM, AST
Jun 04,2013 01:00PM, AST
Spacing:11.28  Pts/Panel1500
Plate Name: Febine04.06.2013

S/N G:580 A:367 T:193 C:348
KB.bcp
KB 1.4.0    Cap:3

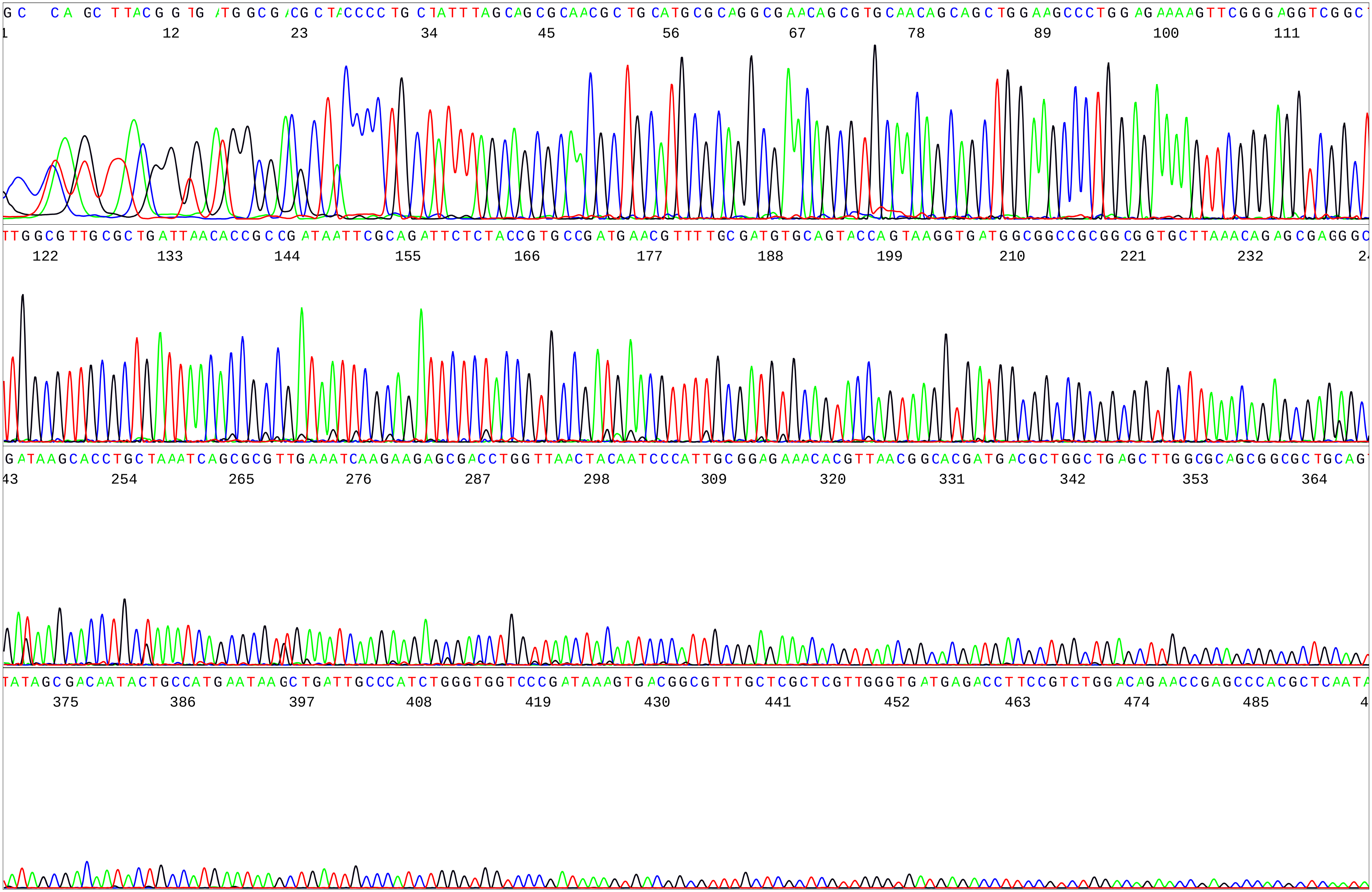

Applied Biosystems

S/N G:580 A:367 T:193 C:348
KB.bcp
KB 1.4.0 Cap:3

K10F_3130POP7_v3.1_2013-06-04
K10F
KB_3130_POP7_BDTv3.mob
Pts 1787 to 11071 Pk1 Loc:1764
Version 5.3 HiSQV Bases: 726

Inst Model/Name 3100/3130GeneticAnalyzer-19348-006
Jun 04,2013 12:28PM, AST
Jun 04,2013 01:00PM, AST
Spacing:11.28 Pts/Panel1500
Plate Name: Febine04.06.2013

CCGCCATTCCAGGCGACCCGCGTGATACCACCACGCCGCTCGCGATGGCGCAGACCCTGAAAAATCTGACGCTGGGTAAAGCGCTGGCGGAAACTCAGCGGGCACAGTTGGTGACGTGGCTTAA

96 507 518 529 540 551 562 573 584 595 606 617

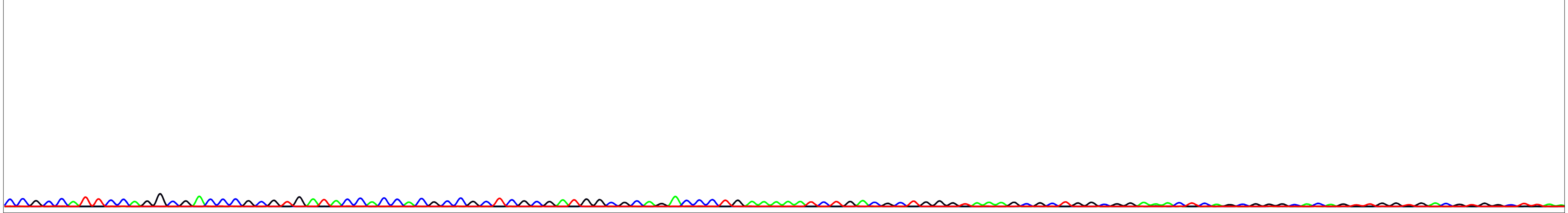

AGGGCAATACTACCGGTAGCGCGAGCATTCGGGCGGGTCTGCCGAAATCATGGGTAGTGGGCGATAAAACCGGCAGCGGAGATTATGGCACCACCAACGATATCGCGGTTATCTGGGCGGA

628 639 650 661 672 683 694 705 716 727 738

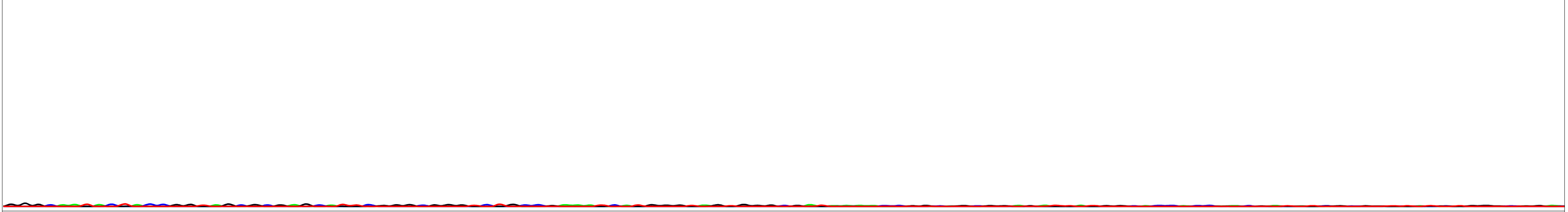

AAACCACCCACCGGTGGTTCTGGCGACCTATTTTACACCAGCAGCAGCAGAGGGGAAAACACCCCTCGATATTCTGGGTTTGCCCGCCGAAAATTTTGACCCCCGGGTATGG/TGCATAAA

749 760 771 782 793 804 815 826 837 848 859

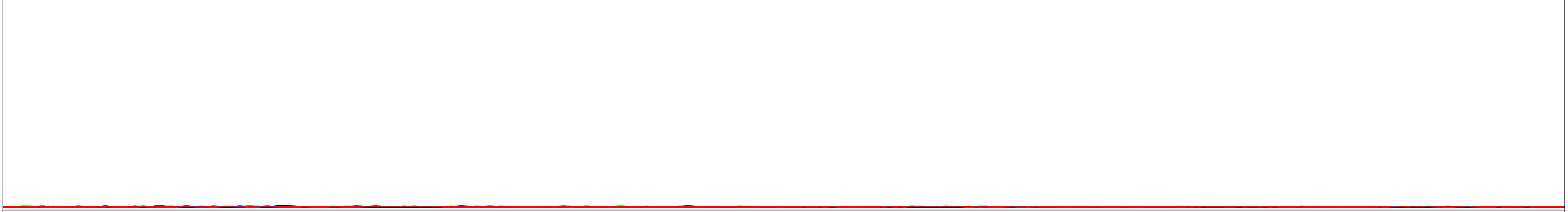

ACAC
