# Supplementary material for: The characterization and antibiotic resistance profiles of clinical Escherichia coli O25b-B2-ST131 isolates in Kuwait
Source: BMC Microbiol. 2014 Aug 28;14:214. doi: 10.1186/s12866-014-0214-6 (PMC4159528; doi:10.1186/s12866-014-0214-6)

Applied Biosystems

S/N G:197 A:123 T:152 C:204	K10R_3130POP7_v3.1_2013-06-04	Inst Model/Name 3100/3130GeneticAnalyzer-19348-006
	K10R	Jun 04,2013 12:28PM, AST
	KB_3130_POP7_BDTv3.mob	Jun 04,2013 01:00PM, AST
KB.bcp	Pts 1802 to 11071 Pk1 Loc:1779	Spacing:11.45
KB 1.4.0 Cap:4	Version 5.3 HiSQV Bases: 772	Plate Name: Febine04.06.2013

```
  1  GGTCGATTTT CGCGCCGCAG CCAGAATATC CCGACGGCTT TCCGCCTTCT GCTCCGGTTG GGTAAAGTAG   70
 71  GTGAAGGCCA GCGGTGCGTG GTTTTCCGGC CAGATAACCG CGAAATCGTT GGTGGTGCCA TAATCTCCGC  140
141  TGCCGGTTTT ATCGCCCACT ACCCATGATT TCGGCAGACC CGCCCGAATG CTCGCGCTAC CGGTAGTATT  210
211  GCCCTTAAGC CACGTCACCA ACTGTGCCCG CTGAGTTTCC GCCAGCGCTT TACCCAGCGT CAGATTTTTC  280
281  AGGGTCTGCG CCATCGCGAG CGGCGTGGTG GTATCACGCG GGTCGCCTGG AATGGCGGTA TTGAGCGTGG  350
351  GCTCGGTTCT GTCCAGACGG AAGGTCTCAT CACCCAACGA GCGAGCAAAC GCCGTCACTT TATCGGGACC  420
421  ACCCAGATGG GCAATCAGCT TATTCATGGC AGTATTGTCG CTATACTGCA GCGCCGCTGC GCCAAGCTCA  490
491  GCCAGCGTCA TCGTGCCGTT AACGTGTTTC TCCGCAATGG GATTGTAGTT AACCAGGTCG CTCTTCTTGA  560
561  TTTCAACGCG CTGATTTAGC AGGTGCTTAT CGCTCTCGCT CTGTTTAAGC ACCGCCGCGG CCGCCATCAC  630
631  CTTACTGGTA CTGCACATCG CAAAACGTTC ATCGGCACGG TAGAGAATCT GCGAATTATC GGCGGTGTTA  700
701  ATCAGCGCAA CGCCAAGCCG ACCTCCCGAA CTTTTCTCCA GGGCTTCCAG CTGCTGTTGC ACGCTGTTCG  770
771  CCTGCGCATG CAGCGTTGCG CTGCTAAATA GGCAGGGGGT AGCGTCGCCA TCACCCGATT AACATTGGAG  840
841  CCGGGCGAAT GCTCTG                                                               856
```

Applied Biosystems

S/N G:197 A:123 T:152 C:204
KB.bcp
KB 1.4.0   Cap:4

K10R_3130POP7_v3.1_2013-06-04
K10R
KB_3130_POP7_BDTv3.mob
Pts 1802 to 11071 Pk1 Loc:1779
Version 5.3   HiSQV Bases: 772

Inst Model/Name 3100/3130GeneticAnalyzer-19348-006
Jun 04,2013 12:28PM, AST
Jun 04,2013 01:00PM, AST
Spacing:11.45 Pts/Panel1500
Plate Name: Febine04.06.2013

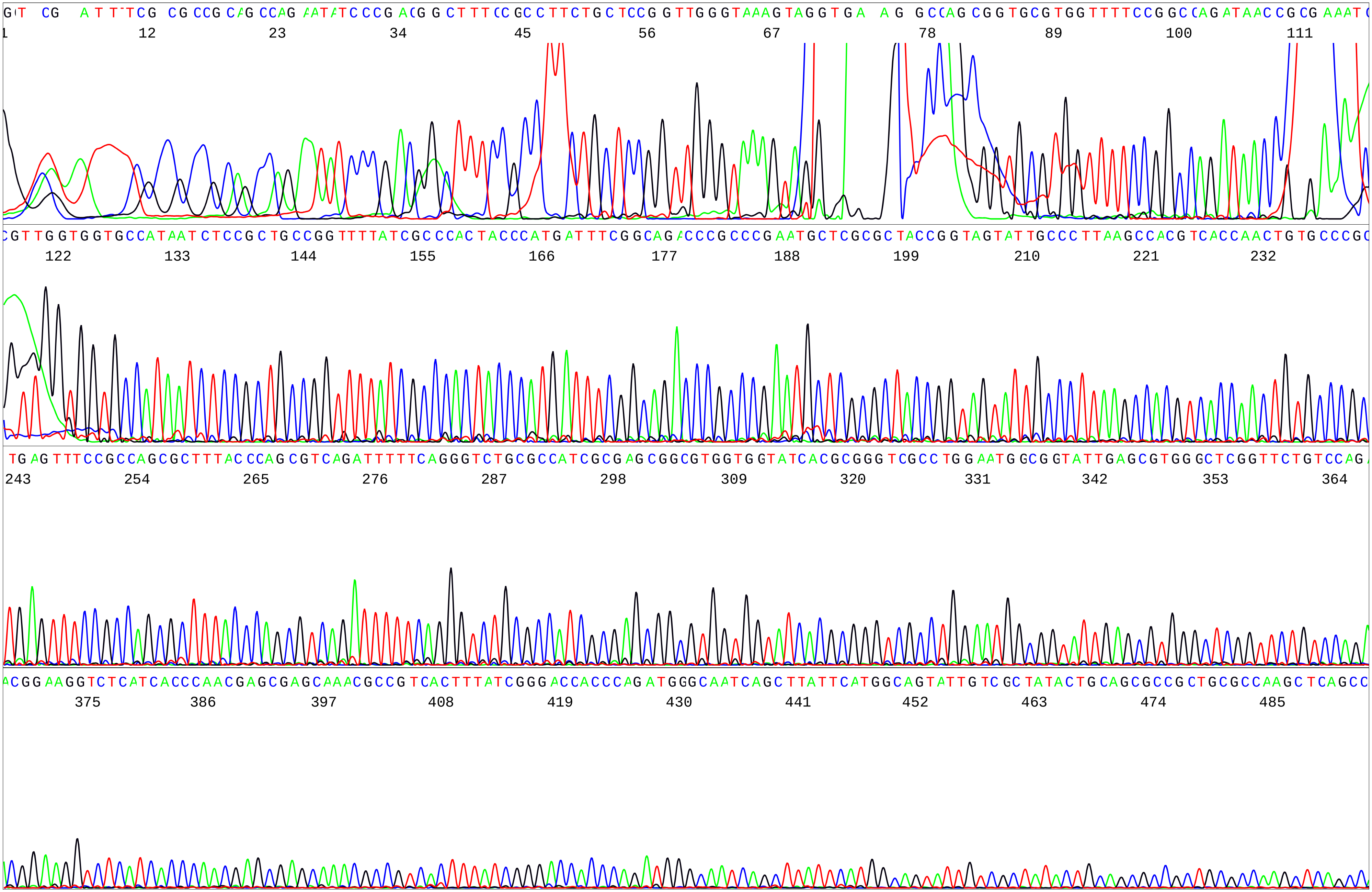

AGCGTCATCGTGCCGTTAACGTGTTTCTCCGCAATGGGATTGTAGTTAACCAGGTCGCTCTTCTTGATTTCAACGCGCTGATTTAGCAGGTGCTTATCGCTCTCGCTCTGTTTAAGCACCGCCG
496 507 518 529 540 551 562 573 584 595 606 617

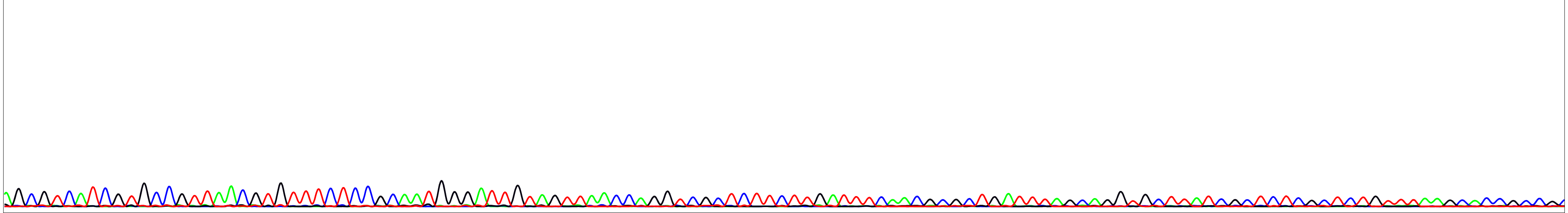

CGGCCGCCATCACCTTACTGGTACTGCACATCGCAAAACGTTCATCGGCACGGTAGAGAATCTGCGAATTATCGGCGGTGTTAATCAGCGCAACGCCAAGCCGACCTCCCGAACTTTTCTC
628 639 650 661 672 683 694 705 716 727 738

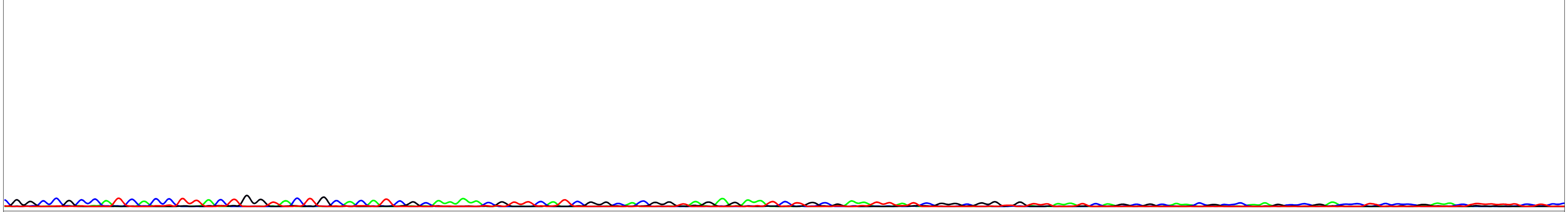

CAGCGCTTCCAGCTGCTGTTGCACGCTGTTCGCCTGCGCATGCAGCGTTGCGCTGCTAAATAGCCACGGGGTAGCGTCGCCATCACCCGATTAACATTCGACCGGCCGAATGCTCTG
749 760 771 782 793 804 815 826 837 848

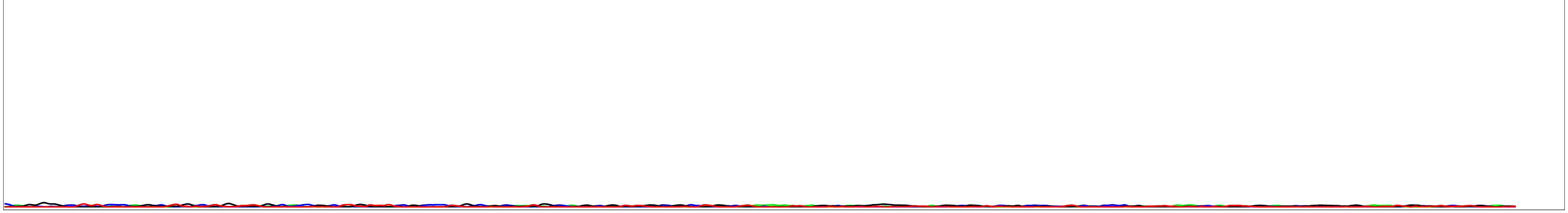

Supplement: Additional file 1: Table S1. — Specimen types and Demographics of E. coli O25b-B2-ST131 isolates. Samples from pus, skin and wound have been illustrated under soft tissue. [file 12866_2014_214_MOESM1_ESM.zip › 12866_2014_214_MOESM1_ESM/12866_2014_214_add19.pdf]
