# Supplementary material for: The characterization and antibiotic resistance profiles of clinical Escherichia coli O25b-B2-ST131 isolates in Kuwait
Source: BMC Microbiol. 2014 Aug 28;14:214. doi: 10.1186/s12866-014-0214-6 (PMC4159528; doi:10.1186/s12866-014-0214-6)

Applied Biosystems

S/N G:3311 A:2149 T:1271 C:1577	121-F_3130POP7_v3.1_2012-10-21	Inst Model/Name 3100/3130GeneticAnalyzer-19348-006
	121-F	Oct 21,2012 04:06PM, AST
	KB_3130_POP7_BDTv3.mob	Oct 21,2012 04:17PM, AST
KB.bcp	Pts 1967 to 8532 Pk1 Loc:1936	Spacing:10.59
KB 1.4.0 Cap:1	Version 5.3 HiSQV Bases: 653	Plate Name: SS-21102012

1	CCTTTTCCTG	TTTTTTTGCT	CACCCAGAAA	CGCTGGTGAA	AGTAAAAGAT	GCTGAAGATC	AGTTGGGTGC	70
71	ACGAGTGGGT	TACATCGAAC	TGGATCTCAA	CAGCGGTAAG	ATCCTTGAGA	GTTTTCGCCC	CGAAGAACGT	140
141	TTTCCAATGA	TGAGCACTTT	TAAAGTTCTG	CTATGTGGTG	CGGTATTATC	CCGTGTTGAC	GCCGGGCAAG	210
211	AGCAACTCGG	TCGCCGCATA	CACTATTCTC	AGAATGACTT	GGTTGAGTAC	TCACCAGTCA	CAGAAAAGCA	280
281	TCTTACGGAT	GGCATGACAG	TAAGAGAATT	ATGCAGTGCT	GCCATAACCA	TGAGTGATAA	CACTGCTGCC	350
351	AACTTACTTC	TGACAACGAT	CGGAGGACCG	AAGGAGCTAA	CCGCTTTTTT	GCACAACATG	GGGGATCATG	420
421	TAACTCGCCT	TGATCGTTGG	GAACCGGAGC	TGAATGAAGC	CATACCAAAC	GACGAGCGTG	ACACCACGAT	490
491	GCCTGCAGCA	ATGGCAACAA	CGTTGCGCAA	ACTATTAACT	GGCGAACTAC	TTACTCTAGC	TTCCCGGCAA	560
561	CAATTAATAG	ACTGGATGGA	GGCGGATAAA	GTTGCAGGAC	CACTTCTGCG	CTCGGCCCTT	CCGGCTGGGC	630
631	TGGTTTATTG	CTGATAAATC	TGGAGCCGGT	GAGCGTGGGG	T			671

Applied Biosystems

S/N G:3311 A:2149 T:1271 C:1577
KB.bcp
KB 1.4.0 Cap:1

121-F_3130POP7_v3.1_2012-10-21
121-F
KB_3130_POP7_BDTv3.mob
Pts 1967 to 8532 Pk1 Loc:1936
Version 5.3 HiSQV Bases: 653

Inst Model/Name 3100/3130GeneticAnalyzer-19348-006
Oct 21,2012 04:06PM, AST
Oct 21,2012 04:17PM, AST
Spacing:10.59 Pts/Panel1500
Plate Name: SS-21102012

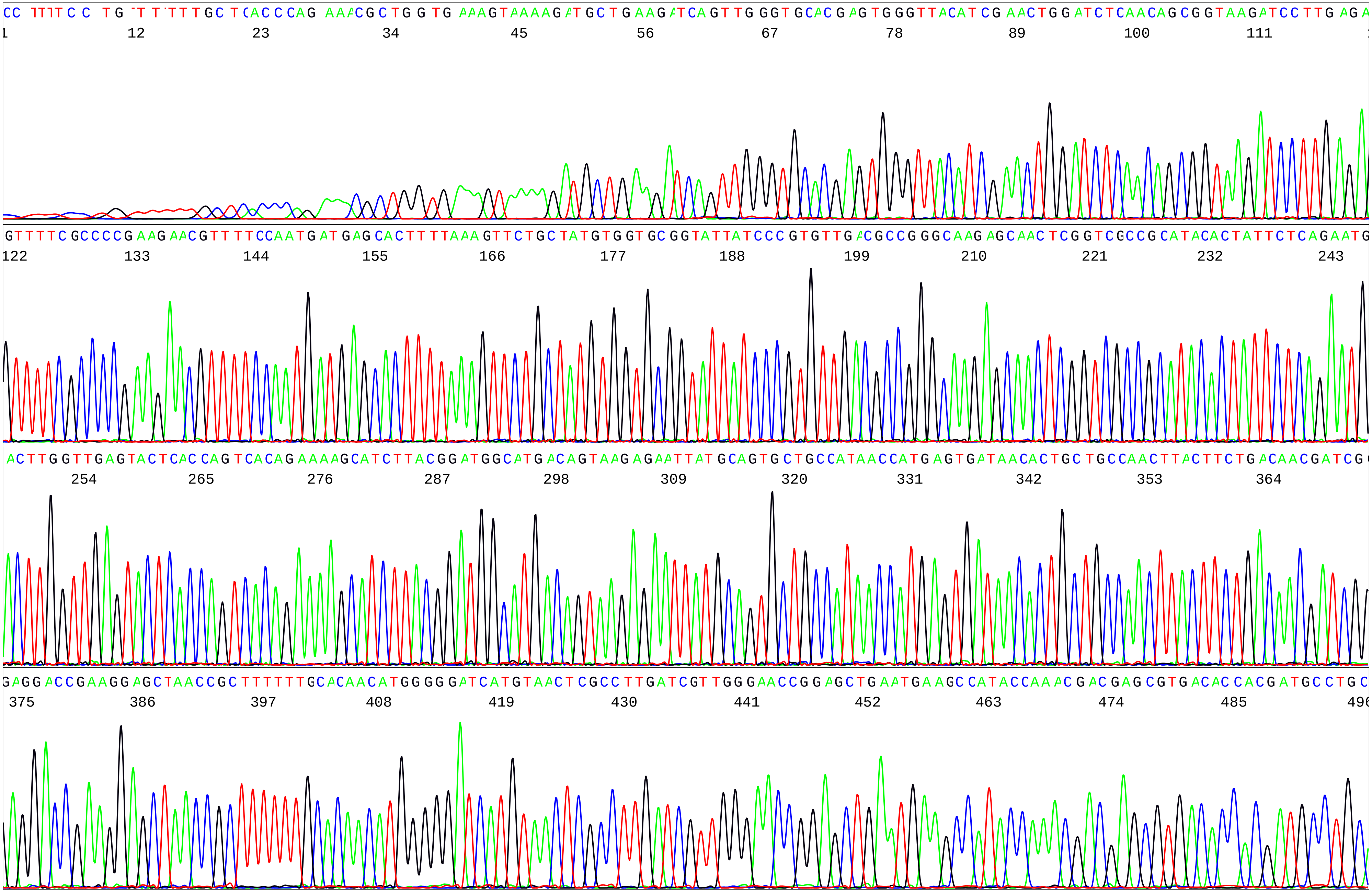

Printed on: Tue Oct 23,2012 02:20PM, AST

121-F_3130POP7_v3.1_2012-10-21
121-F
KB_3130_POP7_BDTv3.mob
Pts 1967 to 8532 Pk1 Loc:1936
Version 5.3   HiSQV Bases: 653

Inst Model/Name 3100/3130GeneticAnalyzer-19348-006
Oct 21,2012 04:06PM, AST
Oct 21,2012 04:17PM, AST
Spacing:10.59 Pts/Panel1500
Plate Name: SS-21102012

S/N G:3311 A:2149 T:1271 C:1577
KB.bcp
KB 1.4.0   Cap:1

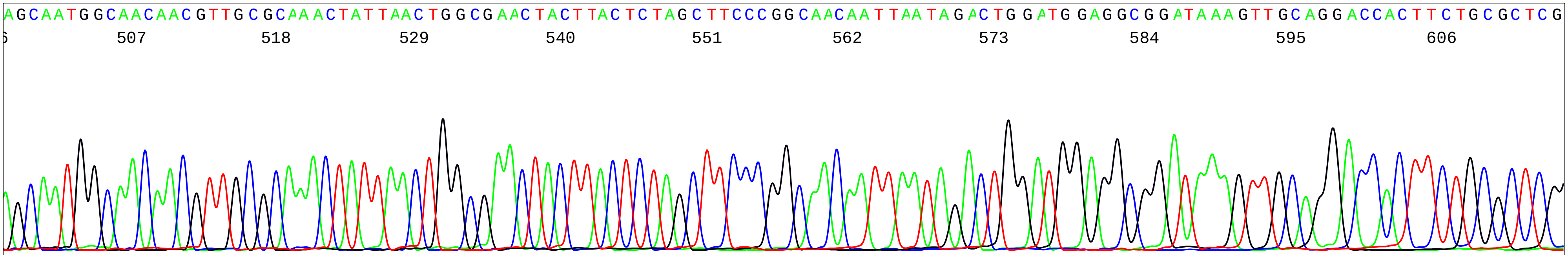

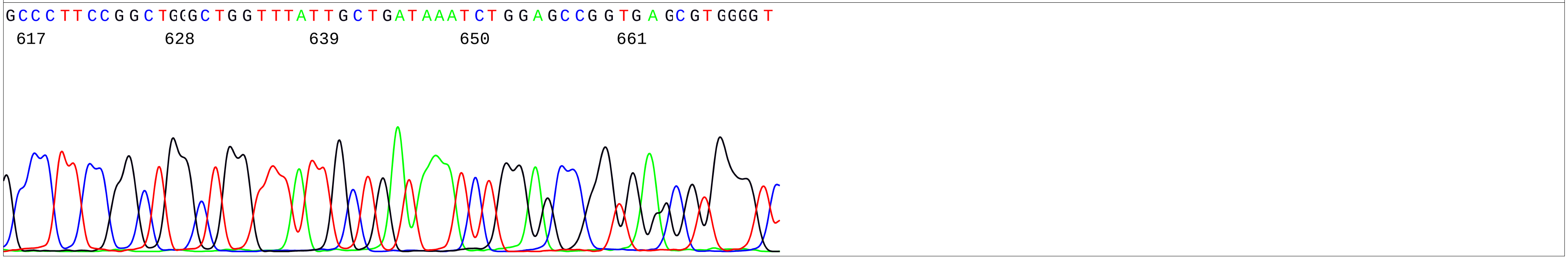

Supplement: Additional file 1: Table S1. — Specimen types and Demographics of E. coli O25b-B2-ST131 isolates. Samples from pus, skin and wound have been illustrated under soft tissue. [file 12866_2014_214_MOESM1_ESM.zip › 12866_2014_214_MOESM1_ESM/12866_2014_214_add2.pdf]
