# Supplementary material for: The characterization and antibiotic resistance profiles of clinical Escherichia coli O25b-B2-ST131 isolates in Kuwait
Source: BMC Microbiol. 2014 Aug 28;14:214. doi: 10.1186/s12866-014-0214-6 (PMC4159528; doi:10.1186/s12866-014-0214-6)

```
1    CTACCCGTAG CGGATGGACT GAACACTGGA ACGTCGCGGC CTAGTACCTA GCACCTCTTC CAACGGTTTC   70
71   CCCAGCTCAC ACTTTTCCAC ACGACTTTCG ATTATTGGCG TAGCTTAGAT CGGTATTCGT GTTATATGCG  140
141  CTACAAAACC ATGCGCGCGC GGCTTCATAT TCATAAAGCT CGCGCCGCGG AATTCTGCGC CTCGCGCGCG  210
211  GCAGCACAGA TTTCATTGCA AGCGCGCACA AGGCGCAGAT TTGCGCGGCG CAAGCTTTAT GAATATGATC  280
281  ACCACGCGCA CCTGGTTTTG TAGCGCATAT ATCACGAATA CCAATCTAAG CTACGCCAAT TTTTCGAAAG  350
351  TCGTGTTGGA AAAGTGTGAG CTGTGGGAAA ACCGTTGGAT AGGTGCCCAG GTACTGGGCG CGACGTTCAG  420
421  TGGTTCAGAT CTCTCCGGCG GCGAGTTTTC GACTTTCGAC TGGCGGAGCA GCAAAAATTG TTATTTTATA  490
491  AATTTTGGTG ACCCCAATAT CCGTTGGCAT CCCCGGGAGT CCGTGTGCTT CGCCGCCGCC CTCCCTCA    558
```

Applied Biosystems

S/N G:2837 A:2732 T:2112 C:2514
KB.bcp
KB 1.4.0 Cap:1

shurooq2_3130POP7_v3.1_2014-04-30
shurooq2
KB_3130_POP7_BDTv3.mob
Pts 1846 to 11071 Pk1 Loc:1823
Version 5.3 HiSQV Bases: 221

Inst Model/Name 3100/3130GeneticAnalyzer-19348-006
Apr 30,2014 01:36AM, AST
Apr 30,2014 01:58AM, AST
Spacing:-13.4 Pts/Panel1500
Plate Name: KhadijaMSC29-4-2014

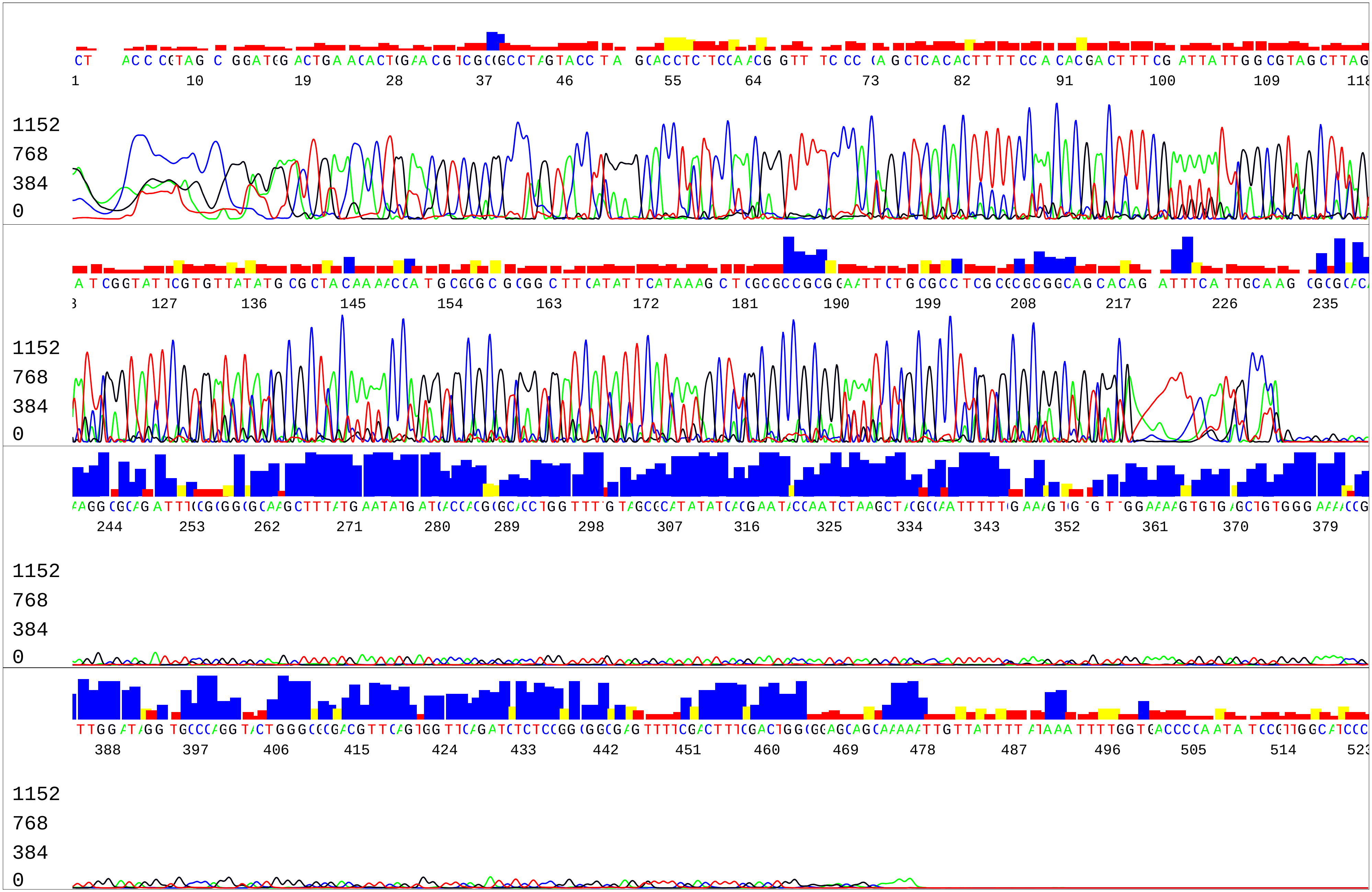

Applied Biosystems

S/N G:2837 A:2732 T:2112 C:2514	shurooq2_3130POP7_v3.1_2014-04-30	Inst Model/Name 3100/3130GeneticAnalyzer-19348-006
KB.bcp	shurooq2	Apr 30,2014 01:36AM, AST
KB 1.4.0 Cap:1	KB_3130_POP7_BDTv3.mob	Apr 30,2014 01:58AM, AST
	Pts 1846 to 11071 Pk1 Loc:1823	Spacing:-13.4 Pts/Panel1500
	Version 5.3 HiSQV Bases: 221	Plate Name: KhadijaMSC29-4-2014

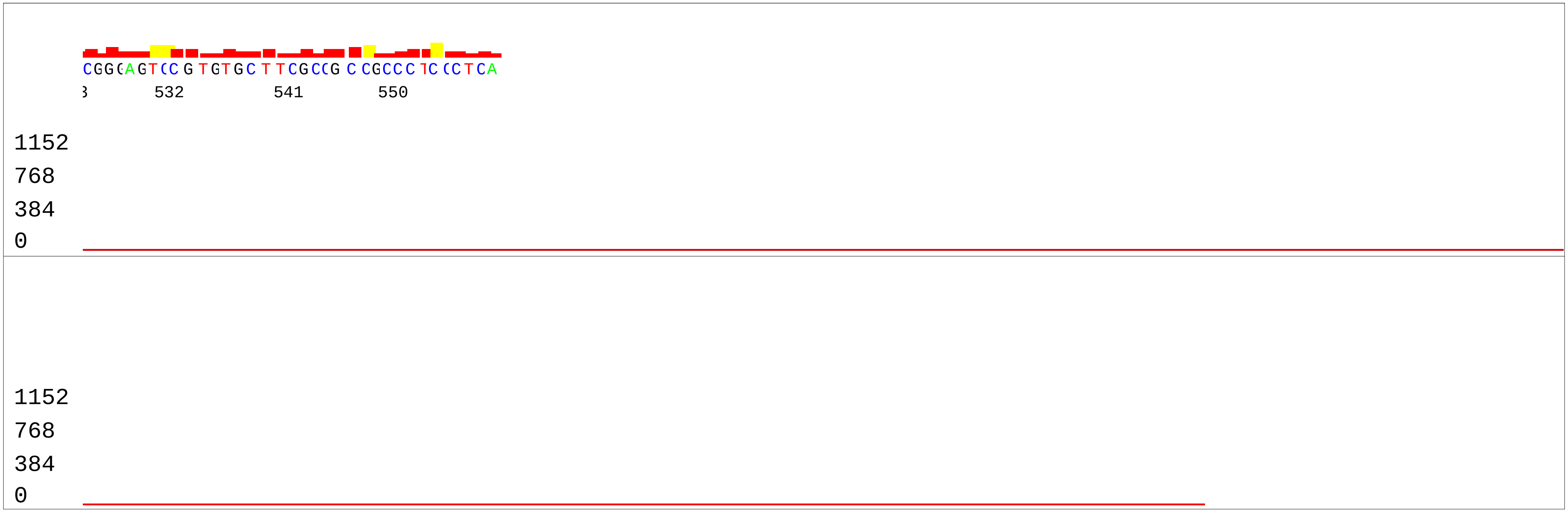

Supplement: Additional file 1: Table S1. — Specimen types and Demographics of E. coli O25b-B2-ST131 isolates. Samples from pus, skin and wound have been illustrated under soft tissue. [file 12866_2014_214_MOESM1_ESM.zip › 12866_2014_214_MOESM1_ESM/12866_2014_214_add20.pdf]
