# Supplementary material for: The characterization and antibiotic resistance profiles of clinical Escherichia coli O25b-B2-ST131 isolates in Kuwait
Source: BMC Microbiol. 2014 Aug 28;14:214. doi: 10.1186/s12866-014-0214-6 (PMC4159528; doi:10.1186/s12866-014-0214-6)

Applied Biosystems

QNRB4_3130POP7_v3.1_2012-09-18
QNRB4
KB_3130_POP7_BDTv3.mob
Pts 2362 to 8532 Pk1 Loc:2331
Version 5.3 HiSQV Bases: 171

Inst Model/Name 3100/3130GeneticAnalyzer-19348-006
Sep 18,2012 09:33AM, AST
Sep 18,2012 09:55AM, AST
Spacing:11.03
Plate Name: 18092012

S/N G:410 A:145 T:151 C:215
KB.bcp
KB 1.4.0 Cap:2

```
  1   GGGGTTTTTT GTTAAGCGCA TATATCACGA ATACCAATCT AAGCTACGCC AATTTTTCGA AAGTCGTGTT    70
 71   GGAAAAGTGT GAGCTGTGGG AAAACCGTTG GATAGGTGCC CAGGTACTGG GCGCGACGTT CAGTGGTTCA   140
141   GATCTCTCCG GCGGCGAGTT TTCGACTTTC GACTGGCGGG CGGCAAA                            187
```

Applied Biosystems	QNRB4_3130POP7_v3.1_2012-09-18	Inst Model/Name 3100/3130GeneticAnalyzer-19348-006
S/N G:410 A:145 T:151 C:215	QNRB4	Sep 18,2012 09:33AM, AST
KB.bcp	KB_3130_POP7_BDTv3.mob	Sep 18,2012 09:55AM, AST
KB 1.4.0 Cap:2	Pts 2362 to 8532 Pk1 Loc:2331	Spacing:11.03 Pts/Panel1500
	Version 5.3 HiSQV Bases: 171	Plate Name: 18092012

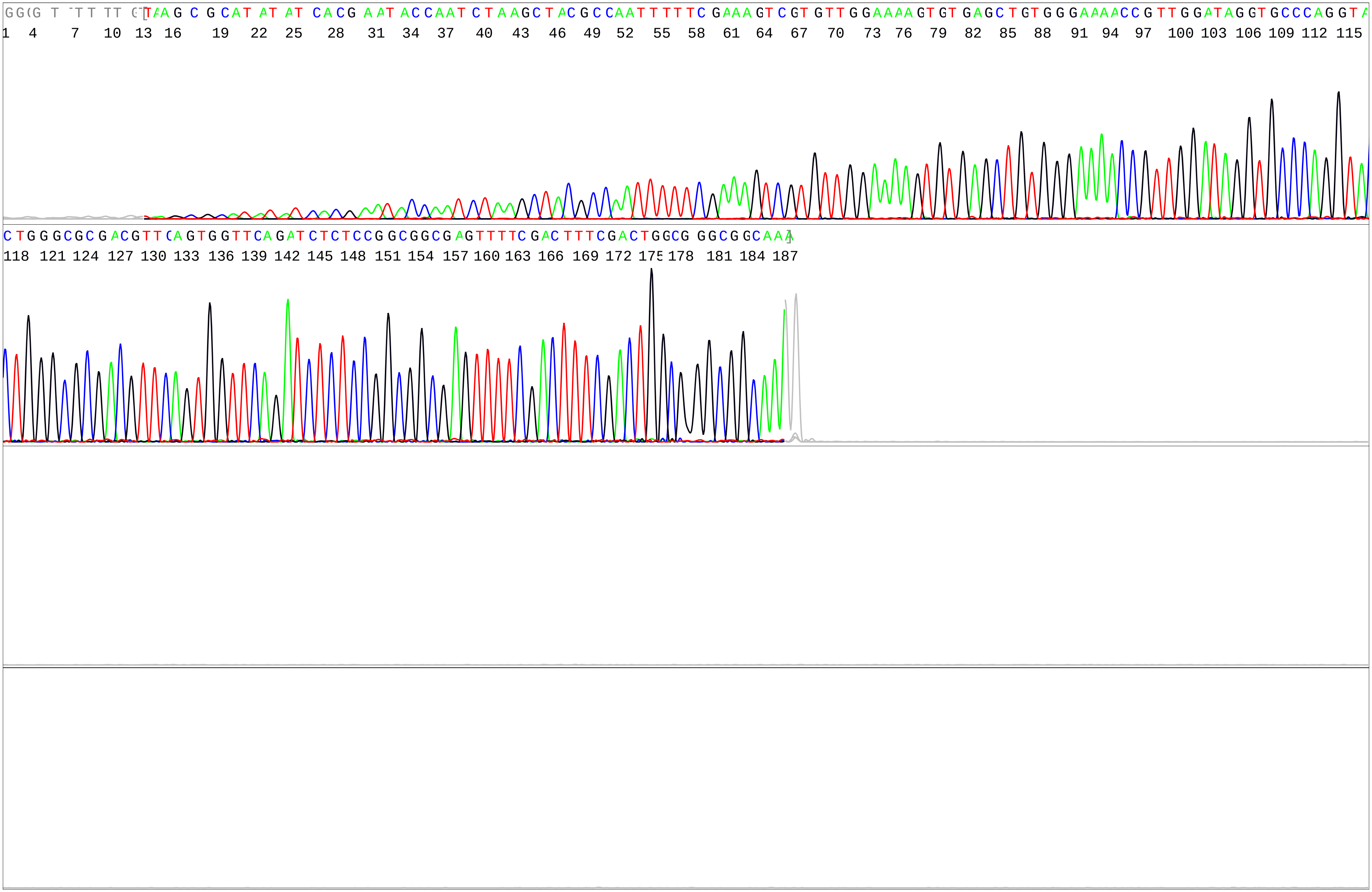

Applied Biosystems

	QNRB4_3130POP7_v3.1_2012-09-18	Inst Model/Name 3100/3130GeneticAnalyzer-19348-006
	QNRB4	Sep 18,2012 09:33AM, AST
S/N G:410 A:145 T:151 C:215	KB_3130_POP7_BDTv3.mob	Sep 18,2012 09:55AM, AST
KB.bcp	Pts 2362 to 8532 Pk1 Loc:2331	Spacing:11.03 Pts/Panel1500
KB 1.4.0 Cap:2	Version 5.3 HiSQV Bases: 171	Plate Name: 18092012

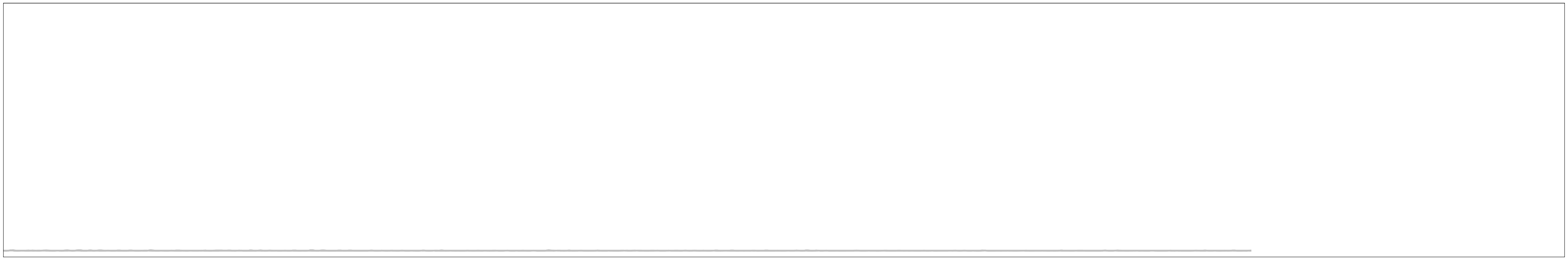

Supplement: Additional file 1: Table S1. — Specimen types and Demographics of E. coli O25b-B2-ST131 isolates. Samples from pus, skin and wound have been illustrated under soft tissue. [file 12866_2014_214_MOESM1_ESM.zip › 12866_2014_214_MOESM1_ESM/12866_2014_214_add21.pdf]
