# Supplementary material for: The characterization and antibiotic resistance profiles of clinical Escherichia coli O25b-B2-ST131 isolates in Kuwait
Source: BMC Microbiol. 2014 Aug 28;14:214. doi: 10.1186/s12866-014-0214-6 (PMC4159528; doi:10.1186/s12866-014-0214-6)

S/N G:2127 A:1682 T:1394 C:1180
KB.bcp
KB 1.4.0 Cap:2

shurooq3_3130POP7_v3.1_2014-04-30
shurooq3
KB_3130_POP7_BDTv3.mob
Pts 1880 to 11071 Pk1 Loc:1857
Version 5.3 HiSQV Bases: 227

Inst Model/Name 3100/3130GeneticAnalyzer-19348-006
Apr 30,2014 01:36AM, AST
Apr 30,2014 01:58AM, AST
Spacing:-13.4
Plate Name: KhadijaMSC29-4-2014

```
  1  CGTCAATTCG CGGCGCAAGC TTTATGAATA TGATCACCAC GCGCACCTGG TTTTGTAGCG CATATATCAC   70
 71  GAATACCAAT CTAAGCTACG CCAATTTTTC GAATGTCGTG TTGGAAATGT GTGAGCTCGT GGGATAACCG  140
141  TTGGATAGGT GCCCAGGTAC CGGGCGCGAC GTTCAGTGGT TCAGATCTCT CCGGCGGCGA GTTTTCGACT  210
211  TTCGACCGGG AACGGCAAAA CAGCCAGATT CATGGGCGCA AGCTTTATGA ATAATGATCA CCACCACGCA  280
281  CCCTGGTTTT GTAGCGCATA TATTCGAATA TAACCTAAGC TAGCAATTTT TTTTTTTGAA ATGGAAAATT  350
351  GTGAATGTGG GAAAACGTTG GAAACCTGTC GGTACTGGGC CGAGTTCAGG GGTTACATTC TCCGGTGCAA  420
421  AGTTTCTACT TTCGACTGGG GAACAGCAAA AGATGTTGTT TTATGGGAAG GGGGGGGGGG GGCGCGCCCA  490
491  CCCGGAGGAG GAAGGGAAGA GGGTGGGTCC GTTGCGCTCT ATCAGCTTAG TTATGGGGGG GGGCGGGAGG  560
561  GGGGAGGGGT AGAGTGCGTC GGGCAAAGGA ACGTCAGGAG CTCGTACAAT GATAATATGC CCCGTCGCGT  630
631  GC                                                                           632
```

Applied Biosystems

shurooq3_3130POP7_v3.1_2014-04-30
shurooq3
KB_3130_POP7_BDTv3.mob
Pts 1880 to 11071 Pk1 Loc:1857
Version 5.3 HiSQV Bases: 227

Inst Model/Name 3100/3130GeneticAnalyzer-19348-006
Apr 30,2014 01:36AM, AST
Apr 30,2014 01:58AM, AST
Spacing:-13.4 Pts/Panel1500
Plate Name: KhadijaMSC29-4-2014

S/N G:2127 A:1682 T:1394 C:1180
KB.bcp
KB 1.4.0 Cap:2

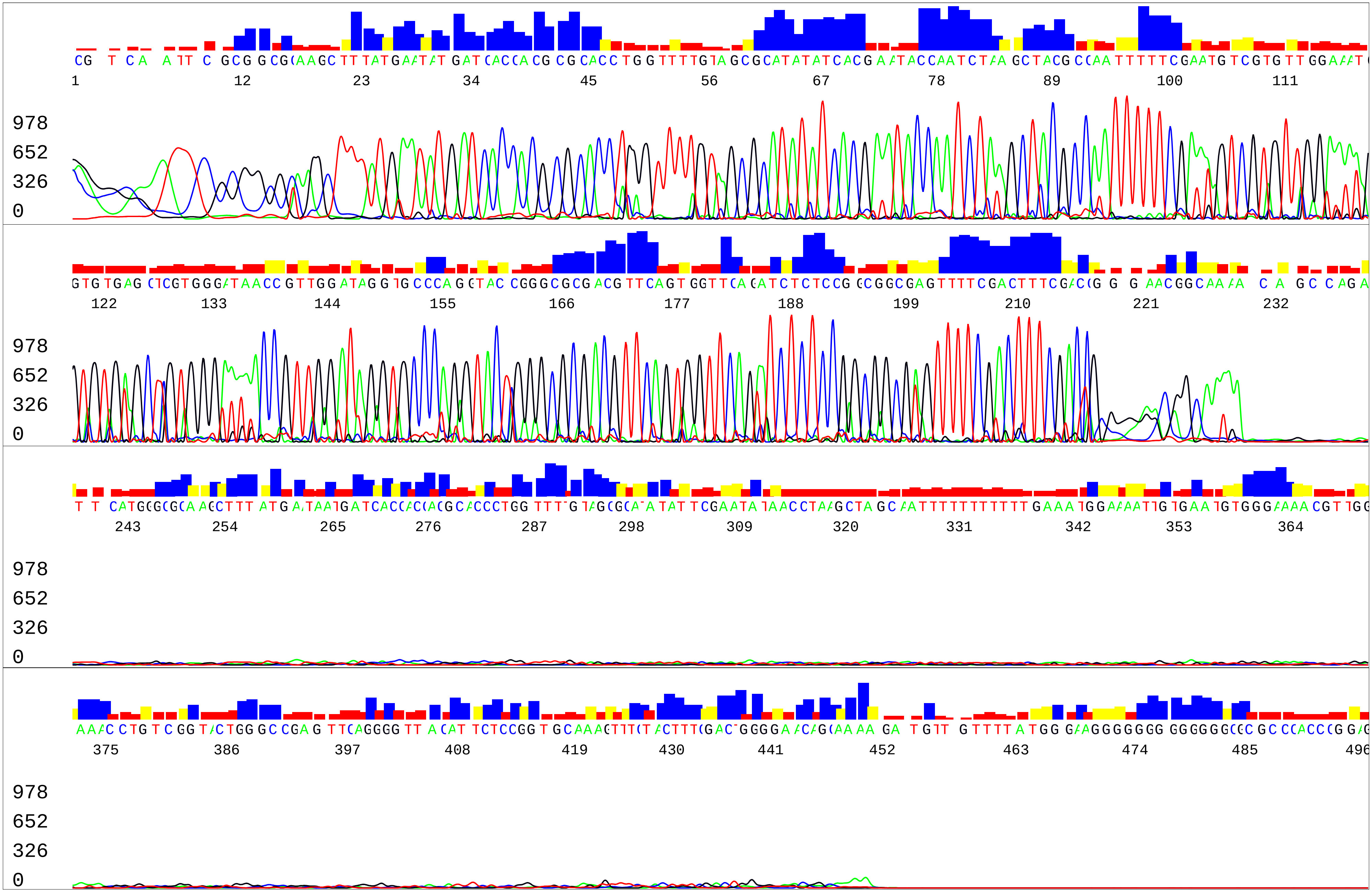

S/N G:2127 A:1682 T:1394 C:1180
KB.bcp
KB 1.4.0 Cap:2

shurooq3_3130POP7_v3.1_2014-04-30
shurooq3
KB_3130_POP7_BDTv3.mob
Pts 1880 to 11071 Pk1 Loc:1857
Version 5.3 HiSQV Bases: 227

Inst Model/Name 3100/3130GeneticAnalyzer-19348-006
Apr 30,2014 01:36AM, AST
Apr 30,2014 01:58AM, AST
Spacing:-13.4 Pts/Panel1500
Plate Name: KhadijaMSC29-4-2014

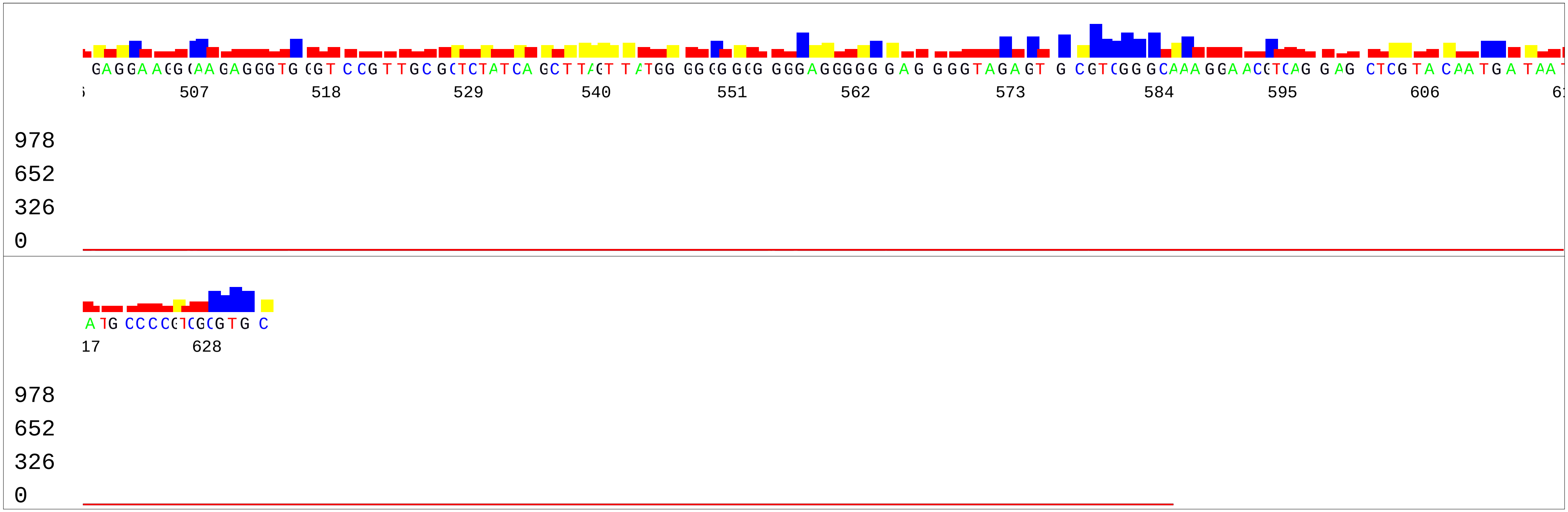

Supplement: Additional file 1: Table S1. — Specimen types and Demographics of E. coli O25b-B2-ST131 isolates. Samples from pus, skin and wound have been illustrated under soft tissue. [file 12866_2014_214_MOESM1_ESM.zip › 12866_2014_214_MOESM1_ESM/12866_2014_214_add22.pdf]
