# Supplementary material for: The characterization and antibiotic resistance profiles of clinical Escherichia coli O25b-B2-ST131 isolates in Kuwait
Source: BMC Microbiol. 2014 Aug 28;14:214. doi: 10.1186/s12866-014-0214-6 (PMC4159528; doi:10.1186/s12866-014-0214-6)

S/N G:2417 A:1305 T:1064 C:1061
KB.bcp
KB 1.4.0 Cap:3

shurooq4_3130POP7_v3.1_2014-04-30
shurooq4
KB_3130_POP7_BDTv3.mob
Pts 1883 to 11071 Pk1 Loc:1860
Version 5.3 HiSQV Bases: 665

Inst Model/Name 3100/3130GeneticAnalyzer-19348-006
Apr 30,2014 01:36AM, AST
Apr 30,2014 01:58AM, AST
Spacing:13.35
Plate Name: KhadijaMSC29-4-2014

1	GTTCCGGAGA	CTCACGCACT	GCACGGTTCG	AATGTCGTAA	CCGCTGCGGA	GCAAGGCCGT	CGCGAACGAG	70
71	TGGCGGAGGG	TGTGCGGTGT	GGCGGGCTTC	GTGATGCCTG	CTTGTTCTAC	GGCACGTTTG	AAGGCGCGCT	140
141	GAAAGGTCTG	GTCATACATG	TGATGGCGAC	GCACGACACC	GCTCCGTGGA	TCGGTCGAAT	GCGTGTGCTG	210
211	CGCAAAAACC	CAGAACCACG	GCCAGGAATG	CCCGGCGCGC	GGATACTTCC	GCTCAAGGGC	GTCGGGAAGC	280
281	GCAACGCCGC	TGCGGCCCTC	GGCCTGGTCC	TTCAGCCACC	ATGCCCGTGC	ACGCGACAGC	TGCTCGCGCA	350
351	GGCTGGGTGC	CAAGCTCTCG	GGTAACATCA	AGGCCCGATC	CTTGGAGCCC	TTGCCCTCCC	GCACGATGAT	420
421	CGTGCCGTGA	TCGAAATCCA	GATCCTTGAC	CCGCAGTTGC	AAACCCTCAC	TGATCCGCAT	GCCCGTTCCA	490
491	TACAGAAGCT	GGGCGAACAA	ACGATGCTCG	CCTTCCAGAA	AACCGAGGAT	GCGAACCACT	TCATCCGGGG	560
561	TCAGCACCAC	CGGCAAGCGC	CGCGACGGCC	GAGGTCTTCC	GATCTCCTGA	AGCCAGGGCA	GATCCGTGCA	630
631	CAGCACCTTG	CCGTAGAAGA	ACAGCAAGGG	CCGCCAATGC	CTGACGATGC	GTGGAGACCG	AAACCTTGCG	700
701	CTCGTTCGCC	AGCCAGGACA	GA					722

Applied Biosystems

shurooq4_3130POP7_v3.1_2014-04-30
shurooq4
KB_3130_POP7_BDTv3.mob
Pts 1883 to 11071 Pk1 Loc:1860
Version 5.3   HiSQV Bases: 665

Inst Model/Name 3100/3130GeneticAnalyzer-19348-006
Apr 30,2014 01:36AM, AST
Apr 30,2014 01:58AM, AST
Spacing:13.35 Pts/Panel1500
Plate Name: KhadijaMSC29-4-2014

S/N G:2417 A:1305 T:1064 C:1061
KB.bcp
KB 1.4.0   Cap:3

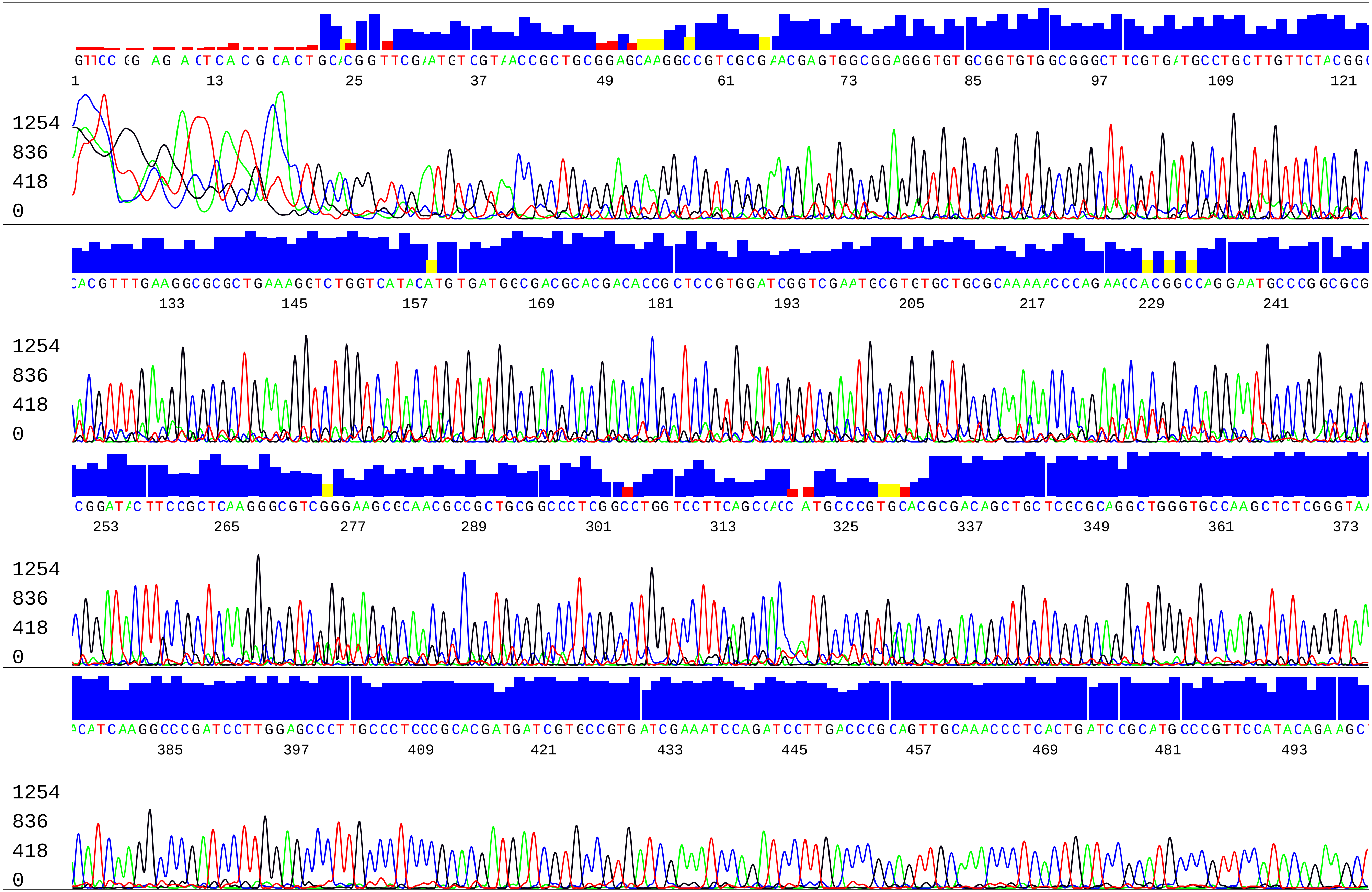

	shurooq4_3130POP7_v3.1_2014-04-30	Inst Model/Name 3100/3130GeneticAnalyzer-19348-006
	shurooq4	Apr 30,2014 01:36AM, AST
S/N G:2417 A:1305 T:1064 C:1061	KB_3130_POP7_BDTv3.mob	Apr 30,2014 01:58AM, AST
KB.bcp	Pts 1883 to 11071 Pk1 Loc:1860	Spacing:13.35 Pts/Panel1500
KB 1.4.0 Cap:3	Version 5.3 HiSQV Bases: 665	Plate Name: KhadijaMSC29-4-2014

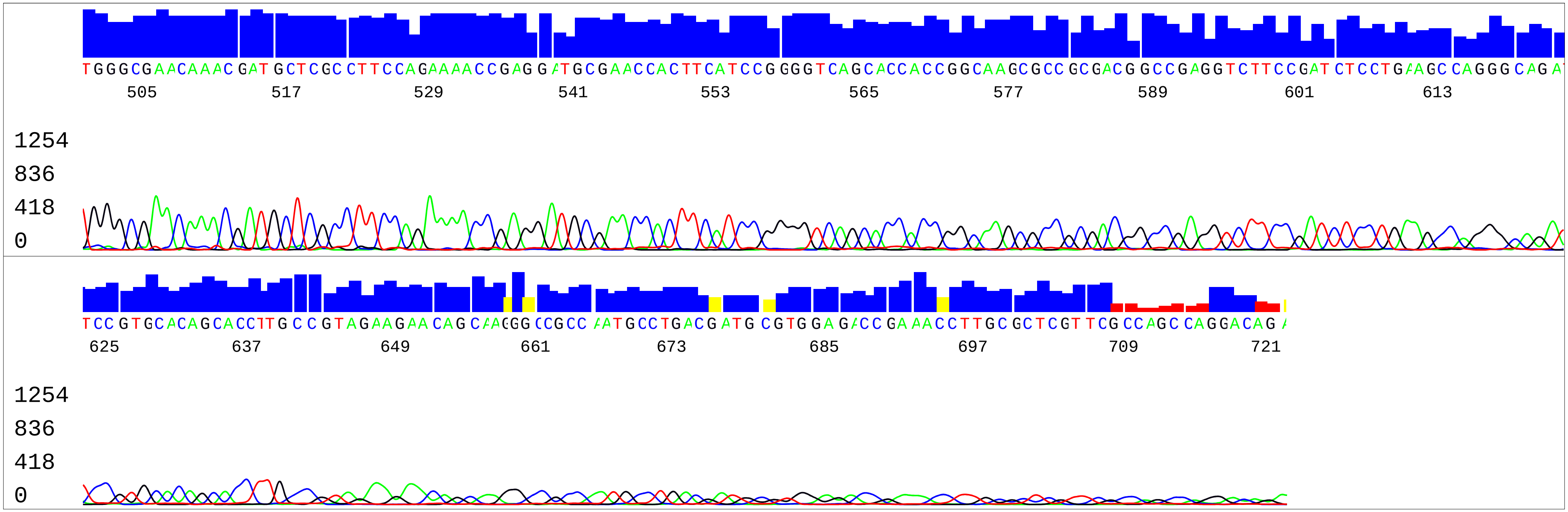

Supplement: Additional file 1: Table S1. — Specimen types and Demographics of E. coli O25b-B2-ST131 isolates. Samples from pus, skin and wound have been illustrated under soft tissue. [file 12866_2014_214_MOESM1_ESM.zip › 12866_2014_214_MOESM1_ESM/12866_2014_214_add23.pdf]
