# Supplementary material for: The characterization and antibiotic resistance profiles of clinical Escherichia coli O25b-B2-ST131 isolates in Kuwait
Source: BMC Microbiol. 2014 Aug 28;14:214. doi: 10.1186/s12866-014-0214-6 (PMC4159528; doi:10.1186/s12866-014-0214-6)

Applied Biosystems

	7_3130POP7_v3.1_2013-01-08	Inst Model/Name 3100/3130GeneticAnalyzer-19348-006
	7	Jan 08,2013 09:00AM, AST
S/N G:490 A:250 T:244 C:478	KB_3130_POP7_BDTv3.mob	Jan 08,2013 09:11AM, AST
KB.bcp	Pts 2242 to 8532 Pk1 Loc:2211	Spacing:11.31
KB 1.4.0 Cap:3	Version 5.3 HiSQV Bases: 569	Plate Name: manar07012013

```
  1  TGCCTTTTTG GAGGCCAAAT TTAAACAAAG CGAAAGCCAG CTGTCGGGCC GCGTAGGCAT GATAGAAATG   70
 71  GATCTGGCCA GCGGCCGCAC GCTGACCGCC TGGCGCGCCG ATGAACGCTT TCCCATGATG AGCACCTTTA  140
141  AAGTAGTGCT CTGCGGCGCA GTGCTGGCGC GGGTGGATGC CGGTGACGAA CAGCTGGAGC GAAAGATCCA  210
211  CTATCGCCAG CAGGATCTGG TGGACTACTC GCCGGTCAGC GAAAAACACC TTGCCGACGG CATGACGGTC  280
281  GGCGAACTCT GCGCCGCCGC CATTACCATG AGCGATAACA GCGCCGCCAA TCTGCTGCTG GCCACCGTCG  350
351  GCGGCCCCGC AGGATTGACT GCCTTTTTGC GCCAGATCGG CGACAACGTC ACCCGCCTTG ACCGCTGGGA  420
421  AACGGAACTG AATGAGGCGC TTCCCGGCGA CGCCCGCGAC ACCACTACCC CGGCCAGCAT GGCCGCGACC  490
491  CTGCGCAAGC TGCTGACCAG CCAGCGTCTG AGCGCCCGTT CGCAACGGCA GCTGCTGCAG TGGATGGTGG  560
561  ACGATCGGGT CGCCGGACCG TTGATCCGCT CCGTGC                                       596
```

Applied Biosystems

7_3130POP7_v3.1_2013-01-08
7
KB_3130_POP7_BDTv3.mob
Pts 2242 to 8532 Pk1 Loc:2211
Version 5.3 HiSQV Bases: 569

Inst Model/Name 3100/3130GeneticAnalyzer-19348-006
Jan 08,2013 09:00AM, AST
Jan 08,2013 09:11AM, AST
Spacing:11.31 Pts/Panel1500
Plate Name: manar07012013

S/N G:490 A:250 T:244 C:478
KB.bcp
KB 1.4.0 Cap:3

TGCCTTTGGAGGCAAATTTAAACAAAGCGAAAGCCAGCTGTCGGGCCGCGTAGGCATGATAGAAATGGATCTGGCCAGCGGCCGCACGCTGACCGCCTGGCGCGCCGATGAACGCTTTCCC

1 12 23 34 45 56 67 78 89 100 111 122

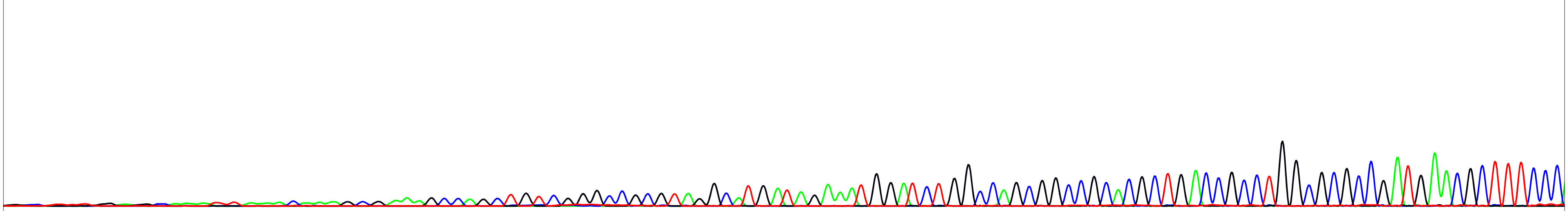

ATGATGAGCACCTTTAAAGTAGTGCTCTGCGGCGCAGTGCTGGCGCGGGTGGATGCCGGTGACGAACAGCTGGAGCGAAAGATCCACTATCGCCAGCAGGATCTGGTGGACTACTCGCCGGTCAGC

133 144 155 166 177 188 199 210 221 232 243

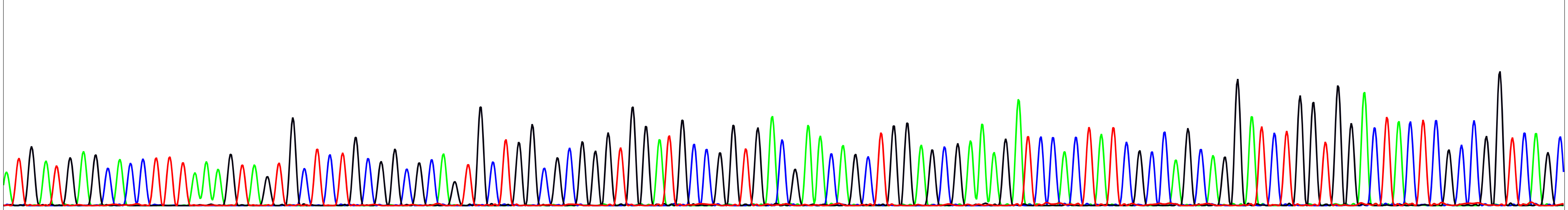

GAAAAACACCTTGCCGACGGCATGACGGTCGGCGAACTCTGCGCCGCCGCCATTACCATGAGCGATAACAGCGCCGCCAATCTGCTGCTGGCCACCGTCGGCGGCCCCGCAGGATTGACTGCCTTT

254 265 276 287 298 309 320 331 342 353 364 375

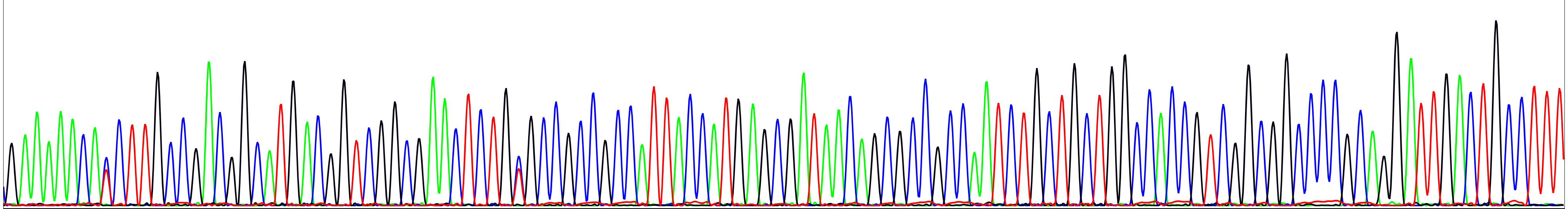

TTGCGCCAGATCGGCGACAACGTCACCCGCCTTGACCGCTGGGAAACGGAACTGAATGAGGCGCTTCCCGGCGACGCCCGCGACACCACTACCCCGGCCAGCATGGCCGCGACCCTGCGCAAGC

386 397 408 419 430 441 452 463 474 485 496

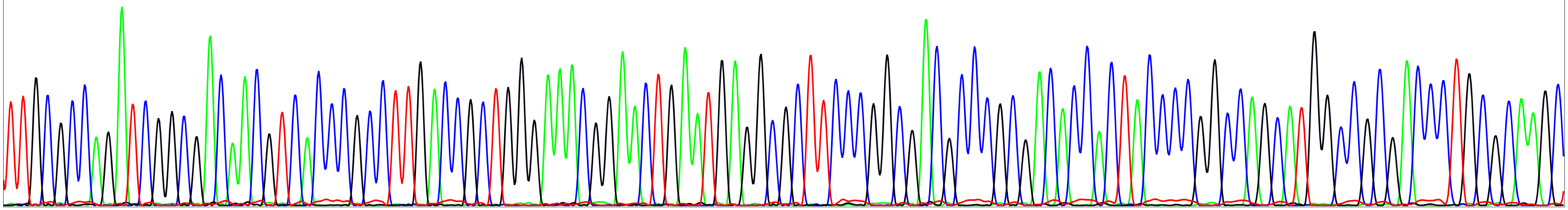

Applied Biosystems

7_3130POP7_v3.1_2013-01-08
7
KB_3130_POP7_BDTv3.mob
Pts 2242 to 8532 Pk1 Loc:2211
Version 5.3   HiSQV Bases: 569

Inst Model/Name 3100/3130GeneticAnalyzer-19348-006
Jan 08,2013 09:00AM, AST
Jan 08,2013 09:11AM, AST
Spacing:11.31 Pts/Panel1500
Plate Name: manar07012013

S/N G:490 A:250 T:244 C:478
KB.bcp
KB 1.4.0   Cap:3

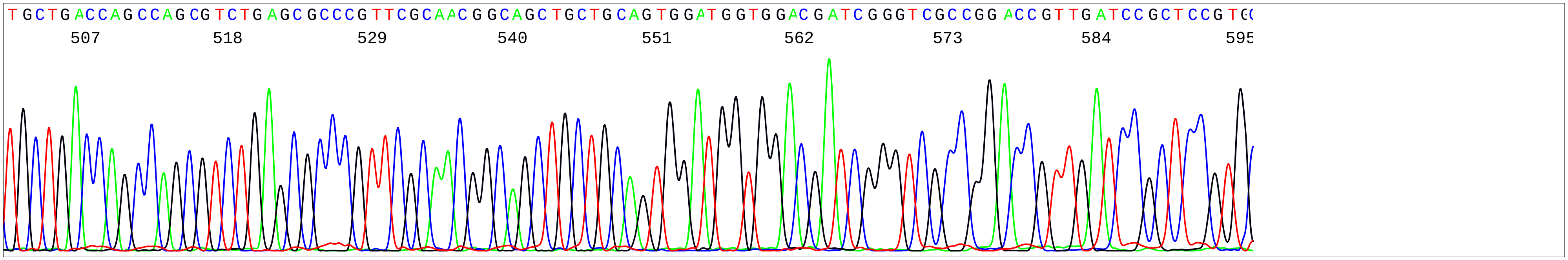

Supplement: Additional file 1: Table S1. — Specimen types and Demographics of E. coli O25b-B2-ST131 isolates. Samples from pus, skin and wound have been illustrated under soft tissue. [file 12866_2014_214_MOESM1_ESM.zip › 12866_2014_214_MOESM1_ESM/12866_2014_214_add24.pdf]
