# Supplementary material for: The characterization and antibiotic resistance profiles of clinical Escherichia coli O25b-B2-ST131 isolates in Kuwait
Source: BMC Microbiol. 2014 Aug 28;14:214. doi: 10.1186/s12866-014-0214-6 (PMC4159528; doi:10.1186/s12866-014-0214-6)

Applied Biosystems

	8_3130POP7_v3.1_2013-01-08	Inst Model/Name 3100/3130GeneticAnalyzer-19348-006
	8	Jan 08,2013 09:00AM, AST
S/N G:442 A:190 T:218 C:300	KB_3130_POP7_BDTv3.mob	Jan 08,2013 09:11AM, AST
KB.bcp	Pts 2243 to 8532 Pk1 Loc:2212	Spacing:11.38
KB 1.4.0 Cap:4	Version 5.3 HiSQV Bases: 568	Plate Name: manar07012013

```
  1   CGGCGGTAAT CCCGCAAGAT AAATCACCAC AATGCGCTCT GCTTTGTTAT TCGGGCCAAG CAGGGCGACA    70
 71   ATCCCGCGCG CACCCCGCTC GCCAGCTCCG GTCTTATCGG CGATAAACCA GCCCGCCGGC AGCACGGAGC   140
141   GGATCAACGG TCCGGCGACC CGATCGTCCA CCATCCACTG CAGCAGCTGC CGTTGCGAAC GGGCGCTCAG   210
211   ACGCTGGCTG GTCAGCAGCT TGCGCAGGGT CGCGGCCATG CTGGCCGGGG TAGTGGTGTC GCGGGCGTCG   280
281   CCGGGAAGCG CCTCATTCAG TTCCGTTTCC CAGCGGTCAA GGCGGGTGAC GTTGTCGCCG ATCTGGCGCA   350
351   AAAAGGCAGT CAATCCTGCG GGGCCGCCGA CGGTGGCCAG CAGCAGATTG GCGGCGCTGT TATCGCTCAT   420
421   GGTAATGGCG GCGGCGCAGA GTTCGCCGAC CGTCATGCCG TCGGCAAGGT GTTTTTCGCT GACCGGCGAG   490
491   TAGTCCACCA GATCCTGCTG GCGATAGTGG ATCTTTCGCT CCAGCTGTTC GTCACCGGCA TCCACCCGCG   560
561   CCAGCACTGC GCCGCAGAGC ACTACT                                                   586
```

Applied Biosystems

S/N G:442 A:190 T:218 C:300
KB.bcp
KB 1.4.0 Cap:4

8_3130POP7_v3.1_2013-01-08
8
KB_3130_POP7_BDTv3.mob
Pts 2243 to 8532 Pk1 Loc:2212
Version 5.3 HiSQV Bases: 568

Inst Model/Name 3100/3130GeneticAnalyzer-19348-006
Jan 08,2013 09:00AM, AST
Jan 08,2013 09:11AM, AST
Spacing:11.38 Pts/Panel1500
Plate Name: manar07012013

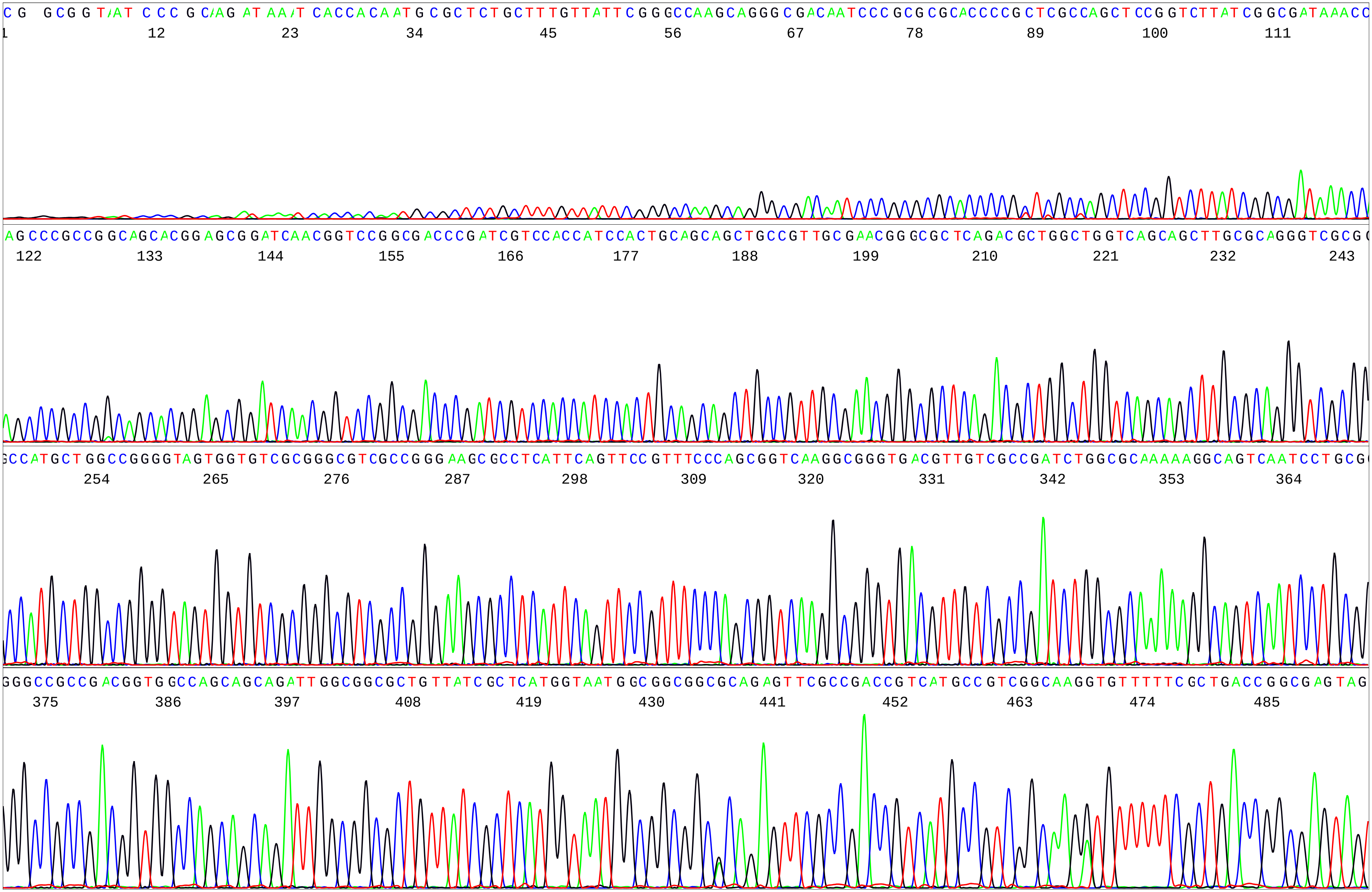

Applied Biosystems

S/N G:442 A:190 T:218 C:300	8_3130POP7_v3.1_2013-01-08	Inst Model/Name 3100/3130GeneticAnalyzer-19348-006
	8	Jan 08,2013 09:00AM, AST
KB.bcp	KB_3130_POP7_BDTv3.mob	Jan 08,2013 09:11AM, AST
KB 1.4.0 Cap:4	Pts 2243 to 8532 Pk1 Loc:2212	Spacing:11.38 Pts/Panel1500
	Version 5.3 HiSQV Bases: 568	Plate Name: manar07012013

TCCACCAGATCCTGCTGGCGATAGTGGATCTTTCGCTCCAGCTGTTCGTCACCGGCATCCACCCGCGCCAGCACTGCGCCGCAGAGCACTACT

496 507 518 529 540 551 562 573 584

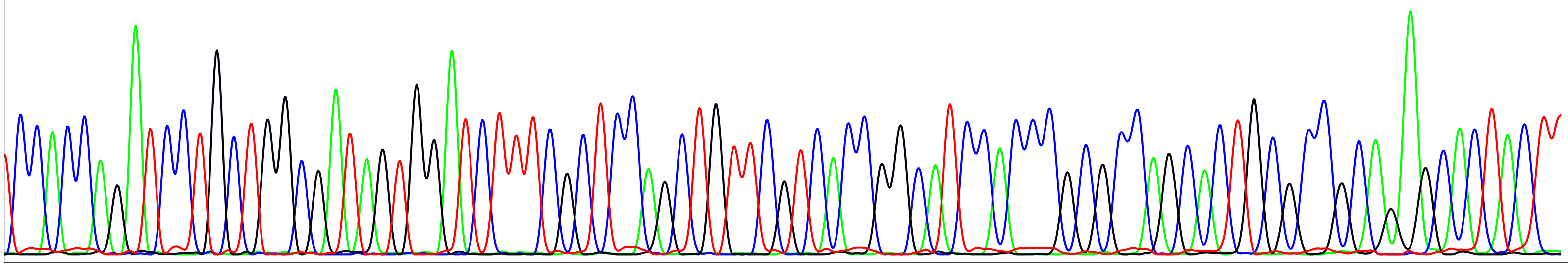

Supplement: Additional file 1: Table S1. — Specimen types and Demographics of E. coli O25b-B2-ST131 isolates. Samples from pus, skin and wound have been illustrated under soft tissue. [file 12866_2014_214_MOESM1_ESM.zip › 12866_2014_214_MOESM1_ESM/12866_2014_214_add25.pdf]
