# Supplementary material for: The characterization and antibiotic resistance profiles of clinical Escherichia coli O25b-B2-ST131 isolates in Kuwait
Source: BMC Microbiol. 2014 Aug 28;14:214. doi: 10.1186/s12866-014-0214-6 (PMC4159528; doi:10.1186/s12866-014-0214-6)

Applied Biosystems

S/N G:402 A:148 T:259 C:326
KB.bcp
KB 1.4.0 Cap:3

Dr.Suleiman_2011-03-13_TetAF_C11
TetAF
KB_3130_POP7_BDTv3.mob
Pts 2771 to 8532 Pk1 Loc:2740
Version 5.3 HiSQV Bases: 503

Inst Model/Name 3100/3130RCF-19348-006
Mar 13,2011 04:52PM, GMT+03:00
Mar 13,2011 05:03PM, GMT+03:00
Spacing:11.37
Plate Name: Dr.Suleiman

```
  1  GAAAACGGAC GTCACCGCCC ACTATGGCAT TCTGCTGGCG CTGTATGCGT TGATGCAATT TGCCTGCGCA CCTGTGCTGG   80
 81  GCGCGCTGTC GGATCGTTTC GGGCGGCGGC CGGTCTTGCT CGTCTCGCTG GCCGGCGCTG CTGTCGACTA CGCCATCATG  160
161  GCGACGGCGC CTTTCCTTTG GGTTCTCTAT ATCGGGCGGA TCGTGGCCGG CATCACCGGG GCGACTGGGG CGGTAGCCGG  240
241  CGCTTATATT GCCGATATCA CTGATGGCGA TGAGCGCGCG CGGCACTTCG GCTTCATGAG CGCCTGTTTC GGGTTCGGGA  320
321  TGGTCGCGGG ACCTGTGCTC GGTGGGCTGA TGGGCGGTTT CTCCCCCCAC GCTCCGTTCT TCGCCGCGGC AGCCTTGAAC  400
401  GGCCTCAATT TCCTGACGGG CTGTTTCCTT TTGCCGGAGT CGCACAAAGG CGAACGCCGG CCGTTACGCC GGGAGGCTCT  480
481  CAACCCGCTC GCTTCGTTCC GGTGGGGCCC GGGGGCATGA CC                                          522
```

Applied Biosystems

Dr.Suleiman_2011-03-13_TetAF_C11
TetAF
KB_3130_POP7_BDTv3.mob
Pts 2771 to 8532 Pk1 Loc:2740
Version 5.3   HiSQV Bases: 503

Inst Model/Name 3100/3130RCF-19348-006
Mar 13,2011 04:52PM, GMT+03:00
Mar 13,2011 05:03PM, GMT+03:00
Spacing:11.37 Pts/Panel1000
Plate Name: Dr.Suleiman

S/N G:402 A:148 T:259 C:326
KB.bcp
KB 1.4.0   Cap:3

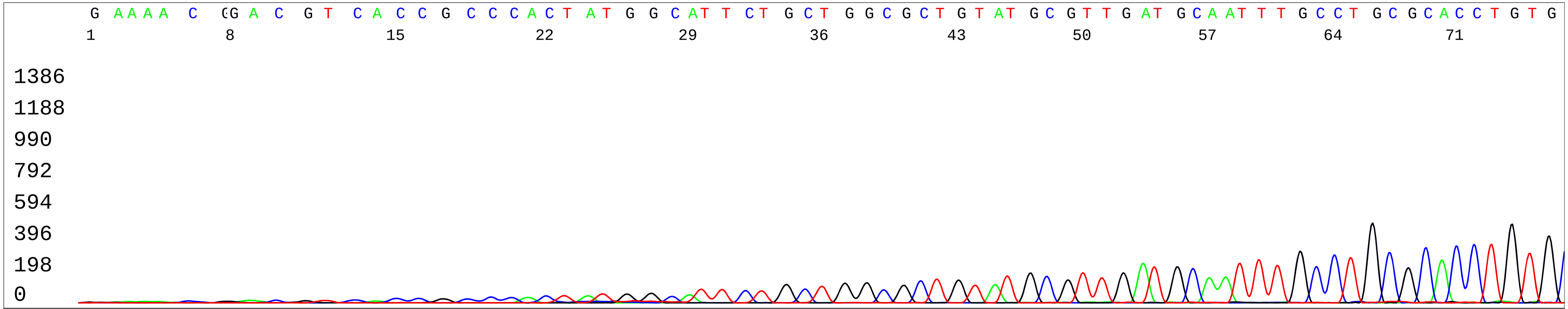

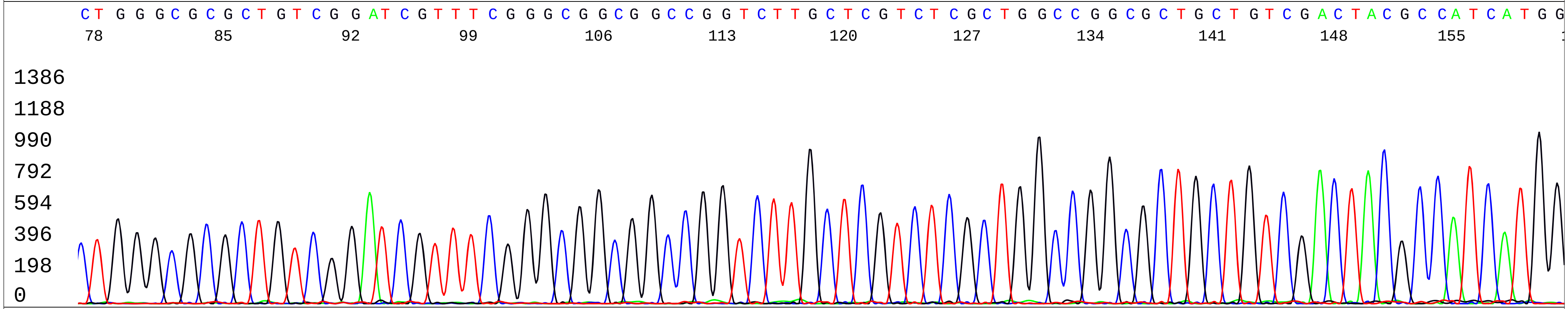

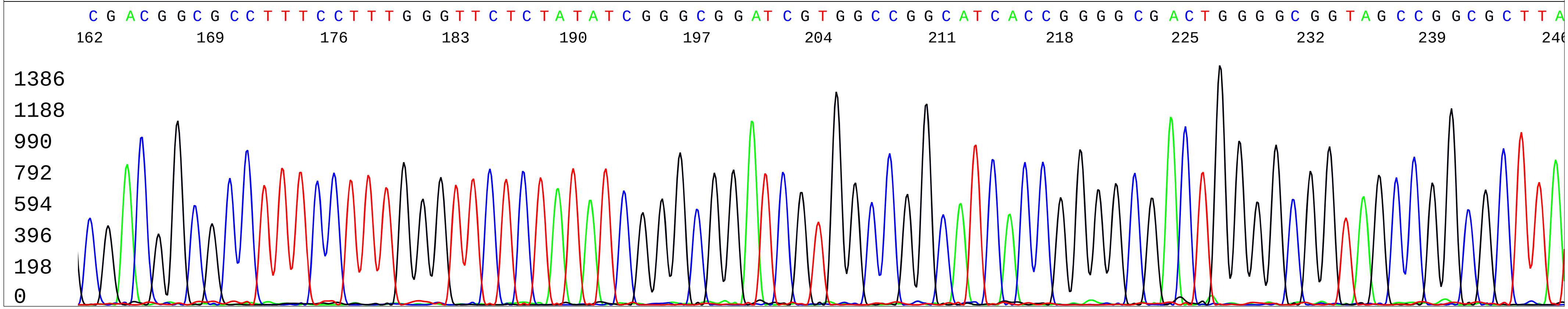

Printed on: Mon Mar 14,2011 08:19AM, GMT+03:00

Applied Biosystems

S/N G:402 A:148 T:259 C:326
KB.bcp
KB 1.4.0 Cap:3

Dr.Suleiman_2011-03-13_TetAF_C11
TetAF
KB_3130_POP7_BDTv3.mob
Pts 2771 to 8532 Pk1 Loc:2740
Version 5.3 HiSQV Bases: 503

Inst Model/Name 3100/3130RCF-19348-006
Mar 13,2011 04:52PM, GMT+03:00
Mar 13,2011 05:03PM, GMT+03:00
Spacing:11.37 Pts/Panel1000
Plate Name: Dr.Suleiman

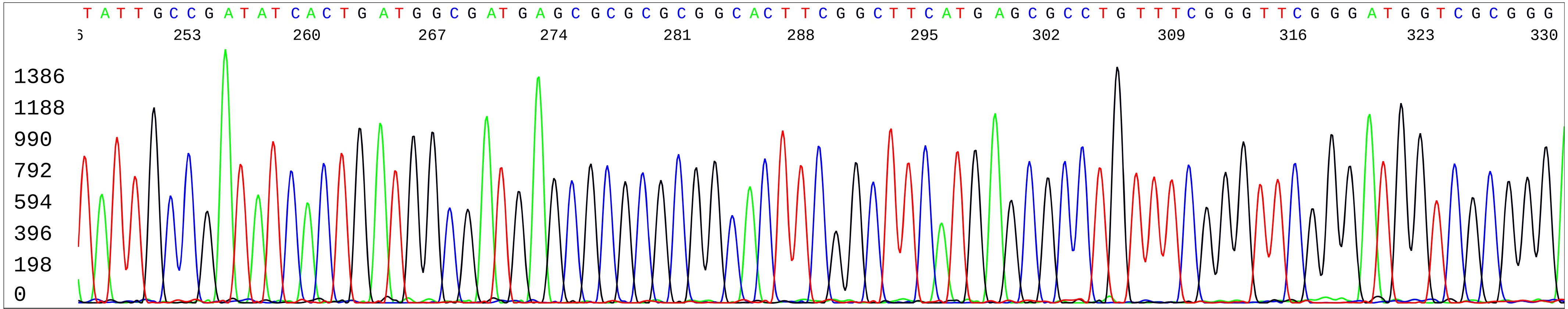

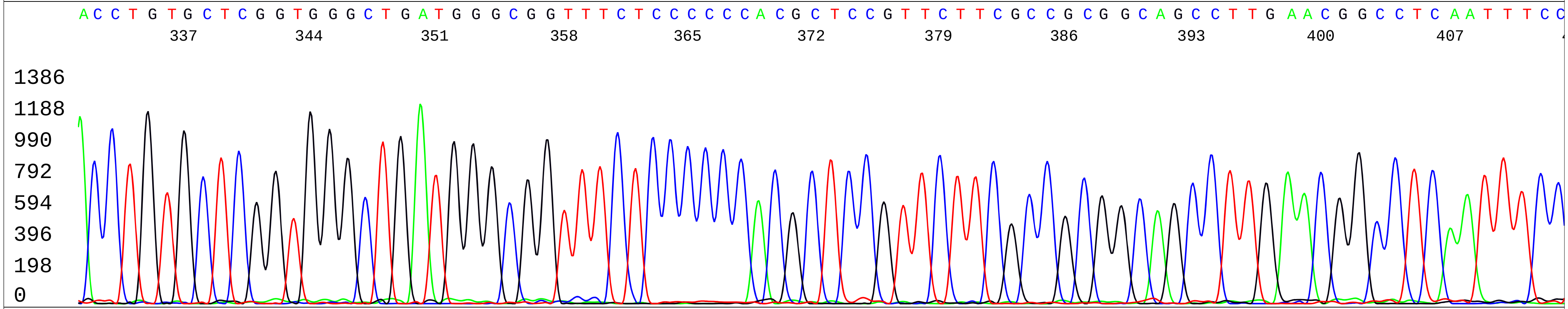

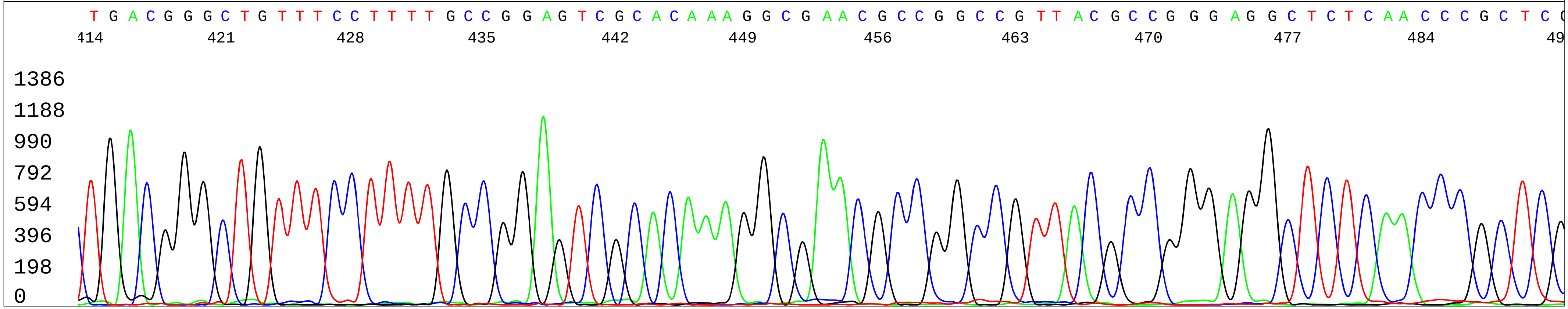

S/N G:402 A:148 T:259 C:326
KB.bcp
KB 1.4.0 Cap:3

Dr.Suleiman_2011-03-13_TetAF_C11
TetAF
KB_3130_POP7_BDTv3.mob
Pts 2771 to 8532 Pk1 Loc:2740
Version 5.3 HiSQV Bases: 503

Inst Model/Name 3100/3130RCF-19348-006
Mar 13,2011 04:52PM, GMT+03:00
Mar 13,2011 05:03PM, GMT+03:00
Spacing:11.37 Pts/Panel1000
Plate Name: Dr.Suleiman

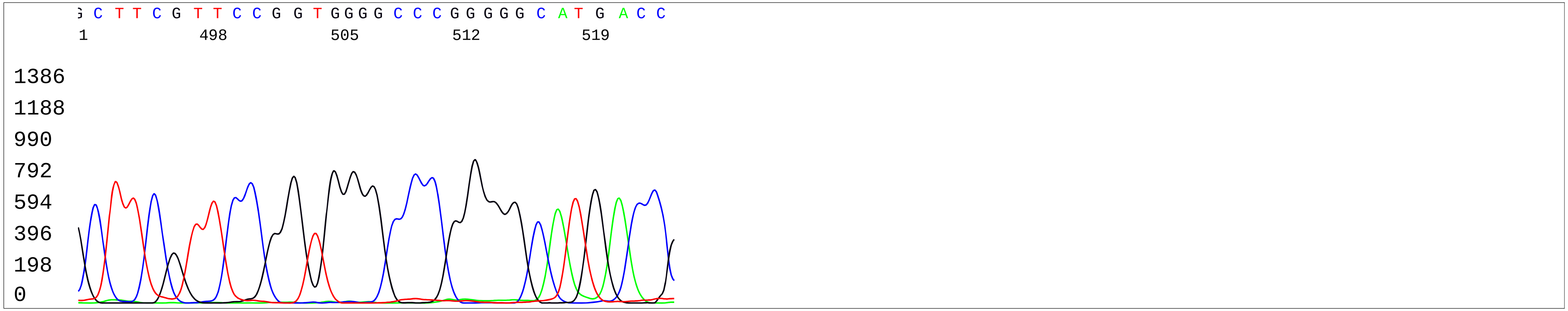

Supplement: Additional file 1: Table S1. — Specimen types and Demographics of E. coli O25b-B2-ST131 isolates. Samples from pus, skin and wound have been illustrated under soft tissue. [file 12866_2014_214_MOESM1_ESM.zip › 12866_2014_214_MOESM1_ESM/12866_2014_214_add26.pdf]
