# Supplementary material for: The characterization and antibiotic resistance profiles of clinical Escherichia coli O25b-B2-ST131 isolates in Kuwait
Source: BMC Microbiol. 2014 Aug 28;14:214. doi: 10.1186/s12866-014-0214-6 (PMC4159528; doi:10.1186/s12866-014-0214-6)
Supplement: Additional file 1: Table S1. — Specimen types and Demographics of E. coli O25b-B2-ST131 isolates. Samples from pus, skin and wound have been illustrated under soft tissue. [file 12866_2014_214_MOESM1_ESM.zip › 12866_2014_214_MOESM1_ESM/12866_2014_214_add27.pdf]

S/N G:2690 A:2996 T:1370 C:1380
KB.bcp
KB 1.4.0 Cap:3

Dr.Suleiman_2011-03-07_BLAOXAF_C03
BLAOXAF
KB_3130_POP7_BDTv3.mob
Pts 1955 to 8532 Pk1 Loc:1924
Version 5.3 HiSQV Bases: 295

Inst Model/Name 3100/3130RCF-19348-006
Mar 07,2011 06:14PM, GMT+03:00
Mar 07,2011 06:25PM, GMT+03:00
Spacing:10.61
Plate Name: Dr.Suleiman

```
  1   GAGGCAATCA  TACACCAAAG  ACGTGGATGC  ATTTTCTGTT  GTTTGGGTTT  CGCAAGAAAT  AACCCAAAAA  ATTGGATTAA    80
 81   ATAAAATCAA  GAATTATCTC  AAAGATTTTG  ATTATGGAAA  TCAAGACTTC  TCTGGAGATA  AAGAAAGAAA  CAACGGATTA   160
161   ACAGAAGCAT  GGCTCGAAAG  TAGCTTAAAA  ATTTCACCAG  AAGAACAAAT  TCAATTCCTG  CGTAAAATTA  TTAATCACAA   240
241   TCTCCCAGTT  AAAAACTCAG  CCATAGAAAA  CACCATAGAG  AACATGTATC  TACAAGATCT  GGATAATAGT  ACAAAACTGT   320
321   ATGGGAA                                                                                         327
```

Applied Biosystems

S/N G:2690 A:2996 T:1370 C:1380
KB.bcp
KB 1.4.0 Cap:3

Dr.Suleiman_2011-03-07_BLAOXAF_C03
BLAOXAF
KB_3130_POP7_BDTv3.mob
Pts 1955 to 8532 Pk1 Loc:1924
Version 5.3 HiSQV Bases: 295

Inst Model/Name 3100/3130RCF-19348-006
Mar 07,2011 06:14PM, GMT+03:00
Mar 07,2011 06:25PM, GMT+03:00
Spacing:10.61 Pts/Panel1000
Plate Name: Dr.Suleiman

G AGG CAA TCA T ACACC AAAG ACG TGG ATG C A T T T T T C T G T T G T T T G G G T T T C G C A A G A A A T A A C C C A A A A A A T T G G A T T A A A T

1 4 7 10 13 16 19 22 25 28 31 34 37 40 43 46 49 52 55 58 61 64 67 70 73 76 79 82

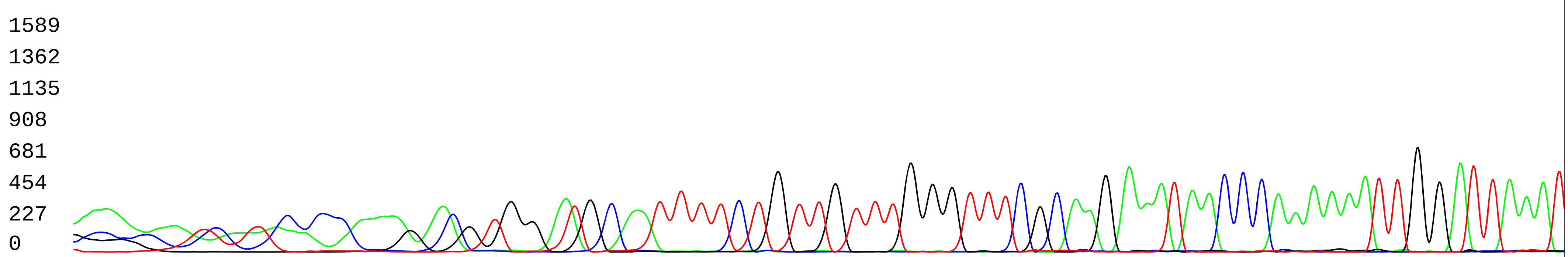

A A A A T C A A G A A T T A T C T C A A A G A T T T T G A T T A T G G A A A T C A A G A C T T C T C T G G A G A T A A A G A A A G A A A C A A C G G A T T A A C A G A

85 88 91 94 97 100 103 106 109 112 115 118 121 124 127 130 133 136 139 142 145 148 151 154 157 160 163 1

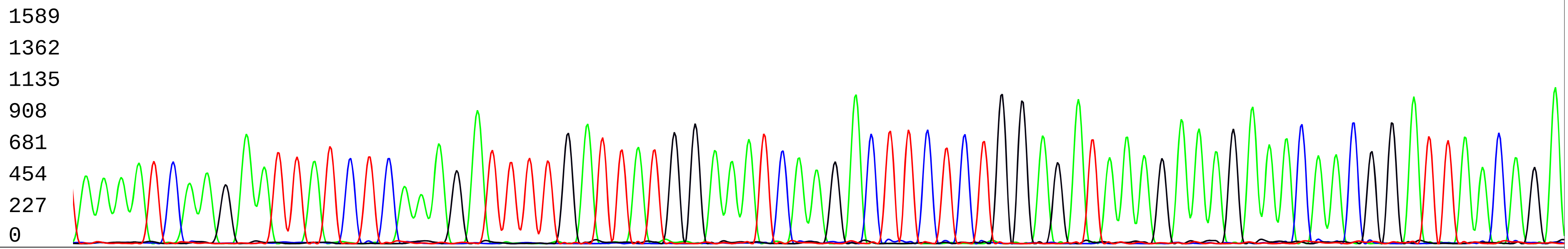

A G C A T G G C T C G A A A G T A G C T T A A A A A T T T C A C C A G A A G A A C A A A T T C A A T T C C T G C G T A A A A T T A T T A A T C A C A A T C T C C C A G T

166 169 172 175 178 181 184 187 190 193 196 199 202 205 208 211 214 217 220 223 226 229 232 235 238 241 244 247

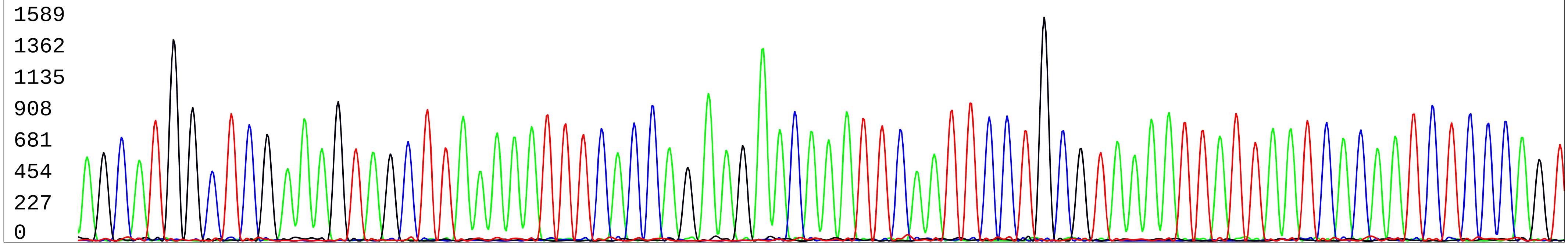

Applied Biosystems

S/N G:2690 A:2996 T:1370 C:1380
KB.bcp
KB 1.4.0 Cap:3

Dr.Suleiman_2011-03-07_BLAOXAF_C03
BLAOXAF
KB_3130_POP7_BDTv3.mob
Pts 1955 to 8532 Pk1 Loc:1924
Version 5.3 HiSQV Bases: 295

Inst Model/Name 3100/3130RCF-19348-006
Mar 07,2011 06:14PM, GMT+03:00
Mar 07,2011 06:25PM, GMT+03:00
Spacing:10.61 Pts/Panel1000
Plate Name: Dr.Suleiman

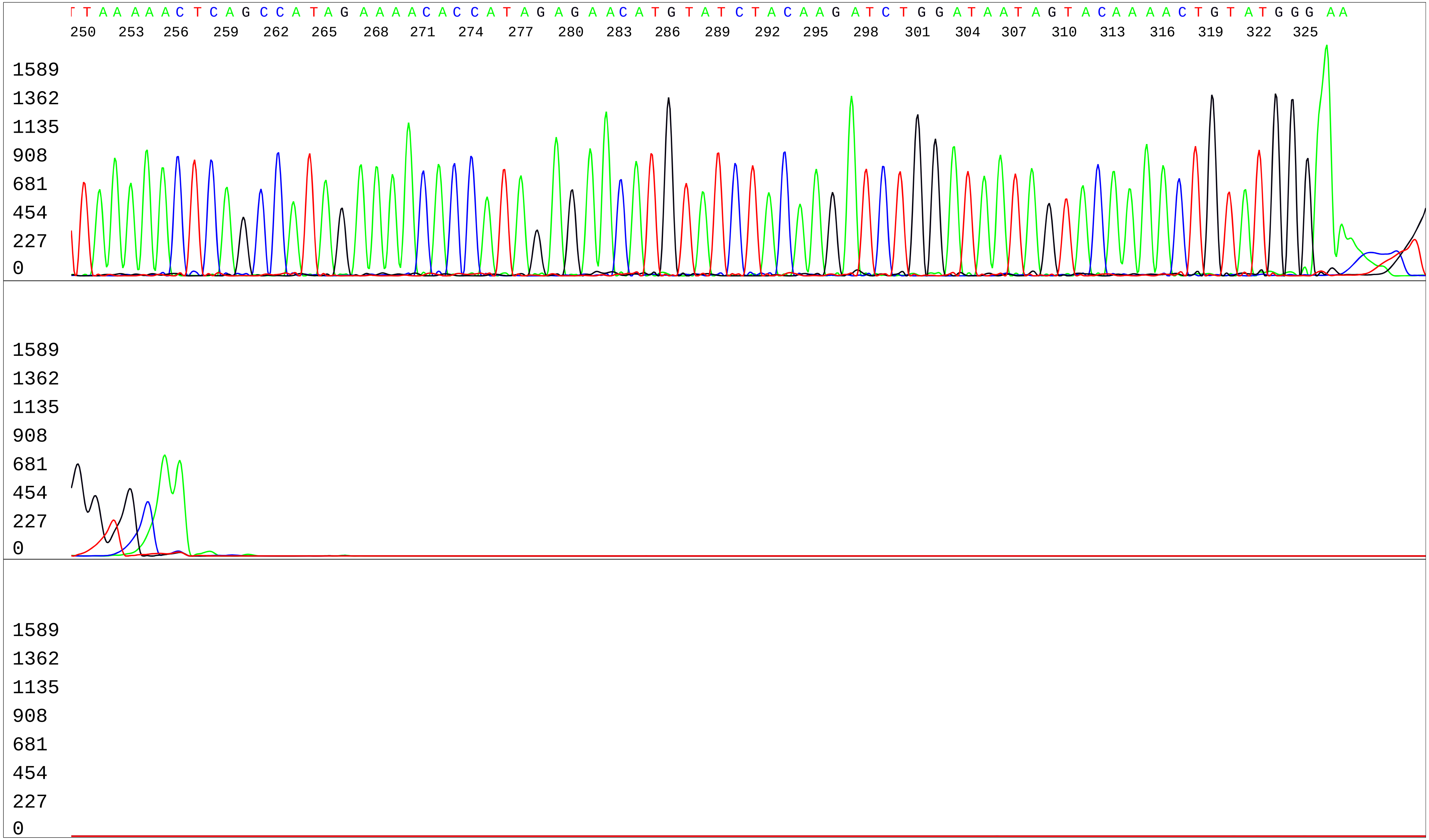

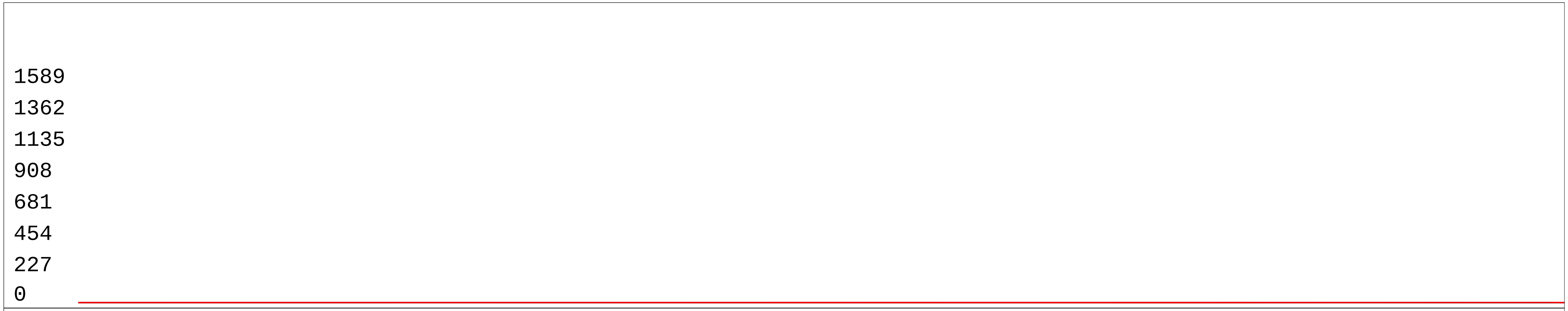
1589
1362
1135
908
681
454
227
0

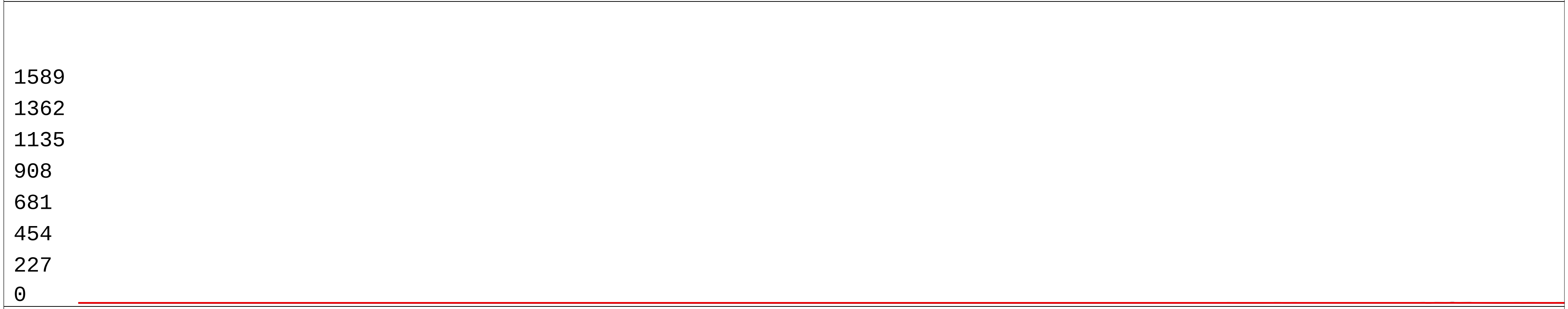
1589
1362
1135
908
681
454
227
0

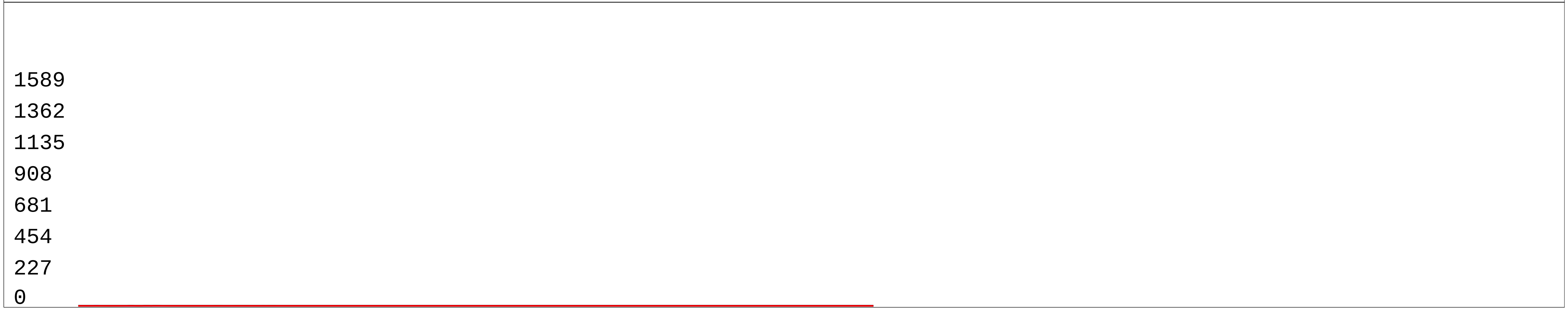
1589
1362
1135
908
681
454
227
0
