# Supplementary material for: The characterization and antibiotic resistance profiles of clinical Escherichia coli O25b-B2-ST131 isolates in Kuwait
Source: BMC Microbiol. 2014 Aug 28;14:214. doi: 10.1186/s12866-014-0214-6 (PMC4159528; doi:10.1186/s12866-014-0214-6)

Applied Biosystems

S/N G:268 A:398 T:339 C:232	Dr.Suleiman_2011-03-13_BlaoxaF_G10	Inst Model/Name 3100/3130RCF-19348-006
	BlaoxaF	Mar 13,2011 04:22PM, GMT+03:00
	KB_3130_POP7_BDTv3.mob	Mar 13,2011 04:32PM, GMT+03:00
KB.bcp	Pts 2615 to 8532 Pk1 Loc:2584	Spacing:12.63
KB 1.4.0 Cap:3	Version 5.3 HiSQV Bases: 298	Plate Name: Dr.Suleiman

```
  1  ACATACCCCA AAGACGTGGA TGCAATTTTC TGTTGTTTGG GTTTCGCAAG AAATAACCCA AAAAATTGGA TTAAATAAAA   80
 81  TCAAGAATTA TCTCAAAGAT TTTGATTATG GAAATCAAGA CTTCTCTGGA GATAAAGAAA GAAACAACGG ATTAACAGAA  160
161  GCATGGCTCG AAAGTAGCTT AAAAATTTCA CCAGAAGAAC AAATTCAATT CCTGCGTAAA ATTATTAATC ACAATCTCCC  240
241  AGTTAAAAAC TCAGCCATAG AAAACACCAT AGAGAACATG TATCTACAAG ATCTGGATAA TAGTACAAAA CTGTATGGGA  320
321  AAACATCGGG GTGGCAA                                                                      337
```

Applied Biosystems

S/N G:268 A:398 T:339 C:232
KB.bcp
KB 1.4.0 Cap:3

Dr.Suleiman_2011-03-13_BlaoxaF_G10
BlaoxaF
KB_3130_POP7_BDTv3.mob
Pts 2615 to 8532 Pk1 Loc:2584
Version 5.3 HiSQV Bases: 298

Inst Model/Name 3100/3130RCF-19348-006
Mar 13,2011 04:22PM, GMT+03:00
Mar 13,2011 04:32PM, GMT+03:00
Spacing:12.63 Pts/Panel1000
Plate Name: Dr.Suleiman

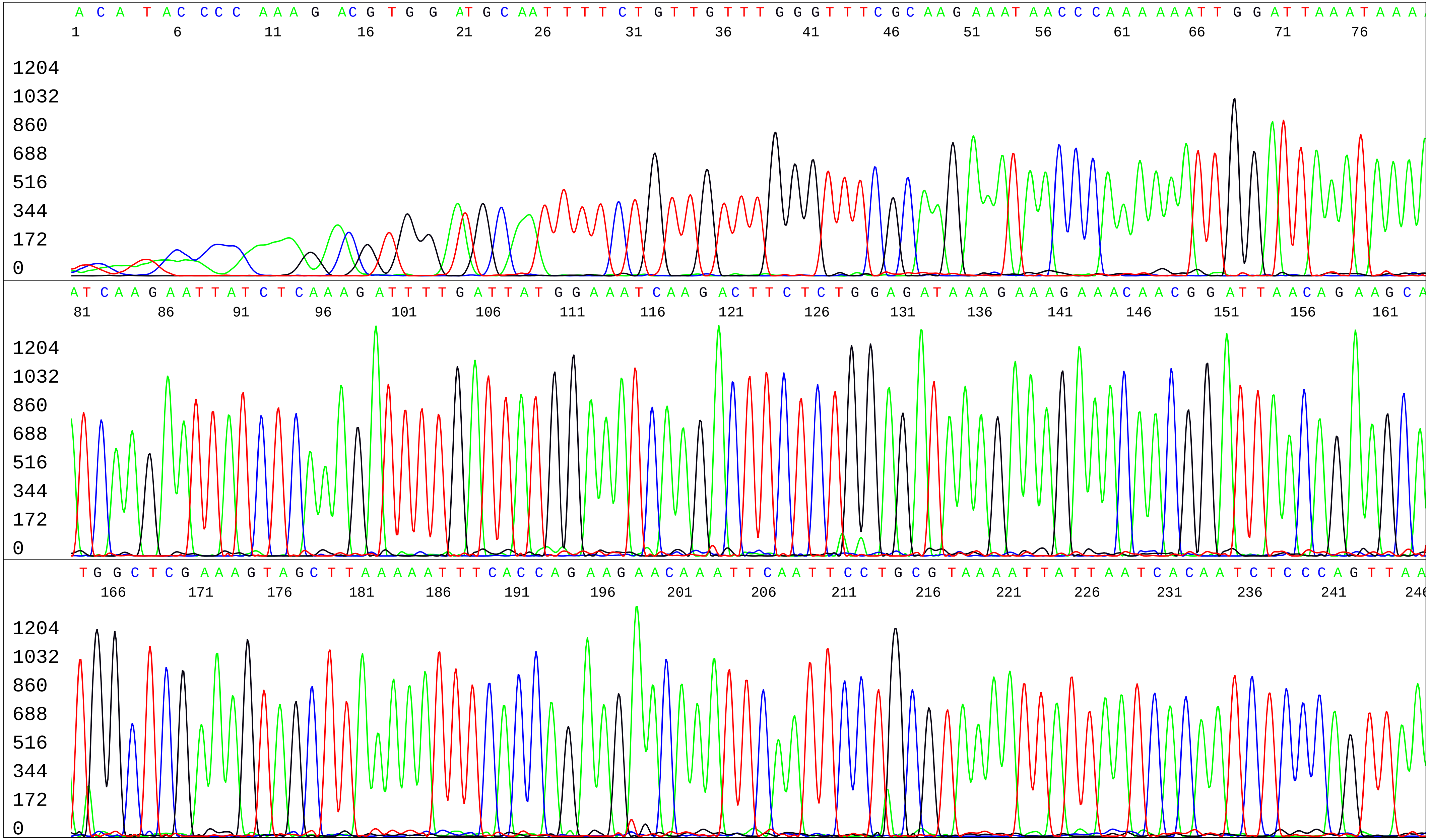

Printed on: Mon Mar 14,2011 08:15AM, GMT+03:00

S/N G:268 A:398 T:339 C:232
KB.bcp
KB 1.4.0 Cap:3

Inst Model/Name 3100/3130RCF-19348-006
Mar 13,2011 04:22PM, GMT+03:00
Mar 13,2011 04:32PM, GMT+03:00
Spacing:12.63 Pts/Panel1000
Plate Name: Dr.Suleiman

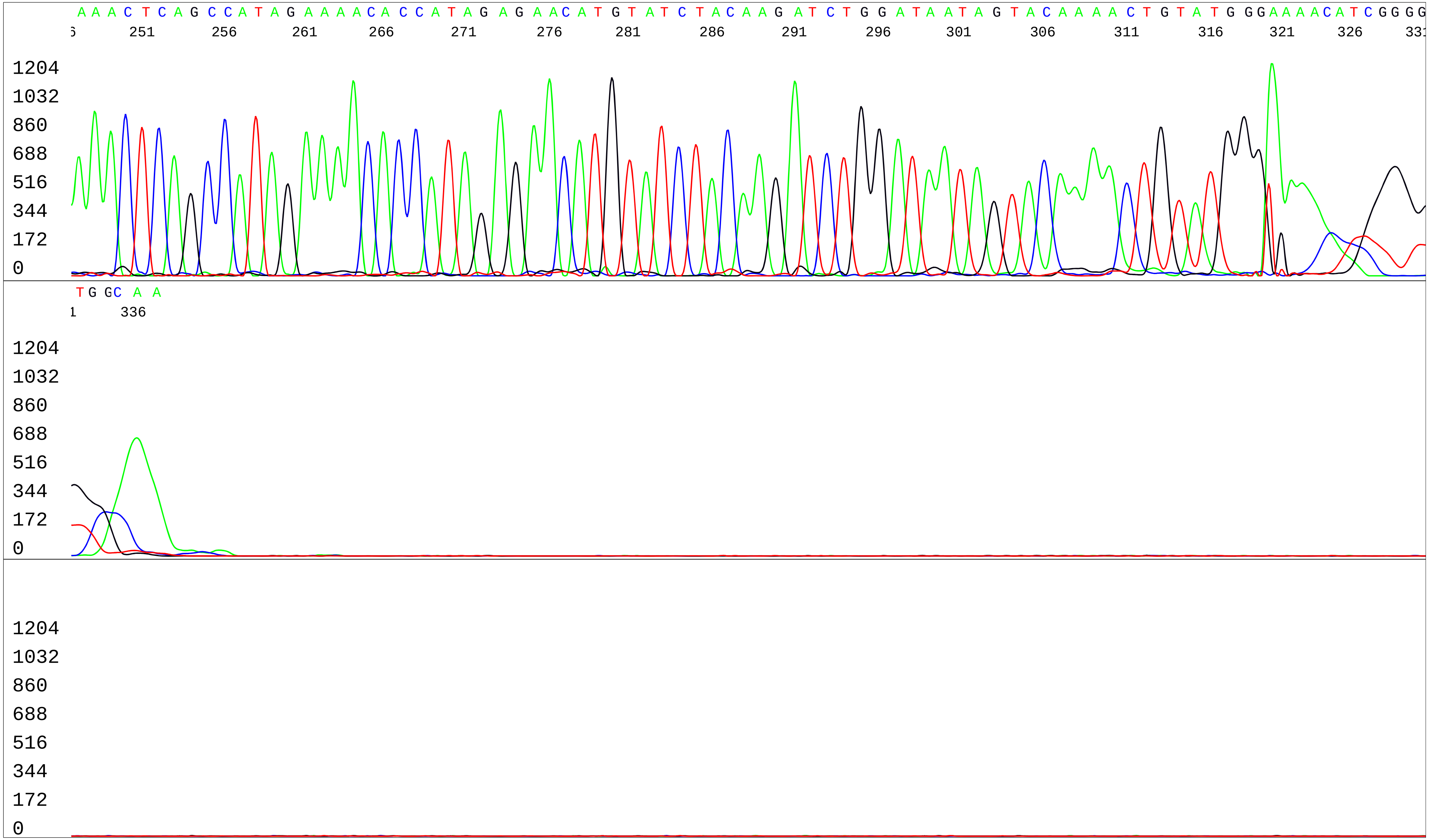

Applied Biosystems

S/N G:268 A:398 T:339 C:232
KB.bcp
KB 1.4.0 Cap:3

Dr.Suleiman_2011-03-13_BlaoxaF_G10
BlaoxaF
KB_3130_POP7_BDTv3.mob
Pts 2615 to 8532 Pk1 Loc:2584
Version 5.3 HiSQV Bases: 298

Inst Model/Name 3100/3130RCF-19348-006
Mar 13,2011 04:22PM, GMT+03:00
Mar 13,2011 04:32PM, GMT+03:00
Spacing:12.63 Pts/Panel1000
Plate Name: Dr.Suleiman

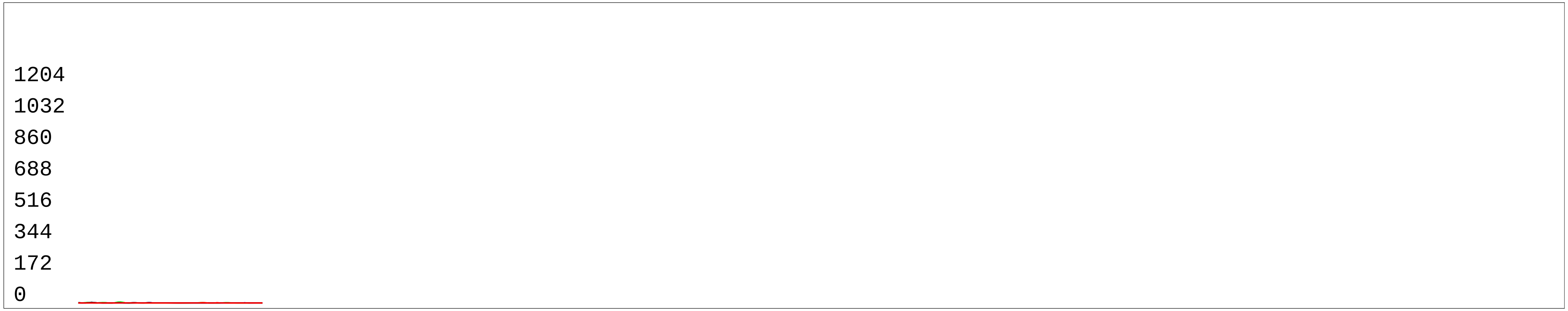

Supplement: Additional file 1: Table S1. — Specimen types and Demographics of E. coli O25b-B2-ST131 isolates. Samples from pus, skin and wound have been illustrated under soft tissue. [file 12866_2014_214_MOESM1_ESM.zip › 12866_2014_214_MOESM1_ESM/12866_2014_214_add28.pdf]
