# Supplementary material for: The characterization and antibiotic resistance profiles of clinical Escherichia coli O25b-B2-ST131 isolates in Kuwait
Source: BMC Microbiol. 2014 Aug 28;14:214. doi: 10.1186/s12866-014-0214-6 (PMC4159528; doi:10.1186/s12866-014-0214-6)

Applied Biosystems

S/N G:477 A:285 T:318 C:233
KB.bcp
KB 1.4.0 Cap:2

Dr.Suleiman_2011-03-13_OxaR_B11
OxaR
KB_3130_POP7_BDTv3.mob
Pts 2333 to 8532 Pk1 Loc:2302
Version 5.3 HiSQV Bases: 406

Inst Model/Name 3100/3130RCF-19348-006
Mar 13,2011 04:52PM, GMT+03:00
Mar 13,2011 05:03PM, GMT+03:00
Spacing:11.38
Plate Name: Dr.Suleiman

```
  1   CATTTTCCTT GTAGTGCGGA ACAAAACATA TTTATGTCCT GATTTGCTTA TAATAAACCC TTCAAACCAT CCGTTTTGTA    80
 81   AGGTTCTATT TGCTGTGAAT CCTGCACCAG TTTTCCCATA CAGTTTTGTA CTATTATCCA GATCTTGTAG ATACATGTTC   160
161   TCTATGGTGT TTTCTATGGC TGAGTTTTTA ACTGGGAGAT TGTGATTAAT AATTTTACGC AGGAATTGAA TTTGTTCTTC   240
241   TGGTGAAATT TTTAAGCTAC TTTCGAGCCA TGCTTCTGTT AATCCGTTGT TTCTTTCTTT ATCTCCAGAG AAGTCTTGAT   320
321   TTCCATAATC AAAATCTTTG AGATAATTCT TGATTTTATT TAATCCAATT TTTTGGGTTA TTTCTTGCAA AACCCAAAAA   400
401   ACAGAAAAAC CCTTTCGATT ATTTGCACTG GGGTTTTTTC TCTTTTTTTT TTTTATCAGG GTCTAGAATA TGAAGAAATA   480
481   ACACTAACAC AAAATTGGGC AGATGTTTCT AAAAATTCCA CAAACTAACA CTCTACGGTG CAATAAAGTT TAAAAATATT   560
561   CCCG                                                                                     564
```

Applied Biosystems

S/N G:477 A:285 T:318 C:233
KB.bcp
KB 1.4.0 Cap:2

Dr.Suleiman_2011-03-13_OxaR_B11
OxaR
KB_3130_POP7_BDTv3.mob
Pts 2333 to 8532 Pk1 Loc:2302
Version 5.3 HiSQV Bases: 406

Inst Model/Name 3100/3130RCF-19348-006
Mar 13,2011 04:52PM, GMT+03:00
Mar 13,2011 05:03PM, GMT+03:00
Spacing:11.38 Pts/Panel1000
Plate Name: Dr.Suleiman

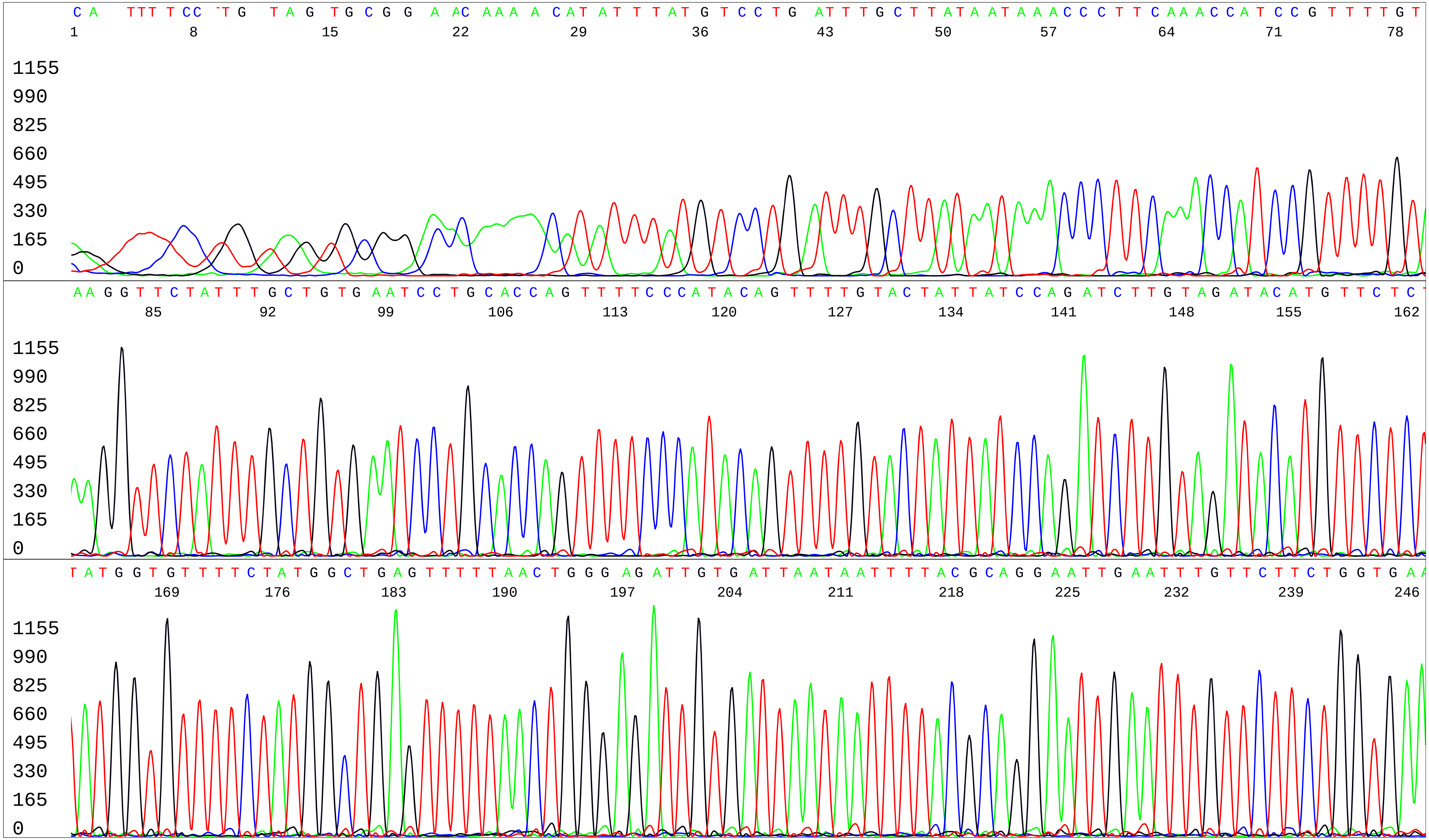

Printed on: Mon Mar 14,2011 08:19AM, GMT+03:00

Applied Biosystems

S/N G:477 A:285 T:318 C:233
KB.bcp
KB 1.4.0 Cap:2

Dr.Suleiman_2011-03-13_OxaR_B11
OxaR
KB_3130_POP7_BDTv3.mob
Pts 2333 to 8532 Pk1 Loc:2302
Version 5.3 HiSQV Bases: 406

Inst Model/Name 3100/3130RCF-19348-006
Mar 13,2011 04:52PM, GMT+03:00
Mar 13,2011 05:03PM, GMT+03:00
Spacing:11.38 Pts/Panel1000
Plate Name: Dr.Suleiman

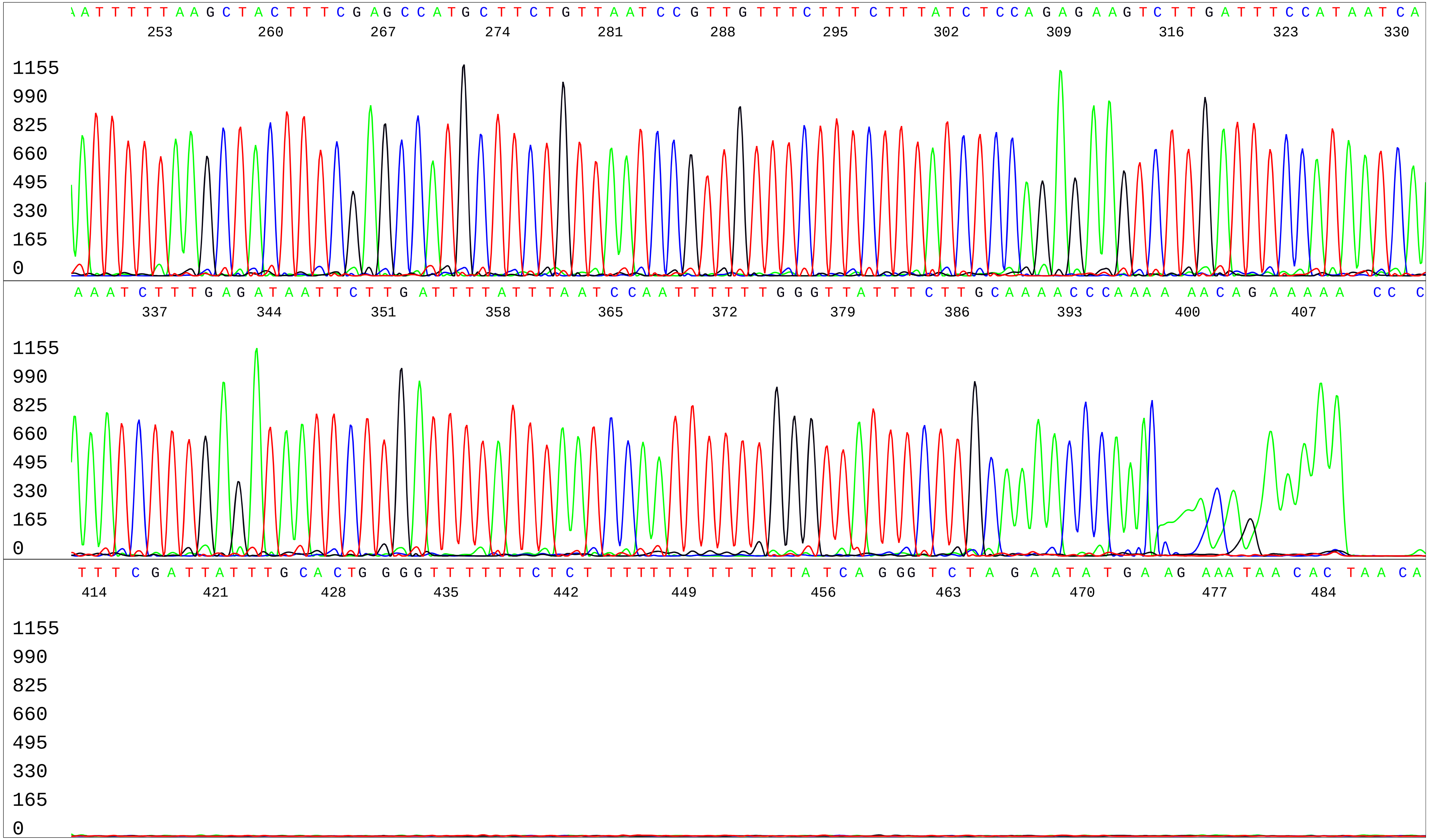

S/N G:477 A:285 T:318 C:233
KB.bcp
KB 1.4.0 Cap:2

Dr.Suleiman_2011-03-13_OxaR_B11
OxaR
KB_3130_POP7_BDTv3.mob
Pts 2333 to 8532 Pk1 Loc:2302
Version 5.3 HiSQV Bases: 406

Inst Model/Name 3100/3130RCF-19348-006
Mar 13,2011 04:52PM, GMT+03:00
Mar 13,2011 05:03PM, GMT+03:00
Spacing:11.38 Pts/Panel1000
Plate Name: Dr.Suleiman

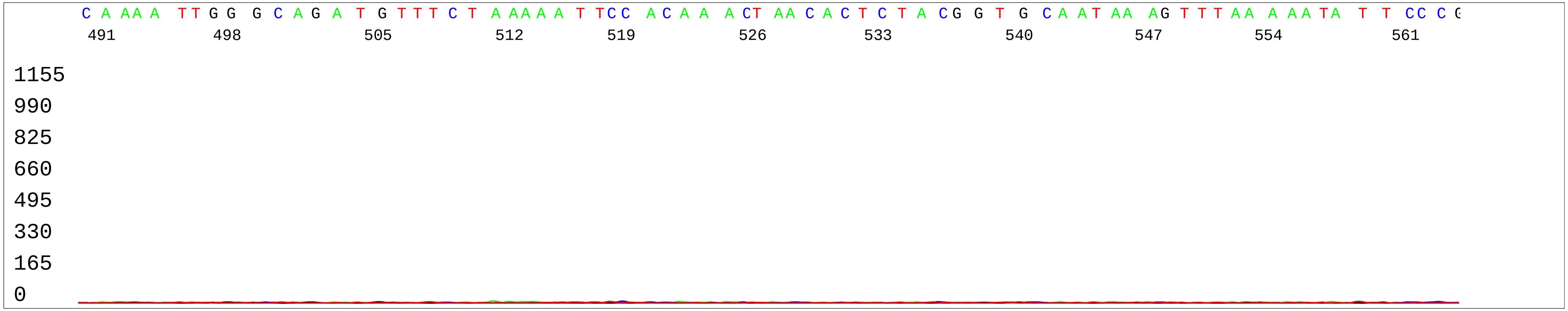

Supplement: Additional file 1: Table S1. — Specimen types and Demographics of E. coli O25b-B2-ST131 isolates. Samples from pus, skin and wound have been illustrated under soft tissue. [file 12866_2014_214_MOESM1_ESM.zip › 12866_2014_214_MOESM1_ESM/12866_2014_214_add29.pdf]
