# Supplementary material for: The characterization and antibiotic resistance profiles of clinical Escherichia coli O25b-B2-ST131 isolates in Kuwait
Source: BMC Microbiol. 2014 Aug 28;14:214. doi: 10.1186/s12866-014-0214-6 (PMC4159528; doi:10.1186/s12866-014-0214-6)

Applied Biosystems

121-R_3130POP7_v3.1_2012-10-21
121-R
KB_3130_POP7_BDTv3.mob
Pts 1861 to 8532 Pk1 Loc:1830
Version 5.3 HiSQV Bases: 645

Inst Model/Name 3100/3130GeneticAnalyzer-19348-006
Oct 21,2012 04:06PM, AST
Oct 21,2012 04:17PM, AST
Spacing:10.42
Plate Name: SS-21102012

S/N G:1913 A:1178 T:972 C:1172
KB.bcp
KB 1.4.0 Cap:2

```
  1  ATTCGTCATC CATAGTTGCC TGACTCCCCG TCGTGTAGAT AACTACGATA CGGGAGGGCT TACCATCTGG   70
 71  CCCCAGTGCT GCAATGATAC CGCGAGACCC ACGCTCACCG GCTCCAGATT TATCAGCAAT AAACCAGCCA  140
141  GCCGGAAGGG CCGAGCGCAG AAGTGGTCCT GCAACTTTAT CCGCCTCCAT CCAGTCTATT AATTGTTGCC  210
211  GGGAAGCTAG AGTAAGTAGT TCGCCAGTTA ATAGTTTGCG CAACGTTGTT GCCATTGCTG CAGGCATCGT  280
281  GGTGTCACGC TCGTCGTTTG GTATGGCTTC ATTCAGCTCC GGTTCCCAAC GATCAAGGCG AGTTACATGA  350
351  TCCCCCATGT TGTGCAAAAA AGCGGTTAGC TCCTTCGGTC CTCCGATCGT TGTCAGAAGT AAGTTGGCAG  420
421  CAGTGTTATC ACTCATGGTT ATGGCAGCAC TGCATAATTC TCTTACTGTC ATGCCATCCG TAAGATGCTT  490
491  TTCTGTGACT GGTGAGTACT CAACCAAGTC ATTCTGAGAA TAGTGTATGC GGCGACCGAG TTGCTCTTGC  560
561  CCGGCGTCAA CACGGGATAA TACCGCACCA CATAGCAGAA CTTTAAAAGT GCTCATCATT GGAAAACGTT  630
631  CTTCGGGGCG AAAACTCTCA AGGATCTTTA CCGCTGTTGA GATCCCAGTT C                     681
```

Applied Biosystems

121-R_3130POP7_v3.1_2012-10-21
121-R
KB_3130_POP7_BDTv3.mob
Pts 1861 to 8532 Pk1 Loc:1830
Version 5.3 HiSQV Bases: 645

Inst Model/Name 3100/3130GeneticAnalyzer-19348-006
Oct 21,2012 04:06PM, AST
Oct 21,2012 04:17PM, AST
Spacing:10.42 Pts/Panel1500
Plate Name: SS-21102012

S/N G:1913 A:1178 T:972 C:1172
KB.bcp
KB 1.4.0 Cap:2

ATT C G T CA TCCATAG T TG CC TG AC TCCCCG TCG TG TAG ATAACTACG ATACGGG AGGGCTTACCATCTG GCCCCAG TGCTGCAATGATACCGCG AGACCCACGCTCACCGGCTCCAG
1 12 23 34 45 56 67 78 89 100 111

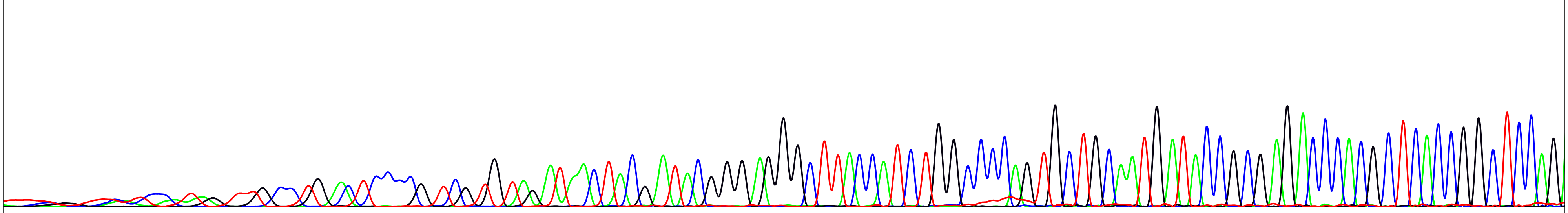

ATTTATCAGCAATAAACCAGCCAGCCGGAAGGGCCGAGCGCAGAAGTGGTCCTGCAACTTTATCCGCCTCCATCCAGTCTATTAATTGTTGCCGGGAAGCTAGAGTAAGTAGTTCGCCAGTTAAT
122 133 144 155 166 177 188 199 210 221 232 24

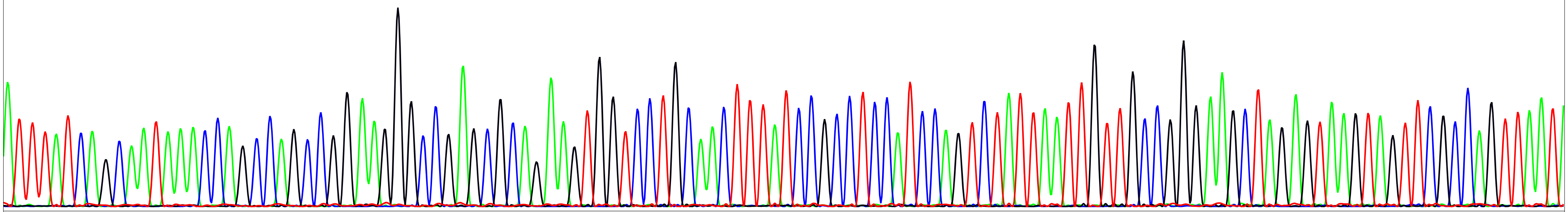

AGTTTGCGCAACGTTGTTGCCATTGCTGCAGGCATCGTGGTGTCACGCTCGTCGTTTGGTATGGCTTCATTCAGCTCCGGTTCCCAACGATCAAGGCGAGTTACATGATCCCCCATGTTGTGCAAAA
43 254 265 276 287 298 309 320 331 342 353 364

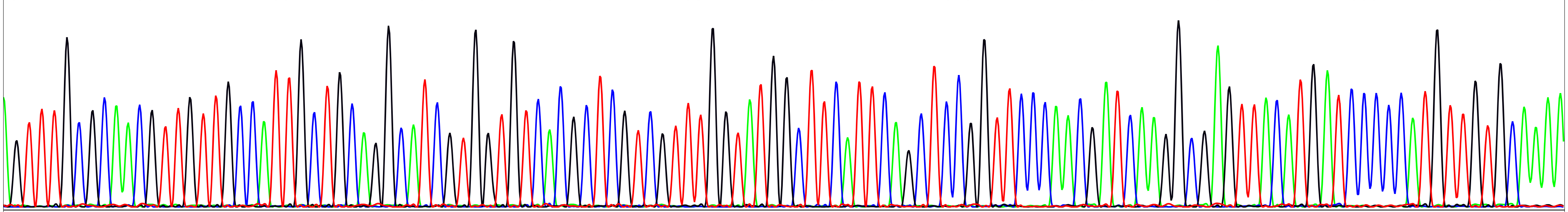

AAAGCGGTTAGCTCCTTCGGTCCTCCGATCGTTGTCAGAAGTAAGTTGGCAGCAGTGTTATCACTCATGGTTATGGCAGCACTGCATAATTCTCTTACTGTCATGCCATCCGTAAGATGCTTTTCT
375 386 397 408 419 430 441 452 463 474 485

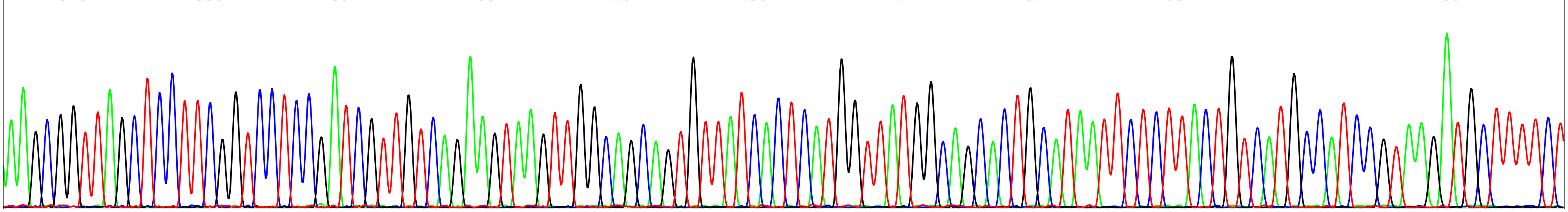

Applied Biosystems

S/N G:1913 A:1178 T:972 C:1172
KB.bcp
KB 1.4.0 Cap:2

121-R_3130POP7_v3.1_2012-10-21
121-R
KB_3130_POP7_BDTv3.mob
Pts 1861 to 8532 Pk1 Loc:1830
Version 5.3 HiSQV Bases: 645

Inst Model/Name 3100/3130GeneticAnalyzer-19348-006
Oct 21,2012 04:06PM, AST
Oct 21,2012 04:17PM, AST
Spacing:10.42 Pts/Panel1500
Plate Name: SS-21102012

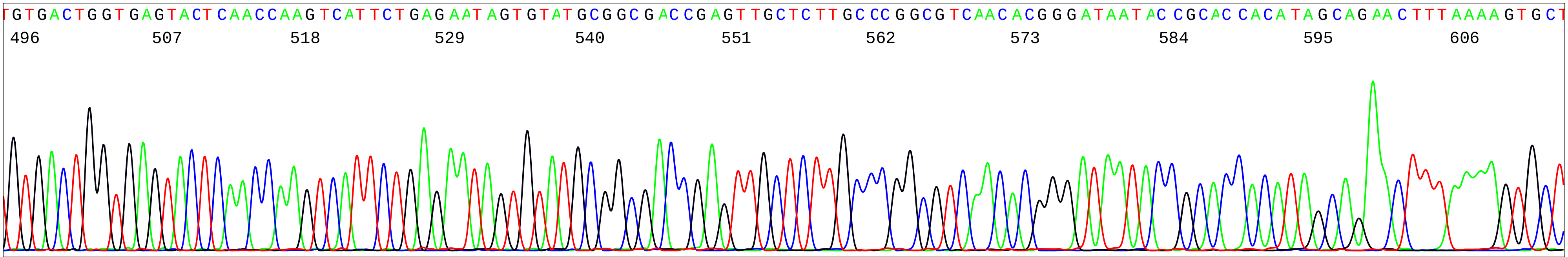

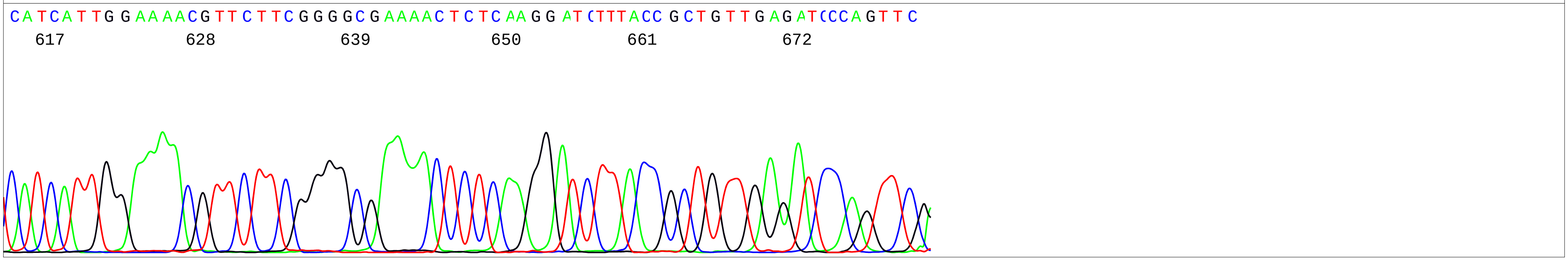

Supplement: Additional file 1: Table S1. — Specimen types and Demographics of E. coli O25b-B2-ST131 isolates. Samples from pus, skin and wound have been illustrated under soft tissue. [file 12866_2014_214_MOESM1_ESM.zip › 12866_2014_214_MOESM1_ESM/12866_2014_214_add3.pdf]
