# Supplementary material for: The characterization and antibiotic resistance profiles of clinical Escherichia coli O25b-B2-ST131 isolates in Kuwait
Source: BMC Microbiol. 2014 Aug 28;14:214. doi: 10.1186/s12866-014-0214-6 (PMC4159528; doi:10.1186/s12866-014-0214-6)

Applied Biosystems

S/N G:12 A:8 T:8 C:9
KB.bcp
KB 1.4.0 Cap:3

C1INT1F_3130POP7_v3.1_2014-02-10
C1INT1F
KB_3130_POP7_BDTv3.mob
Pts 3312 to 11071 Pk1 Loc:3289
Version 5.3 HiSQV Bases: 378

Inst Model/Name 3100/3130GeneticAnalyzer-19348-006
Feb 10,2014 09:34AM, AST
Feb 10,2014 10:06AM, AST
Spacing:-13.4
Plate Name: Shurooq

1	AAAAAATTAA	AGACAATAGG	TAAGCCATAA	ACACGCAGAA	AATTCGCTTT	TAAATAGCCC	TAGCTTTTTT	70
71	AGTACAACAG	GTTTTTACAA	CAGCCGTTAG	GGCGATATTG	ATTATATCCC	TGCAGCAAGC	CTAGACGGCT	140
141	ACCCTCTGTT	ATCTCTGCAA	ATGAAGTGCA	ACGCATTTTG	CAGGTTATGG	ATACTCGCAA	CCAAGTTATT	210
211	TTTACGCTGC	TGTATGGTGC	AAGTTTGCGC	ATTAATGAAT	GCTTGCGTTT	GCGGGTTAAA	GATTTTGATT	280
281	TTGATAATGG	CTGCATCACT	GTGCATGACG	GTAAGGGGGG	AAAGCAGAAA	CAGCCTACTG	CCCACGCGCC	350
351	TAATCCCAGC	AATAATATAA	CTCATTGAGC	AAGCGCGGCT	TATTCACAAG	ACGACAACTT	ACAAGGCGTA	420
421	GGGCCATCGC	TGCCTTTTGC	TTTAGATCAC	AAATACCCTT	CTGCTTATCG	CAGGGGGGGG	GGGATTTTTT	490
491	GCTTTCCCTC	CAGCACGCTC	TGCACCACCC	GTATAACGGC	AAATATGCCC	CATCATCTGC	ATAATCCTTG	560
561	CCAAAGGCAT	AAAAGGAACC	TAAAAAAAAA	AGGTT				595

Applied Biosystems

S/N G:12 A:8 T:8 C:9
KB.bcp
KB 1.4.0 Cap:3

C1INT1F_3130POP7_v3.1_2014-02-10
C1INT1F
KB_3130_POP7_BDTv3.mob
Pts 3312 to 11071 Pk1 Loc:3289
Version 5.3 HiSQV Bases: 378

Inst Model/Name 3100/3130GeneticAnalyzer-19348-006
Feb 10,2014 09:34AM, AST
Feb 10,2014 10:06AM, AST
Spacing:-13.4 Pts/Panel1500
Plate Name: Shurooq

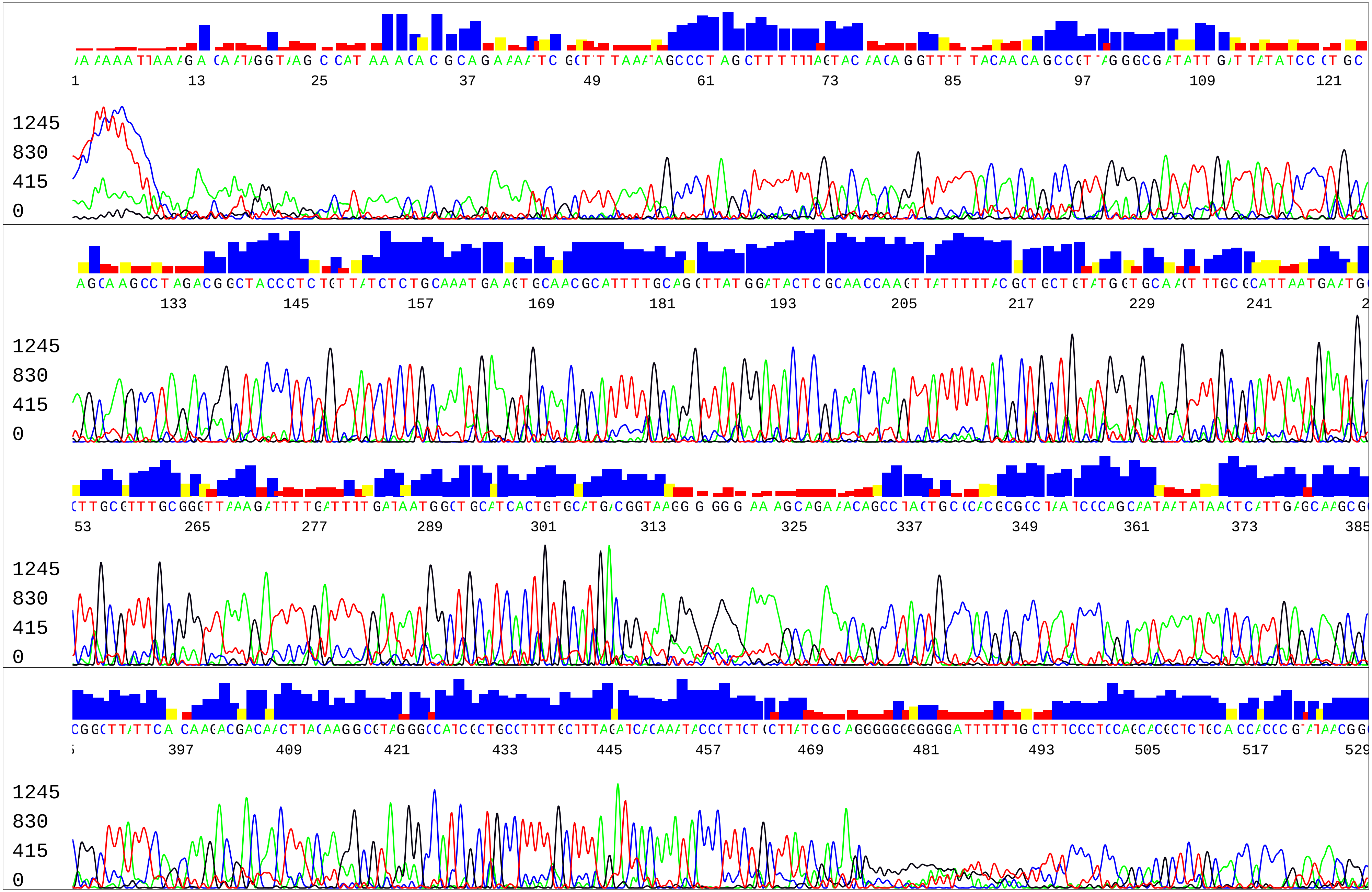

C1INT1F_3130POP7_v3.1_2014-02-10
C1INT1F
KB_3130_POP7_BDTv3.mob
Pts 3312 to 11071 Pk1 Loc:3289
Version 5.3 HiSQV Bases: 378

Inst Model/Name 3100/3130GeneticAnalyzer-19348-006
Feb 10,2014 09:34AM, AST
Feb 10,2014 10:06AM, AST
Spacing:-13.4 Pts/Panel1500
Plate Name: Shurooq

S/N G:12 A:8 T:8 C:9
KB.bcp
KB 1.4.0 Cap:3

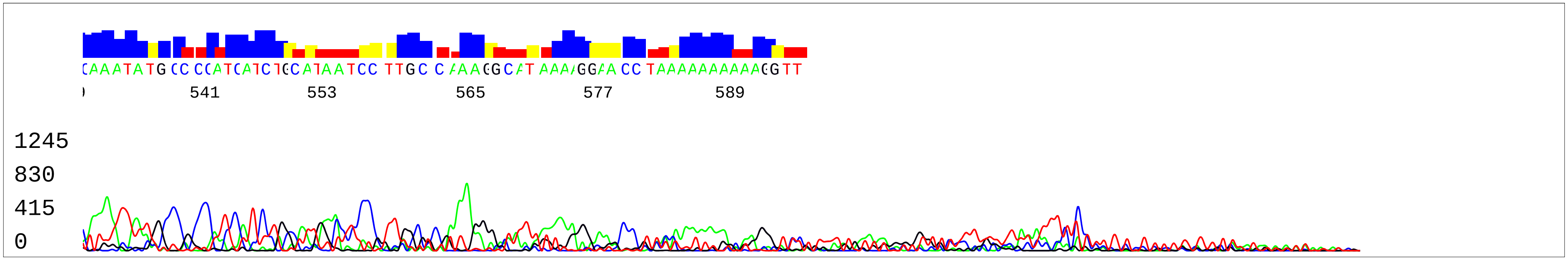

Supplement: Additional file 1: Table S1. — Specimen types and Demographics of E. coli O25b-B2-ST131 isolates. Samples from pus, skin and wound have been illustrated under soft tissue. [file 12866_2014_214_MOESM1_ESM.zip › 12866_2014_214_MOESM1_ESM/12866_2014_214_add30.pdf]
