# Supplementary material for: The characterization and antibiotic resistance profiles of clinical Escherichia coli O25b-B2-ST131 isolates in Kuwait
Source: BMC Microbiol. 2014 Aug 28;14:214. doi: 10.1186/s12866-014-0214-6 (PMC4159528; doi:10.1186/s12866-014-0214-6)

Applied Biosystems

S/N G:12 A:9 T:7 C:8
KB.bcp
KB 1.4.0 Cap:4

C1INT1R_3130POP7_v3.1_2014-02-10
C1INT1R
KB_3130_POP7_BDTv3.mob
Pts 3327 to 11071 Pk1 Loc:3304
Version 5.3 HiSQV Bases: 223

Inst Model/Name 3100/3130GeneticAnalyzer-19348-006
Feb 10,2014 09:34AM, AST
Feb 10,2014 10:06AM, AST
Spacing:-13.4
Plate Name: Shurooq

1	TGAATAACGT	ACAATCGTGA	GCCATCAAAC	ACGCAGTAAT	ATCAGCTTTA	AATAGCCCTA	GCTTTTCTAG	70
71	TACAACAGAG	TTTTACAACA	GCCGTTAGGG	CGATATTGAT	TATTCCCTGC	AAGCAAGCCT	AGACAGCTAC	140
141	CCTCTAGTTA	TCTCTGCAAA	TGAAGTAGCA	ACGCATTTTG	CAAGTTAAGG	ATACTCGCAA	CCAAGTTATT	210
211	TTTACGCTGC	TGTATGGAGC	AAGTTTGCGC	ATTAATGAAT	GCTTGCGTTT	GCGGGTTAAG	ATTTTGATTT	280
281	GAAATGGCTG	CATCACTGTG	CATGACGGTA	AAAAATGGTA	AACAGAAACA	GCCTACTGCC	CACGCGCCTA	350
351	ATCCCAGCAA	TAAAATAACT	CATTGAGCAA	GCGCGGCTTA	TTCAGCAAGA	CGACAACTTA	CAAGGCGTAA	420
421	ACCATCGCTG	CCTTTTGCTT	TAGATCACAA	ATACCCTTCT	GCTTATCGCA	CGGGGGGGGA	ATTTTTTTGT	490
491	TTTTCCCTCC	AGCACGCTCT	GCAACCACCC	GTTAACGCAT	TTATGCCGCC	TCTCTGCTTG	ACTCCGTTGC	560
561	CCGTAGGCTT	CAAAGCAGCC	TCCAAAAGCA	AGGT				594

Applied Biosystems

S/N G:12 A:9 T:7 C:8
KB.bcp
KB 1.4.0 Cap:4

C1INT1R_3130POP7_v3.1_2014-02-10
C1INT1R
KB_3130_POP7_BDTv3.mob
Pts 3327 to 11071 Pk1 Loc:3304
Version 5.3 HiSQV Bases: 223

Inst Model/Name 3100/3130GeneticAnalyzer-19348-006
Feb 10,2014 09:34AM, AST
Feb 10,2014 10:06AM, AST
Spacing:-13.4 Pts/Panel1500
Plate Name: Shurooq

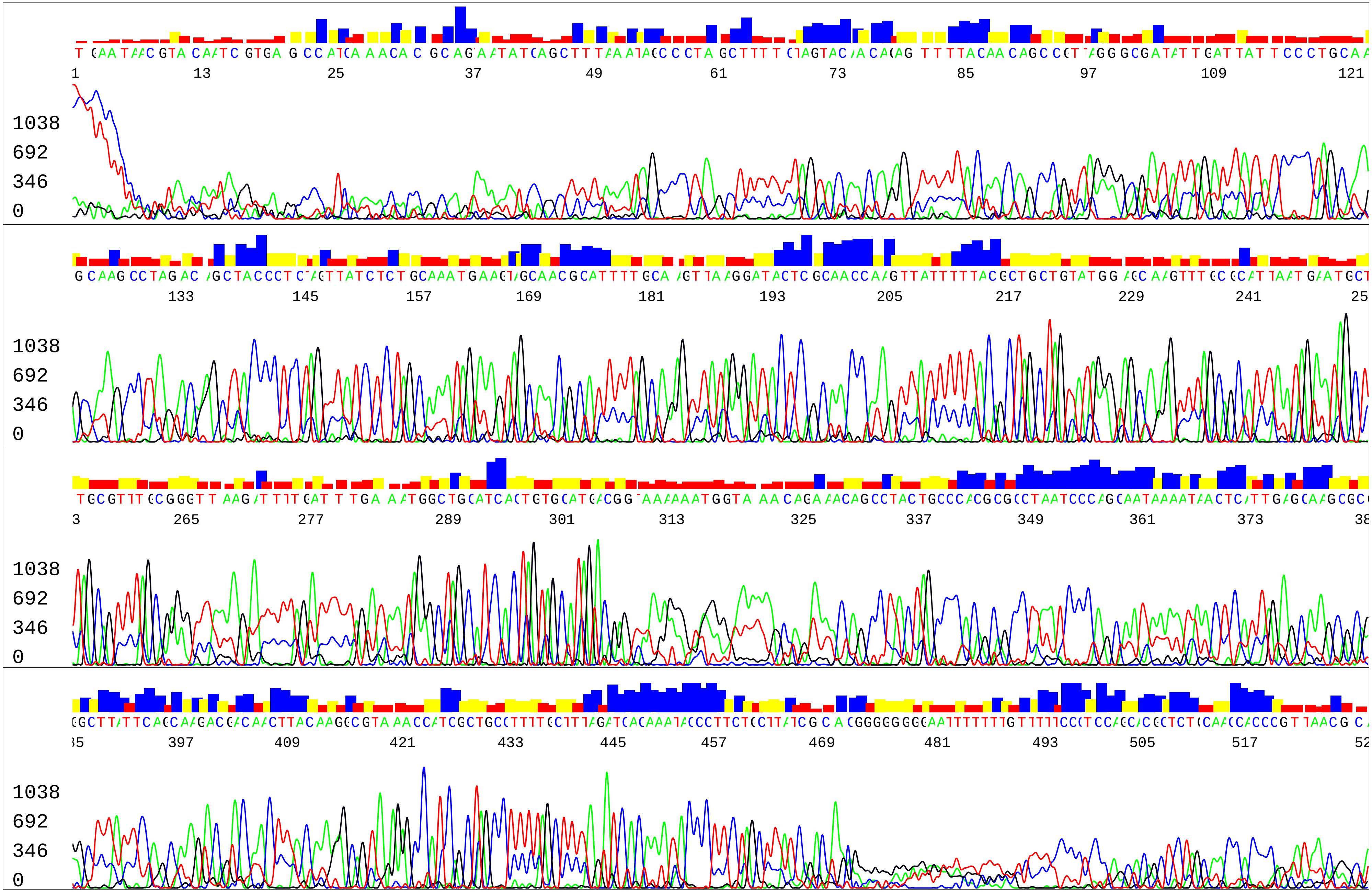

C1INT1R_3130POP7_v3.1_2014-02-10
C1INT1R
KB_3130_POP7_BDTv3.mob
Pts 3327 to 11071 Pk1 Loc:3304
Version 5.3   HiSQV Bases: 223

Inst Model/Name 3100/3130GeneticAnalyzer-19348-006
Feb 10,2014 09:34AM, AST
Feb 10,2014 10:06AM, AST
Spacing:-13.4 Pts/Panel1500
Plate Name: Shurooq

S/N G:12 A:9 T:7 C:8
KB.bcp
KB 1.4.0   Cap:4

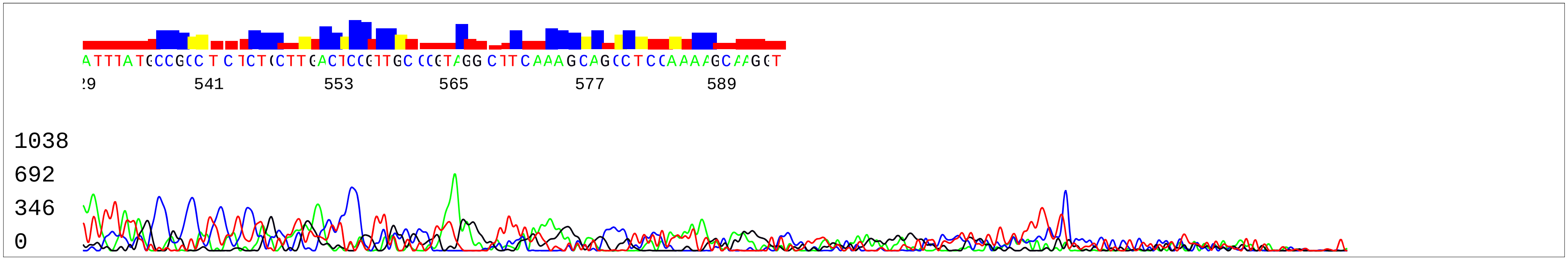

Supplement: Additional file 1: Table S1. — Specimen types and Demographics of E. coli O25b-B2-ST131 isolates. Samples from pus, skin and wound have been illustrated under soft tissue. [file 12866_2014_214_MOESM1_ESM.zip › 12866_2014_214_MOESM1_ESM/12866_2014_214_add31.pdf]
