# Supplementary material for: The characterization and antibiotic resistance profiles of clinical Escherichia coli O25b-B2-ST131 isolates in Kuwait
Source: BMC Microbiol. 2014 Aug 28;14:214. doi: 10.1186/s12866-014-0214-6 (PMC4159528; doi:10.1186/s12866-014-0214-6)

```
  1  GTCAGGGAGA GCTATAGAGC TCCATATCTG GATTATGCGA TGTCGGTCAT TGTTGGCCGT GCGCTGCCAG   70
 71  ATGTCCGAGA TGGCCTGAAG CCGGTACACC GTCGCGTACT TTACGCCATG AACGTACTAG GCAATGACTG  140
141  GAACAAAGCC TATAAAAAAT CTGCCCGTGT CGTTGGTGAC GTAATCGGTA AATACCATCC CCATGGTGAC  210
211  TTGGCGGTTT ATAACACGAT CGTCCGTATG GCGCAGCCAT TCTCGCTGCG TTACATGCTG GTAGACGGTC  280
281  AGGGTAACTT CGGTTCCATC GACGGCGACT CTGCGGCGGC AATGCGTTAT ACGGAAATCC GTCTGGCGAA  350
351  AATTGCCCAT GAACTGATGG CCGATCTCGA AAAAGAGACG GTCGATTTCG TTGATAACTA TGACGGCACG  420
421  GAAAAAATTC CGGACGTCAT GCCAACCAAA ATTCCTAACC TGCTGGTGAA CGGTTCTTCC GGTATCGCCG  490
491  TAGGTATGGC AACCAACATT CCGCCGCACA ACCTGACGGA AGTCATCAAC GGTTGTCTGG CGTATATCGA  560
561  TGATGAAGAC ATCAGCATTG AATTTGCCCC TTTGTTTTAT CCTTTTTCCC CCCAACAACC GTTGATGACT  630
631  TCCGTCAGGT TTGTGCGGCG GAATGTTGGT TGCATACCTA CGGCGATACC GGAAGAACCG TTCACCAGCA  700
701  TGTTAGGAAT TTTGGTTGGA TGACTTCGGA AATTTTTTCG TGCGTCATAG TTTTCAACGA AATCGAACGT  770
771  CTCTTTTTCG AGATCGGCAT CAGTTCATGG GCATTTTCGC GACGGAATTT TCGTATAACG CATTTGCCCC  840
841  CAAATCCCGT CATGGAACCA AGTTCCCTG                                              869
```

Applied Biosystems

3_3130POP7_v3.1_2013-04-25
3
KB_3130_POP7_BDTv3.mob
Pts 1618 to 11071 Pk1 Loc:1595
Version 5.3 HiSQV Bases: 719

Inst Model/Name 3100/3130GeneticAnalyzer-19348-006
Apr 25,2013 01:09PM, AST
Apr 25,2013 01:31PM, AST
Spacing:11.82 Pts/Panel1500
Plate Name: Manar25.04.2013

S/N G:815 A:447 T:434 C:500
KB.bcp
KB 1.4.0 Cap:3

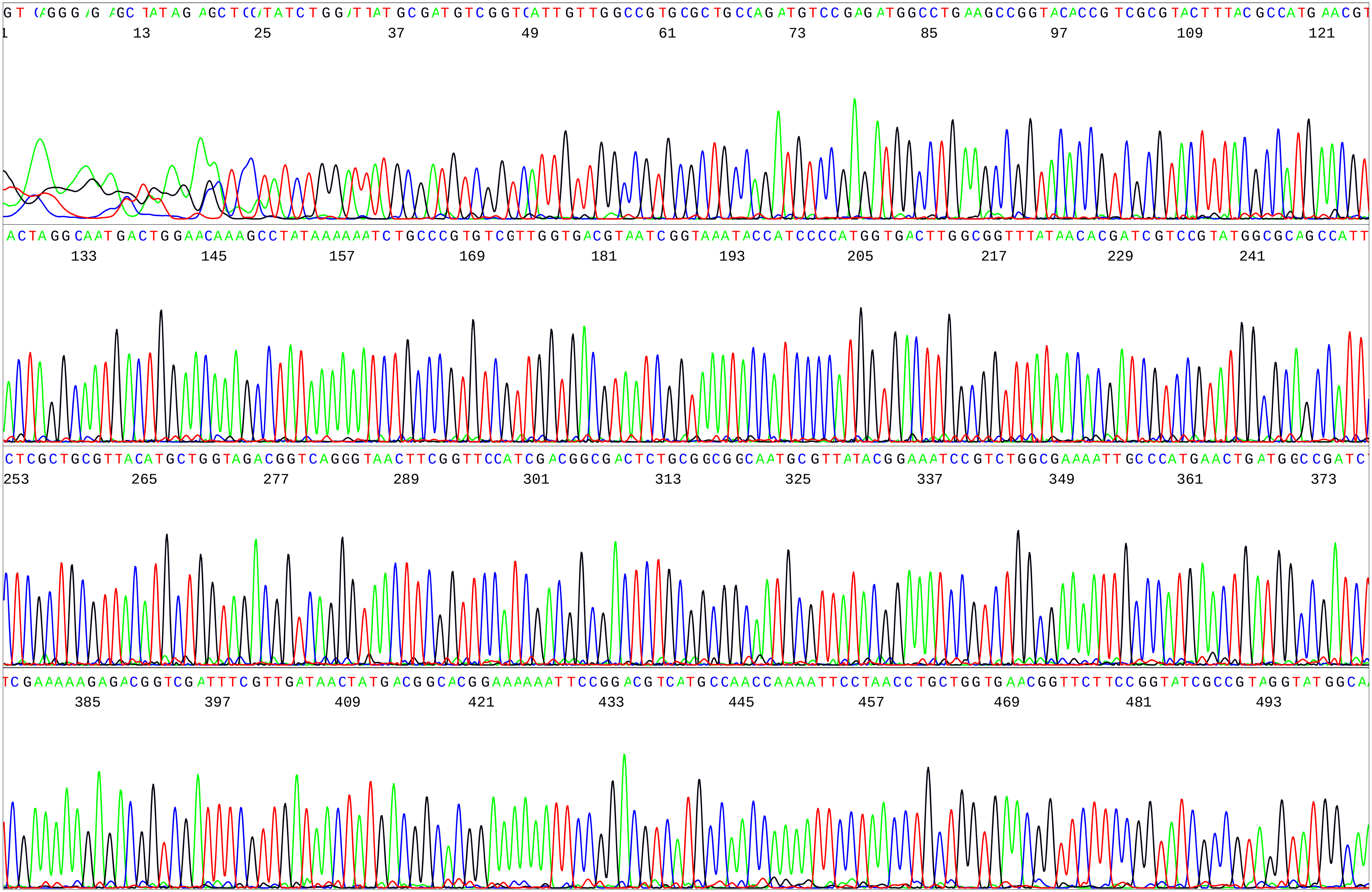

Applied Biosystems

S/N G:815 A:447 T:434 C:500	3_3130POP7_v3.1_2013-04-25	Inst Model/Name 3100/3130GeneticAnalyzer-19348-006
KB.bcp	3	Apr 25,2013 01:09PM, AST
KB 1.4.0 Cap:3	KB_3130_POP7_BDTv3.mob	Apr 25,2013 01:31PM, AST
	Pts 1618 to 11071 Pk1 Loc:1595	Spacing:11.82 Pts/Panel1500
	Version 5.3 HiSQV Bases: 719	Plate Name: Manar25.04.2013

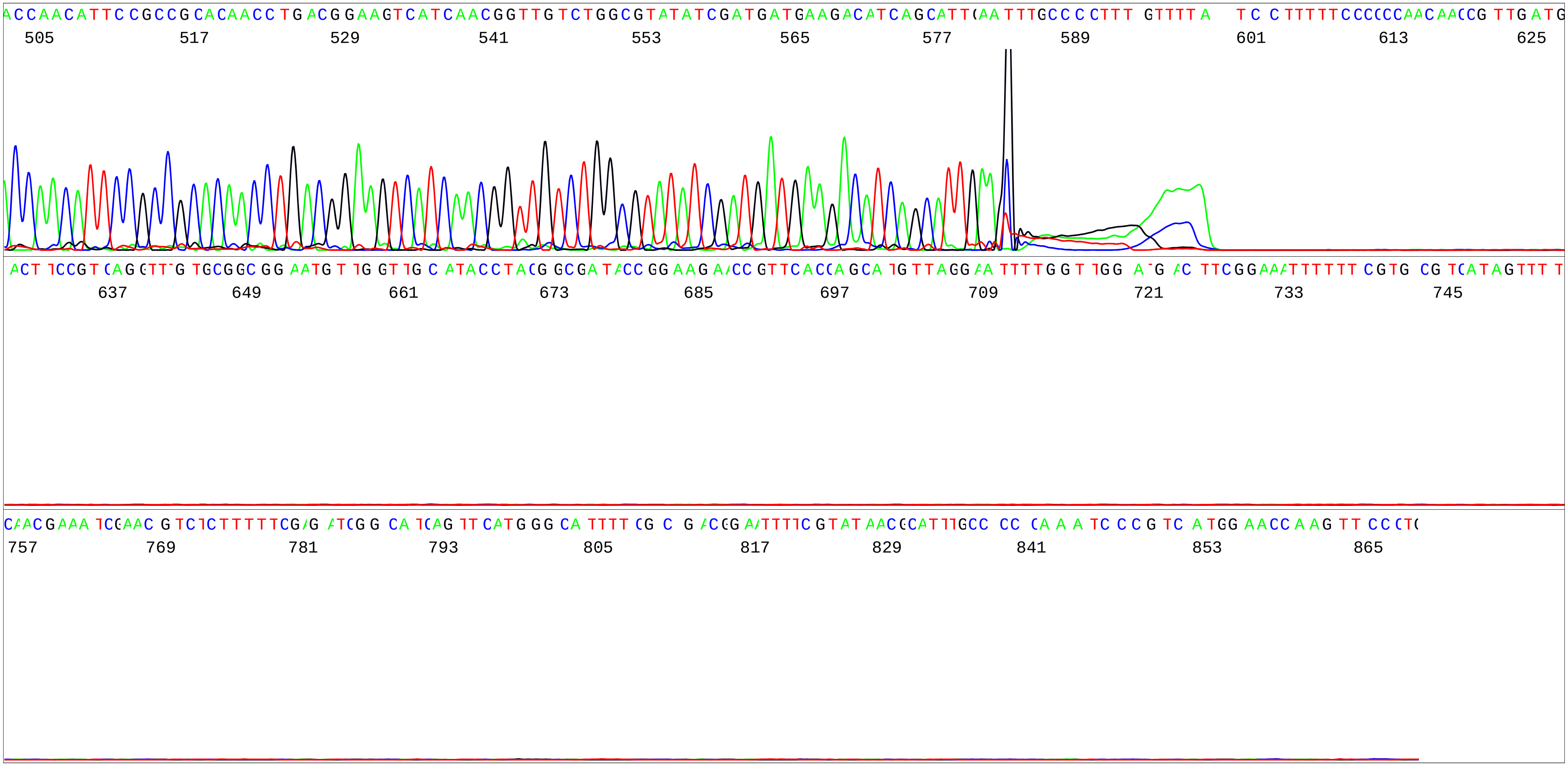

Supplement: Additional file 1: Table S1. — Specimen types and Demographics of E. coli O25b-B2-ST131 isolates. Samples from pus, skin and wound have been illustrated under soft tissue. [file 12866_2014_214_MOESM1_ESM.zip › 12866_2014_214_MOESM1_ESM/12866_2014_214_add32.pdf]
