# Supplementary material for: The characterization and antibiotic resistance profiles of clinical Escherichia coli O25b-B2-ST131 isolates in Kuwait
Source: BMC Microbiol. 2014 Aug 28;14:214. doi: 10.1186/s12866-014-0214-6 (PMC4159528; doi:10.1186/s12866-014-0214-6)

Applied Biosystems

George_03_01_11_2011-01-10_Dashti3GyrF_E07
Dashti3GyrF
KB_3130_POP7_BDTv3.mob
Pts 2469 to 6902 Pk1 Loc:2438
Version 5.3   HiSQV Bases: 312

Inst Model/Name 3100/3130RCF-19348-006
Jan 10,2011 02:31PM, GMT+03:00
Jan 10,2011 02:52PM, GMT+03:00
Spacing:11.0
Plate Name: George_03_01_11

S/N G:2348 A:1808 T:1397 C:1806
KB.bcp
KB 1.4.0   Cap:1

```
  1  ATTGGGGAAC AAAGCCTATA AAAAATCTGC CCGTGTCGTT GGTGACGTAA TCGGTAAATA CCATCCCCAT GGTGACTTGG   80
 81  CGGTTTATAA CACGATCGTC CGTATGGCGC AGCCATTCTC GCTGCGTTAC ATGCTGGTAG ACGGTCAGGG TAACTTCGGT  160
161  TCCATCGACG GCGACTCTGC GGCGGCAATG CGTTATACGG AAATCCGTCT GGCGAAAATT GCCCATGAAC TGATGGCCGA  240
241  TCTCGAAAAA GAGACGGTCG ATTTCGTTGA TAACTATGAC GGCACGGAAA AAATTCCGGA CGTCATGCCA ACCAAAATTC  320
321  CTAACCTGCT GGTGAACGGT TCTTCCGGTA TCGCCGTAGG TATGGGCAAC CAACATTCCG CCGCACAACC TGACGAGGAA  400
401  CAAAGGGAG                                                                                409
```

Applied Biosystems

George_03_01_11_2011-01-10_Dashti3GyrF_E07
Dashti3GyrF
KB_3130_POP7_BDTv3.mob
Pts 2469 to 6902 Pk1 Loc:2438
Version 5.3 HiSQV Bases: 312

Inst Model/Name 3100/3130RCF-19348-006
Jan 10,2011 02:31PM, GMT+03:00
Jan 10,2011 02:52PM, GMT+03:00
Spacing:11.0 Pts/Panel1000
Plate Name: George_03_01_11

S/N G:2348 A:1808 T:1397 C:1806
KB.bcp
KB 1.4.0 Cap:1

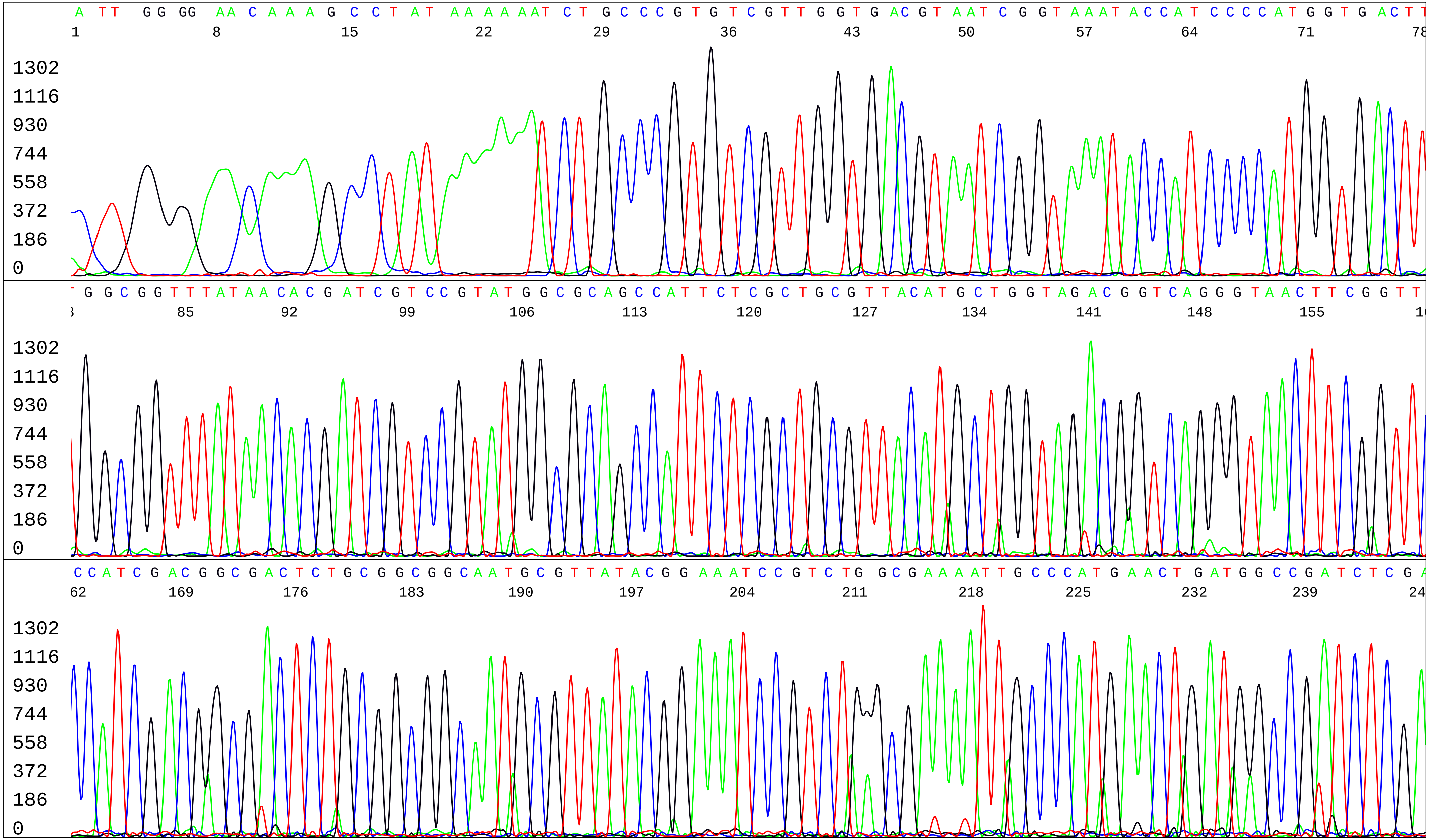

Applied Biosystems

George_03_01_11_2011-01-10_Dashti3GyrF_E07
Dashti3GyrF
KB_3130_POP7_BDTv3.mob
Pts 2469 to 6902 Pk1 Loc:2438
Version 5.3 HiSQV Bases: 312

Inst Model/Name 3100/3130RCF-19348-006
Jan 10,2011 02:31PM, GMT+03:00
Jan 10,2011 02:52PM, GMT+03:00
Spacing:11.0 Pts/Panel1000
Plate Name: George_03_01_11

S/N G:2348 A:1808 T:1397 C:1806
KB.bcp
KB 1.4.0 Cap:1

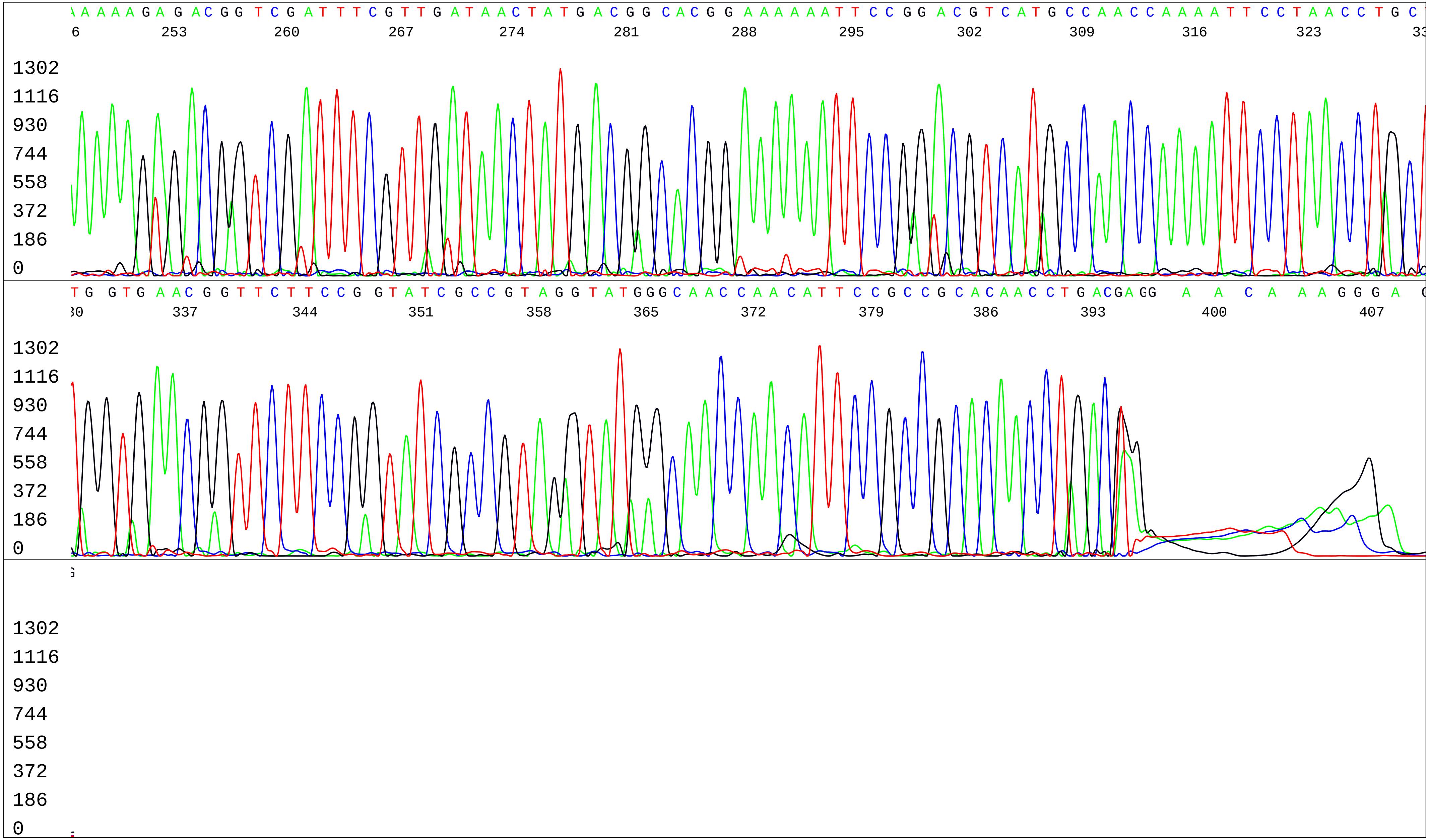

Supplement: Additional file 1: Table S1. — Specimen types and Demographics of E. coli O25b-B2-ST131 isolates. Samples from pus, skin and wound have been illustrated under soft tissue. [file 12866_2014_214_MOESM1_ESM.zip › 12866_2014_214_MOESM1_ESM/12866_2014_214_add33.pdf]
