# Supplementary material for: The characterization and antibiotic resistance profiles of clinical Escherichia coli O25b-B2-ST131 isolates in Kuwait
Source: BMC Microbiol. 2014 Aug 28;14:214. doi: 10.1186/s12866-014-0214-6 (PMC4159528; doi:10.1186/s12866-014-0214-6)

George_03_01_11_2011-01-10_Dashti3GyrR_F07
Dashti3GyrR
KB_3130_POP7_BDTv3.mob
Pts 2210 to 6845 Pk1 Loc:2179
Version 5.3 HiSQV Bases: 389

Inst Model/Name 3100/3130RCF-19348-006
Jan 10,2011 02:31PM, GMT+03:00
Jan 10,2011 02:52PM, GMT+03:00
Spacing:10.9
Plate Name: George_03_01_11

S/N G:2152 A:1537 T:821 C:1251
KB.bcp
KB 1.4.0 Cap:2

```
  1   GATTGGGGTG  CATACCTACG  GCGATACCGG  AGACCGTTCA  CCAGCAGGTT  AGGAATTTTG  GTTGGCATGA  CGTCCGGAAT    80
 81   TTTTTCCGTG  CCGTCATAGT  TATCAACGAA  ATCGACCGTC  TCTTTTTCGA  GATCGGCCAT  CAGTTCATGG  GCAATTTTCG   160
161   CCAGACGGAT  TTCCGTATAA  CGCATTGCCG  CCGCAGAGTC  GCCGTCGATG  GAACCGAAGT  TACCCTGACC  GTCTACCAGC   240
241   ATGTAACGCA  GCGAGAATGG  CTGCGCCATA  CGGACGATCG  TGTTATAAAC  CGCCAAGTCA  CCATGGGGAT  GGTATTTACC   320
321   GATTACGTCA  CCAACGACAC  GGGCAGATTT  TTTATAGGCT  TTGTTCCAGT  CATTGCCTAG  TACGTTCATG  GCGTAAAGTA   400
401   CGCGACGGTG  TACCGGCTTC  AGCCGTTCTC  AAAAAA                                                         436
```

Applied Biosystems

George_03_01_11_2011-01-10_Dashti3GyrR_F07
Dashti3GyrR
KB_3130_POP7_BDTv3.mob
Pts 2210 to 6845 Pk1 Loc:2179
Version 5.3 HiSQV Bases: 389

Inst Model/Name 3100/3130RCF-19348-006
Jan 10,2011 02:31PM, GMT+03:00
Jan 10,2011 02:52PM, GMT+03:00
Spacing:10.9 Pts/Panel1000
Plate Name: George_03_01_11

S/N G:2152 A:1537 T:821 C:1251
KB.bcp
KB 1.4.0 Cap:2

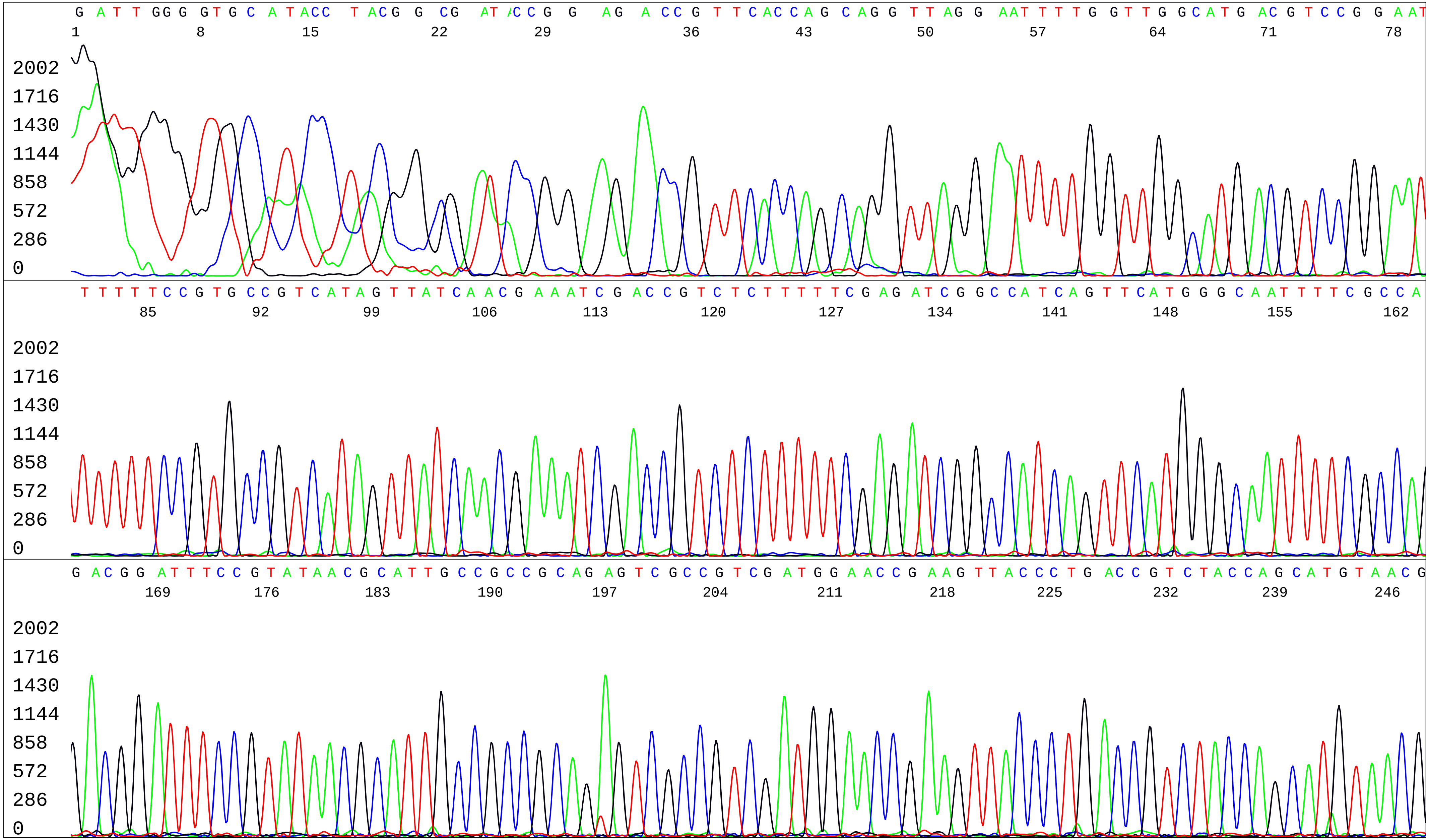

George_03_01_11_2011-01-10_Dashti3GyrR_F07
Dashti3GyrR
KB_3130_POP7_BDTv3.mob
Pts 2210 to 6845 Pk1 Loc:2179
Version 5.3 HiSQV Bases: 389

Inst Model/Name 3100/3130RCF-19348-006
Jan 10,2011 02:31PM, GMT+03:00
Jan 10,2011 02:52PM, GMT+03:00
Spacing:10.9 Pts/Panel1000
Plate Name: George_03_01_11

S/N G:2152 A:1537 T:821 C:1251
KB.bcp
KB 1.4.0 Cap:2

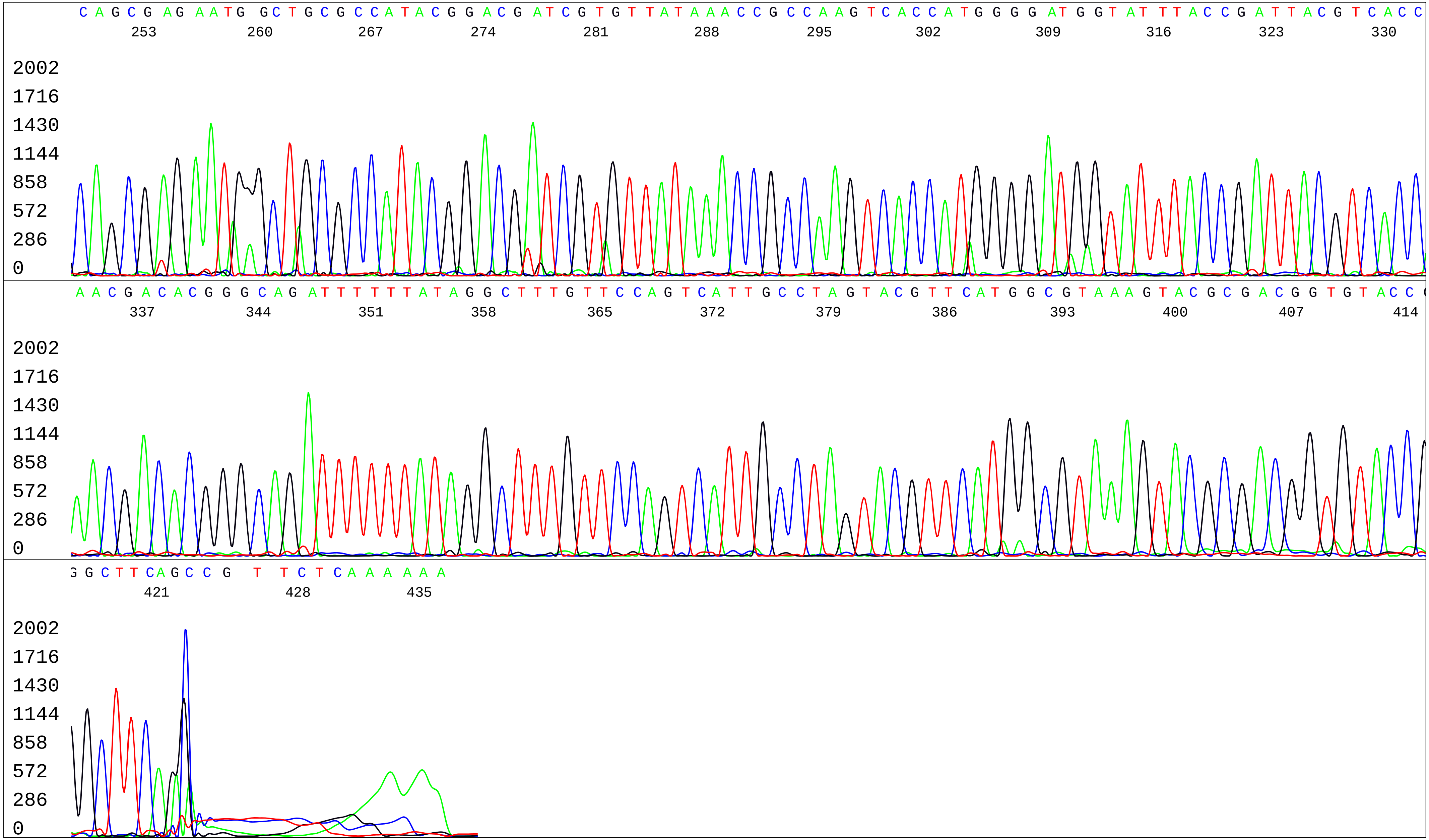

Supplement: Additional file 1: Table S1. — Specimen types and Demographics of E. coli O25b-B2-ST131 isolates. Samples from pus, skin and wound have been illustrated under soft tissue. [file 12866_2014_214_MOESM1_ESM.zip › 12866_2014_214_MOESM1_ESM/12866_2014_214_add34.pdf]
