# Supplementary material for: The characterization and antibiotic resistance profiles of clinical Escherichia coli O25b-B2-ST131 isolates in Kuwait
Source: BMC Microbiol. 2014 Aug 28;14:214. doi: 10.1186/s12866-014-0214-6 (PMC4159528; doi:10.1186/s12866-014-0214-6)

George_03_01_11_2011-01-10_Dashti3PRev_H07
Dashti3PRev
KB_3130_POP7_BDTv3.mob
Pts 2207 to 5963 Pk1 Loc:2176
Version 5.3 HiSQV Bases: 68

Inst Model/Name 3100/3130RCF-19348-006
Jan 10,2011 02:31PM, GMT+03:00
Jan 10,2011 02:52PM, GMT+03:00
Spacing:11.04
Plate Name: George_03_01_11

S/N G:150 A:97 T:48 C:63
KB.bcp
KB 1.4.0 Cap:4

```
  1  TTCCACTCGG GTGAGTCTGA GCTATGCCAG CCACACGGCC ATGTTGCCTG TCTGTCCGAT GGTCCTGATC GCACATCCGT   80
 81  TCTCTTACCC TTATCCGCTG GTTGATGGTC AGGGGAACTG GGGGGCGCCG GACAATACCA AATCGTTCGC GGCCATGTTT  160
161  TACACCAACA TCCCGGTTGT CGAAATATTC CCCCCTGCTA TTGAACCATC TGGGGCAGGG GAAGGCTGAC AGAATGCCTG  240
241  ACTTCTACGG ACCTTTGCAA GATCCCAAAA TGCTGCCTGC CCATCTGCCA AACTTTTTGC TTAACGGCCC CCCCGGTATT  320
321  GCCATATGTA TGGTTACCGA TATTCCTCCG GA                                                      352
```

Applied Biosystems

S/N G:150 A:97 T:48 C:63
KB.bcp
KB 1.4.0 Cap:4

George_03_01_11_2011-01-10_Dashti3PRev_H07
Dashti3PRev
KB_3130_POP7_BDTv3.mob
Pts 2207 to 5963 Pk1 Loc:2176
Version 5.3 HiSQV Bases: 68

Inst Model/Name 3100/3130RCF-19348-006
Jan 10,2011 02:31PM, GMT+03:00
Jan 10,2011 02:52PM, GMT+03:00
Spacing:11.04 Pts/Panel1000
Plate Name: George_03_01_11

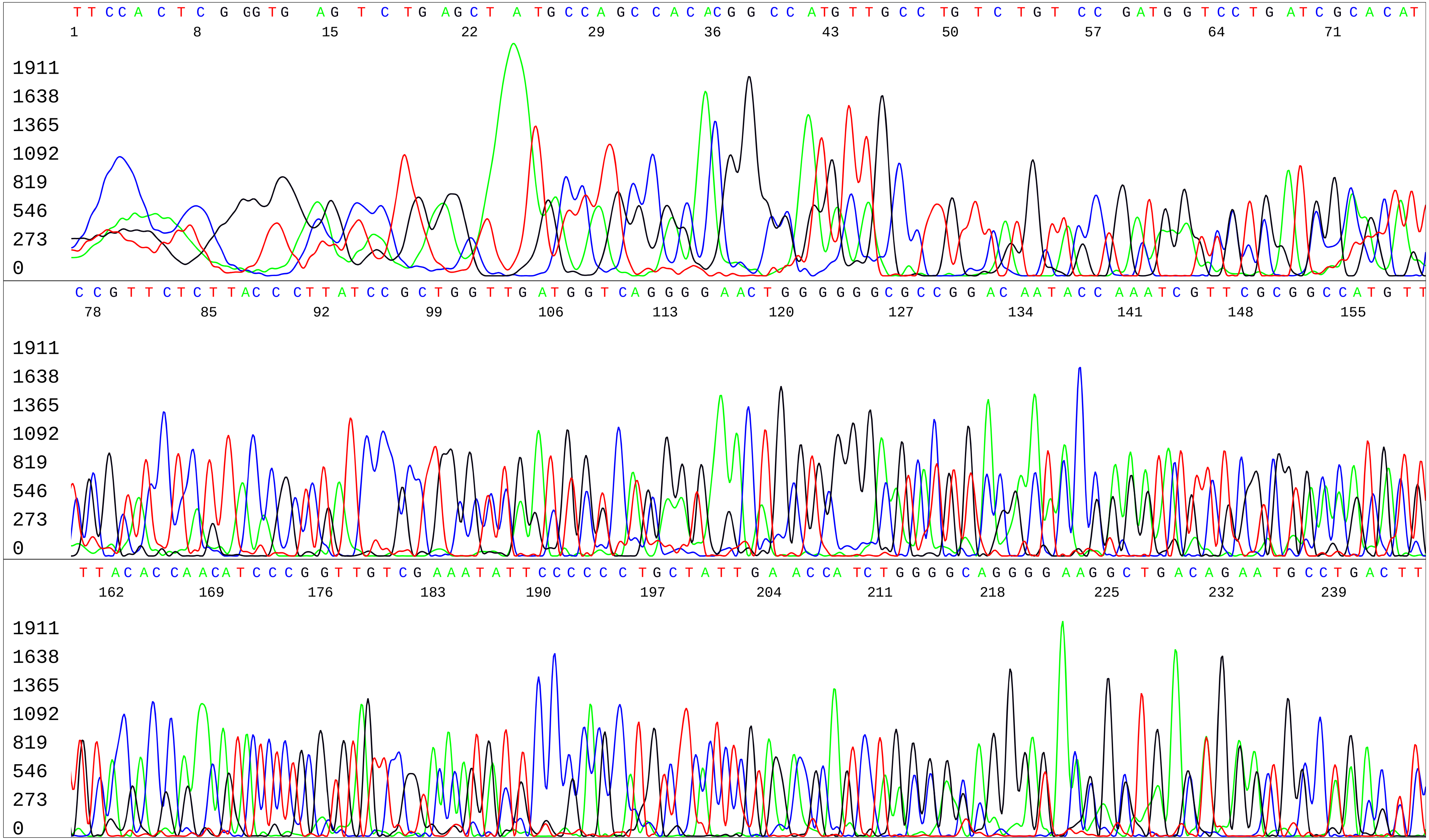

George_03_01_11_2011-01-10_Dashti3PRev_H07
Dashti3PRev
KB_3130_POP7_BDTv3.mob
Pts 2207 to 5963 Pk1 Loc:2176
Version 5.3 HiSQV Bases: 68

Inst Model/Name 3100/3130RCF-19348-006
Jan 10,2011 02:31PM, GMT+03:00
Jan 10,2011 02:52PM, GMT+03:00
Spacing:11.04 Pts/Panel1000
Plate Name: George_03_01_11

S/N G:150 A:97 T:48 C:63
KB.bcp
KB 1.4.0 Cap:4

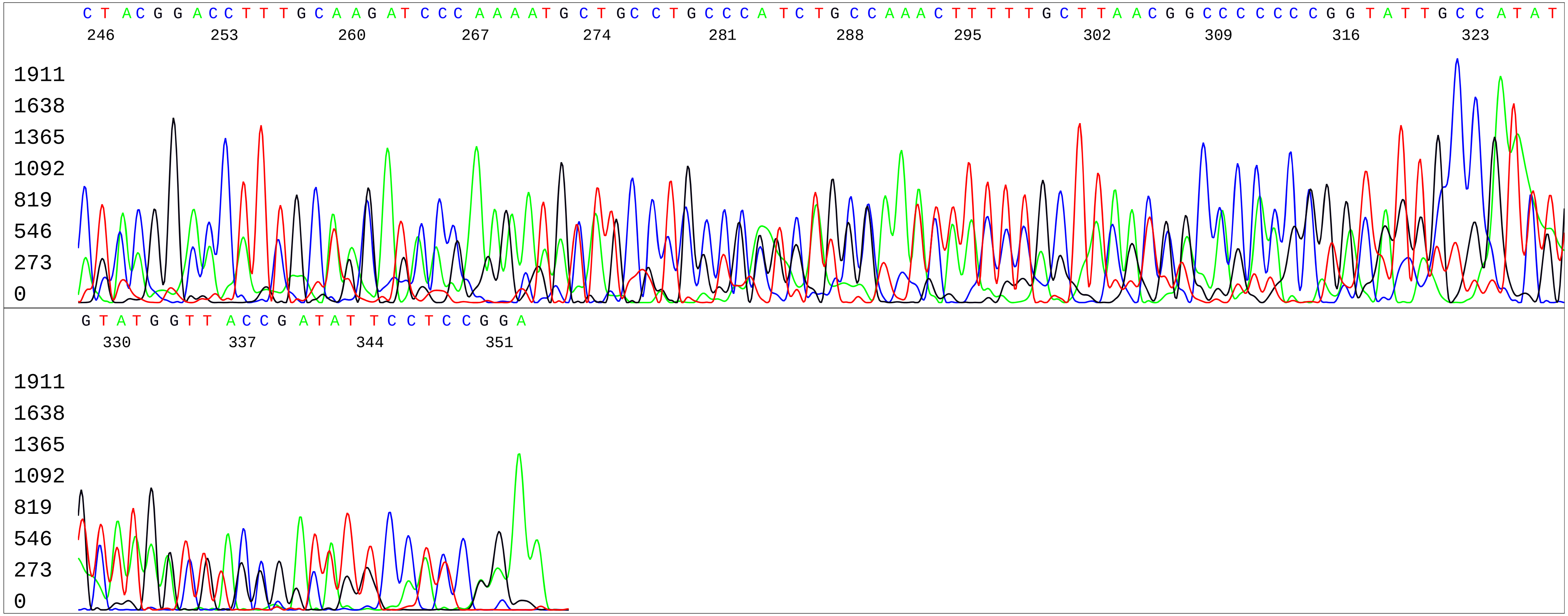

Supplement: Additional file 1: Table S1. — Specimen types and Demographics of E. coli O25b-B2-ST131 isolates. Samples from pus, skin and wound have been illustrated under soft tissue. [file 12866_2014_214_MOESM1_ESM.zip › 12866_2014_214_MOESM1_ESM/12866_2014_214_add35.pdf]
