# Supplementary material for: The characterization and antibiotic resistance profiles of clinical Escherichia coli O25b-B2-ST131 isolates in Kuwait
Source: BMC Microbiol. 2014 Aug 28;14:214. doi: 10.1186/s12866-014-0214-6 (PMC4159528; doi:10.1186/s12866-014-0214-6)
Supplement: Additional file 1: Table S1. — Specimen types and Demographics of E. coli O25b-B2-ST131 isolates. Samples from pus, skin and wound have been illustrated under soft tissue. [file 12866_2014_214_MOESM1_ESM.zip › 12866_2014_214_MOESM1_ESM/12866_2014_214_add37.docx]

sample sequenced was KOC (E.Coli) for CIT (AmpC) forward and reverse primers used.

Results:

**Forward**

NNNNNNNNNNNCNNNNTGCNNTTNNCCNCCTATACGGCAGGCGNCCTACNCGCTGCAGAT

CNCCCNATNACNNNNAGGGATAAAGCCGCATTACTGCATTTTTATCAAAACTGGCAGCCG

CAATGGACTCCGGGCGCTAAGCGACTTTACGCTAACTCCAGCATTGGTCTGTTTGGCGCG

CTGGCGGTGAAACCCTCAGGAATGAGTTACGAAGAGGCAATGACCAGACGCGTCCTGCAA

CCATTAAAACTGGCGCATACCTGGATTACGGTTCCGCAGAACGAACAAAAAGATTATGCC

TGGGGCTATCGCGAAGGGAAGCCCGTACACGTTTCTCCGGGACAACTTGACGCCGAAGCC

TATGGCGTGAAATCCAGCGTTATTGATATGGCCCGCTGGGTTCAGGCCAACATGGATGCC

AGCCACGTTCAGGAGAAAAANGATNNCNANNGNANAATCTTTTTGTTCGCTCGGCGGANN

NGGAATCGANGTATGANTAGNTNTAAAGGTTGGTGGGAGCGTCAGGGCANGCCTGTNCGT

AAAATTGTCCATAGGGATTCATCNCNAGNNNNCNNAAAAACCAATGCTGGAANTAGCGTA

AAGTCGCTTANCGCCCGGNAGTCNATTGNGGGCTGCCAGTTTTGATAAAANTGNNNNAAT

GGCGGCTTTATCCCTAACGTCATCGGGGATCTGCAGCGGNNNGGNCNNCNGNCNNATANG

GTGGNTTAATGCAGNNNGNGGANNNCCCNGCCACTGTTTGCCTGTCAGTTCTGGGNCAAA

NA

**Reverse**

NNNNNNNNNNNNNGGNNNNNNNNNTNNNNNCNCTGGATTNCCGCNNTAGGCTGCGGCGTC

AAGTTGCCCAGAAAAAACGTGTACGGCCTTCCCTTCGCGATACCCCCGGGCATAATCTTT

TTGTTCGTTCTGCGAAACCGAAATCCAGGTATGCCCCATTTTTAATGGTTGCAGGACGCG

TCTGGTCATTGCCTCTTCGTAACTCATTCCTGAGGGTTTCCCCGCCGGCGCGCCAAACAA

ACCAATGCTGGATTTAGCGAAAAGTCGTTTAGCCCCCGGAGTCCATTGCGGCTGCCATTT

TTGATAAAAATGCAGTAATGCGGTTTTATCCCTAACGTCATCGGGAATCTGCAGCGGTAG

GCCGCCTGCCGTATAGGTGGCTAAGTGCAGCAGGCGGATCCCCTGCCACTGTTTGCCTGT

CAGTTCTGGCAAANNNNNNNNNANTACTTTACNNGATTATTAACCGNNNGTAGGTACNGG

TATGGAAACGTANCTTGCGCGTGACTCNTGTGTGTGTGTNANCGCANACCGTGGNTATGT

AATGATTATGTNGATNCTTCNNAACCCCCNGANNNAA
