# Supplementary material for: The characterization and antibiotic resistance profiles of clinical Escherichia coli O25b-B2-ST131 isolates in Kuwait
Source: BMC Microbiol. 2014 Aug 28;14:214. doi: 10.1186/s12866-014-0214-6 (PMC4159528; doi:10.1186/s12866-014-0214-6)

Applied Biosystems

S/N G:671 A:364 T:219 C:264
KB.bcp
KB 1.4.0 Cap:4

C1F_3130POP7_v3.1_2013-05-21
C1F
KB_3130_POP7_BDTv3.mob
Pts 1941 to 11071 Pk1 Loc:1918
Version 5.3 HiSQV Bases: 710

Inst Model/Name 3100/3130GeneticAnalyzer-19348-006
May 21,2013 04:48PM, AST
May 21,2013 05:10PM, AST
Spacing:12.94
Plate Name: 21.05.2013

```
  1   GATCCTGCGC AGTTCACGCT GATGGCGACG GCAACCGTCA CGCTGTTGTT AGGAAGTGTG CCGCTGTATG    70
 71   CGCAAACGGC GGACGTACAG CAAAAACTTG CCGAATTAGA GCGGCAGTCG GGAGGCAGAC TGGGTGTGGC   140
141   ATTGATTAAC ACAGCAGATA ATTCGCAAAT ACTTTATCGT GCTGATGAGC GCTTTGCGAT GTGCAGCACC   210
211   AGTAAAGTGA TGGCCGCGGC CGCGGTGCTG AAGAAAAGTG AAAGCGAACC GAATCTGTTA AATCAGCGAG   280
281   TTGAGATCAA AAAATCTGAC CTTGTTAACT ATAATCCGAT TGCGGAAAAG CACGTCAATG GGACGATGTC   350
351   ACTGGCTGAG CTTAGCGCGG CCGCGCTACA GTACAGCGAT AACGTGGCGA TGAATAAGCT GATTGCTCAC   420
421   GTTGGCGGCC CGGCTAGCGT CACCGCGTTC GCCCGACAGC TGGGAGACGA AACGTTCCGT CTCGACCGTA   490
491   CCGAGCCGAC GTTAAACACC GCCATTCCGG GCGATCCGCG TGATACCACT TCACCTCGGG CAATGGCGCA   560
561   AACTCTGCGG AATCTGACGC TGGGTAAAGC ATTGGGCGAC AGCCAACGGG CGCAGCTGGT GACATGGATG   630
631   AAAGGCAATA CCACCGGTGC AGCGAGCATT CAGGCTGGAC TGCCTGCTTC CTGGGTTGTG GGGGATAAAA   700
701   CCGGCAGCGG TGGCTATGGC ACCACCAACG AT                                            732
```

Applied Biosystems

S/N G:671 A:364 T:219 C:264
KB.bcp
KB 1.4.0 Cap:4

C1F_3130POP7_v3.1_2013-05-21
C1F
KB_3130_POP7_BDTv3.mob
Pts 1941 to 11071 Pk1 Loc:1918
Version 5.3 HiSQV Bases: 710

Inst Model/Name 3100/3130GeneticAnalyzer-19348-006
May 21,2013 04:48PM, AST
May 21,2013 05:10PM, AST
Spacing:12.94 Pts/Panel1500
Plate Name: 21.05.2013

G ATCC 1G CG CAG TTCACGCTG ATGGCGACGGCAACCGTCACGCTGTTGTTAGGAAGTGTGCCGCTGTATGCGCAAACGGCGGACGTACAGCAAAAACTTGCCGAATTAGAGCGGCAGTCG
1 12 23 34 45 56 67 78 89 100 111

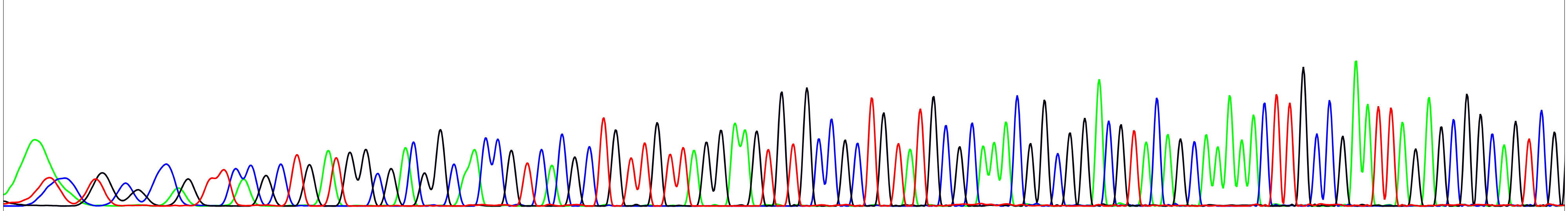

GGAGGCAGACTGGGTGTGGCATTGATTAACACAGCAGATAATTCGCAAATACTTTATCGTGCTGATGAGCGCTTTGCGATGTGCAGCACCAGTAAAGTGATGGCCGCGGCCGCGGTGCTGAAGAAA
122 133 144 155 166 177 188 199 210 221 232 243

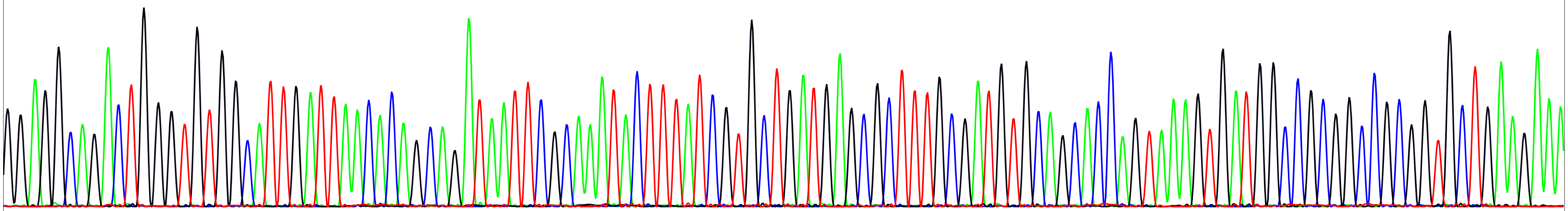

AAGTGAAAGCGAACCGAATCTGTTAAATCAGCGAGTTGAGATCAAAAAATCTGACCTTGTTAACTATAATCCGATTGCGGAAAAGCACGTCAATGGGACGATGTCACTGGCTGAGCTTAGCGCGGCC
254 265 276 287 298 309 320 331 342 353 364

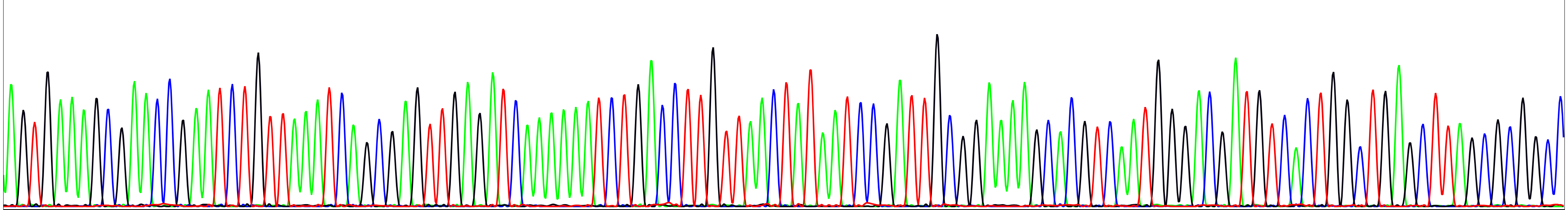

CGCGCTACAGTACAGCGATAACGTGGCGATGAATAAGCTGATTGCTCACGTTGGCGGCCCGGCTAGCGTCACCGCGTTCGCCCGACAGCTGGGAGACGAAACGTTCCGTCTCGACCGTACCGAGCC
375 386 397 408 419 430 441 452 463 474 485 496

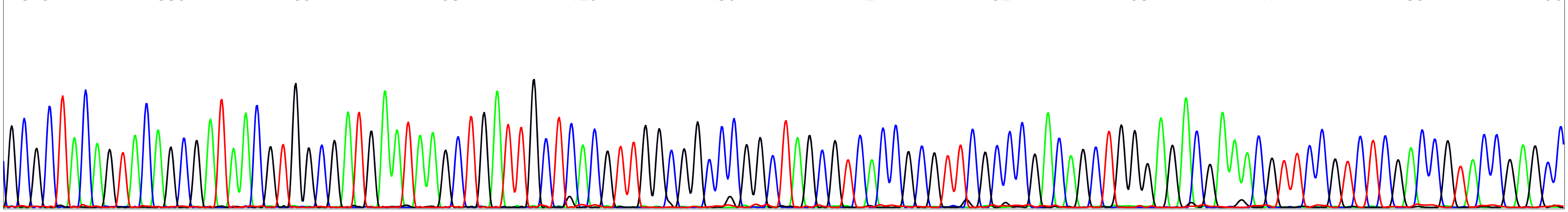

Applied Biosystems

C1F_3130POP7_v3.1_2013-05-21
C1F
KB_3130_POP7_BDTv3.mob
Pts 1941 to 11071 Pk1 Loc:1918
Version 5.3 HiSQV Bases: 710

Inst Model/Name 3100/3130GeneticAnalyzer-19348-006
May 21,2013 04:48PM, AST
May 21,2013 05:10PM, AST
Spacing:12.94 Pts/Panel1500
Plate Name: 21.05.2013

S/N G:671 A:364 T:219 C:264
KB.bcp
KB 1.4.0 Cap:4

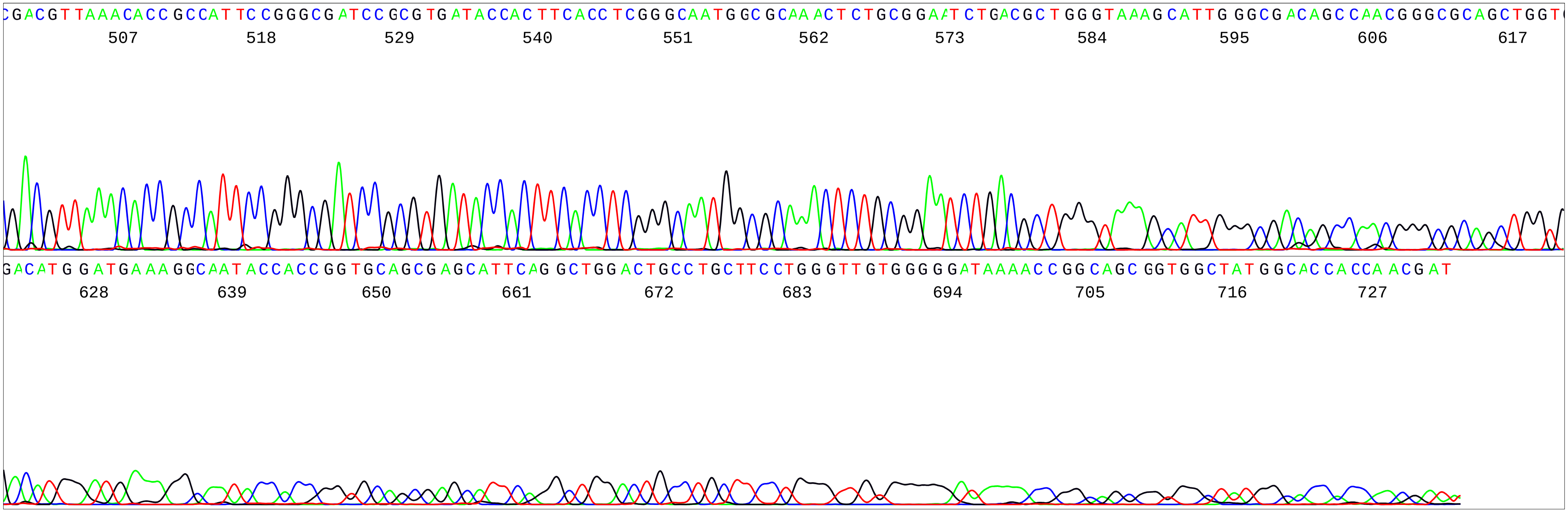

Supplement: Additional file 1: Table S1. — Specimen types and Demographics of E. coli O25b-B2-ST131 isolates. Samples from pus, skin and wound have been illustrated under soft tissue. [file 12866_2014_214_MOESM1_ESM.zip › 12866_2014_214_MOESM1_ESM/12866_2014_214_add5.pdf]
