# Supplementary material for: The characterization and antibiotic resistance profiles of clinical Escherichia coli O25b-B2-ST131 isolates in Kuwait
Source: BMC Microbiol. 2014 Aug 28;14:214. doi: 10.1186/s12866-014-0214-6 (PMC4159528; doi:10.1186/s12866-014-0214-6)

Applied Biosystems

C1R_3130POP7_v3.1_2013-05-21
C1R
KB_3130_POP7_BDTv3.mob
Pts 2270 to 11071 Pk1 Loc:2247
Version 5.3   HiSQV Bases: 629

Inst Model/Name 3100/3130GeneticAnalyzer-19348-006
May 21,2013 04:48PM, AST
May 21,2013 05:10PM, AST
Spacing:13.11
Plate Name: 21.05.2013

S/N G:110 A:46 T:43 C:55
KB.bcp
KB 1.4.0   Cap:3

1	CTGCCCTTAG	GTTGAGGCTG	GGTGAAGTAA	GTGACCAGAA	TCAGCGGCGC	ACGATCTTTT	GGCCAGATCA	70
71	CCGCGATATC	GTTGGTGGTG	CCATAGCCAC	CGCTGCCGGT	TTTATCCCCC	ACAACCCAGG	AAGCAGGCAG	140
141	TCCAGCCTGA	ATGCTCGCTG	CACCGGTGGT	ATTGCCTTTC	ATCCATGTCA	CCAGCTGCGC	CCGTTGGCTG	210
211	TCGCCCAATG	CTTTACCCAG	CGTCAGATTC	CGCAGAGTTT	GCGCCATTGC	CCGAGGTGAA	GTGGTATCAC	280
281	GCGGATCGCC	CGGAATGGCG	GTGTTTAACG	TCGGCTCGGT	ACGGTCGAGA	CGGAACGTTT	CGTCTCCCAG	350
351	CTGTCGGGCG	AACGCGGTGA	CGCTAGCCGG	GCCGCCAACG	TGAGCAATCA	GCTTATTCAT	CGCCACGTTA	420
421	TCGCTGTACT	GTAGCGCGGC	CGCGCTAAGC	TCAGCCAGTG	ACATCGTCCC	ATTGACGTGC	TTTTCCGCAA	490
491	TCGGATTATA	GTTAACAAGG	TCAGATTTTT	TGATCTCAAC	TCGCTGATTT	AACAGATTCG	GTTCGCTTTC	560
561	ACTTTTCTTC	AGCACCGCGG	CCGCGGCCAT	CACTTTACTG	GTGCTGCACA	TCGCAAAGCG	CTCATCAGCA	630
631	CGATAAAGTA	TTTGCGAATT	ATCTGCTGTG	TTAATCAATG	CCACACCCAG	TCTGCCTCCC	GACTGCCGCT	700

Applied Biosystems

S/N G:110 A:46 T:43 C:55
KB.bcp
KB 1.4.0 Cap:3

C1R_3130POP7_v3.1_2013-05-21
C1R
KB_3130_POP7_BDTv3.mob
Pts 2270 to 11071 Pk1 Loc:2247
Version 5.3 HiSQV Bases: 629

Inst Model/Name 3100/3130GeneticAnalyzer-19348-006
May 21,2013 04:48PM, AST
May 21,2013 05:10PM, AST
Spacing:13.11 Pts/Panel1500
Plate Name: 21.05.2013

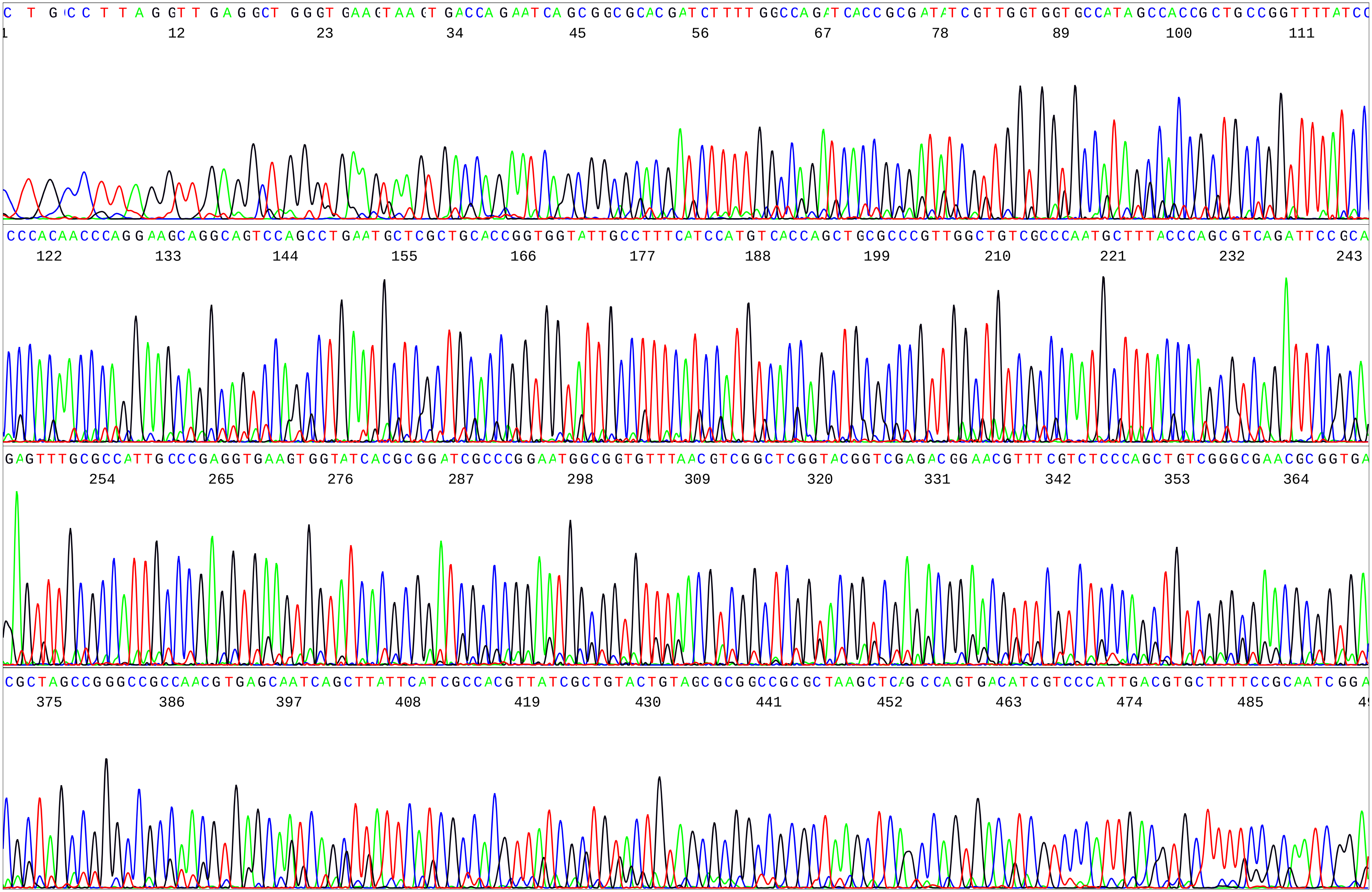

C1R_3130POP7_v3.1_2013-05-21
C1R
KB_3130_POP7_BDTv3.mob
Pts 2270 to 11071 Pk1 Loc:2247
Version 5.3   HiSQV Bases: 629

Inst Model/Name 3100/3130GeneticAnalyzer-19348-006
May 21,2013 04:48PM, AST
May 21,2013 05:10PM, AST
Spacing:13.11 Pts/Panel1500
Plate Name: 21.05.2013

S/N G:110 A:46 T:43 C:55
KB.bcp
KB 1.4.0   Cap:3

TTATAGTTAACAAGGTCAGATTTTTTGATCTCAACTCGCTGATTTAACAGATTCGGTTCGCTTTCACTTTTCTTCAGCACCGCGGCCGCGGCCATCACTTTACTGGTGCTGCACATCGCAAA

96 507 518 529 540 551 562 573 584 595 606 617

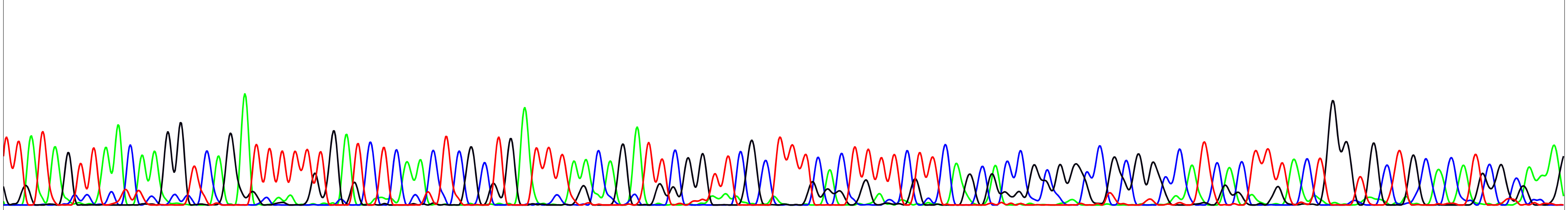

GCGCTCATCAGCACGATAAAGTATTTGCGAATTATCTGCTGTGTTAATCAATGCCACACCCAGTCTGCCTCCCGACTGCCGCT

7 628 639 650 661 672 683 694

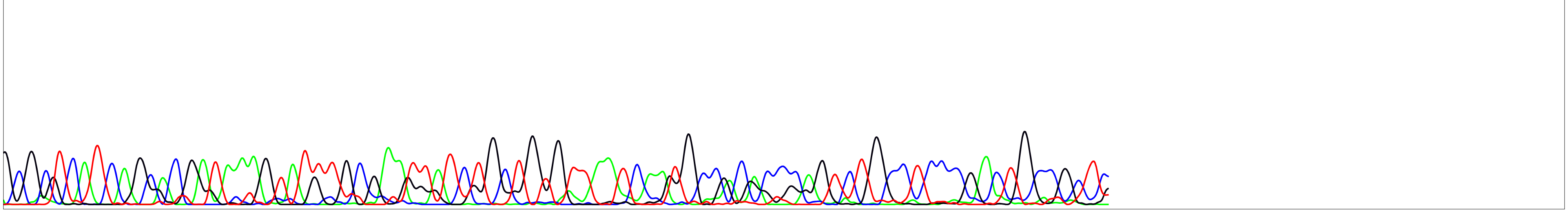

Supplement: Additional file 1: Table S1. — Specimen types and Demographics of E. coli O25b-B2-ST131 isolates. Samples from pus, skin and wound have been illustrated under soft tissue. [file 12866_2014_214_MOESM1_ESM.zip › 12866_2014_214_MOESM1_ESM/12866_2014_214_add6.pdf]
