# Supplementary material for: The characterization and antibiotic resistance profiles of clinical Escherichia coli O25b-B2-ST131 isolates in Kuwait
Source: BMC Microbiol. 2014 Aug 28;14:214. doi: 10.1186/s12866-014-0214-6 (PMC4159528; doi:10.1186/s12866-014-0214-6)
Supplement: Additional file 1: Table S1. — Specimen types and Demographics of E. coli O25b-B2-ST131 isolates. Samples from pus, skin and wound have been illustrated under soft tissue. [file 12866_2014_214_MOESM1_ESM.zip › 12866_2014_214_MOESM1_ESM/12866_2014_214_add7.pdf]

```
1     TCGGACTGCG AATCATGGGT AGTGGGCGAT AAAACCGGCA GCGGAGATTA TGGCACCACC AACGATATCG    70
71    CGGTTATCTG GCCGGAAAAC CACGCACCGC TGGTTCTGGT GACCTACTTT ACCCAACCGG AGCAGAAGGC   140
141   GGAAAGCCGT CGGGATATTC TGGCTGCGGC GGCGAAAATC GTAACCCACG GTTTCTGATG CAATAATCTA   210
211   GAG                                                                            213
```

Applied Biosystems

S/N G:333 A:185 T:130 C:161
KB.bcp
KB 1.4.0   Cap:3

S7_3130POP7_v3.1_2014-03-11
S7
KB_3130_POP7_BDTv3.mob
Pts 1829 to 11071 Pk1 Loc:1806
Version 5.3   HiSQV Bases: 192

Inst Model/Name 3100/3130GeneticAnalyzer-19348-006
Mar 11,2014 11:26PM, AST
Mar 11,2014 11:48PM, AST
Spacing:-13.4 Pts/Panel1500
Plate Name: Febine

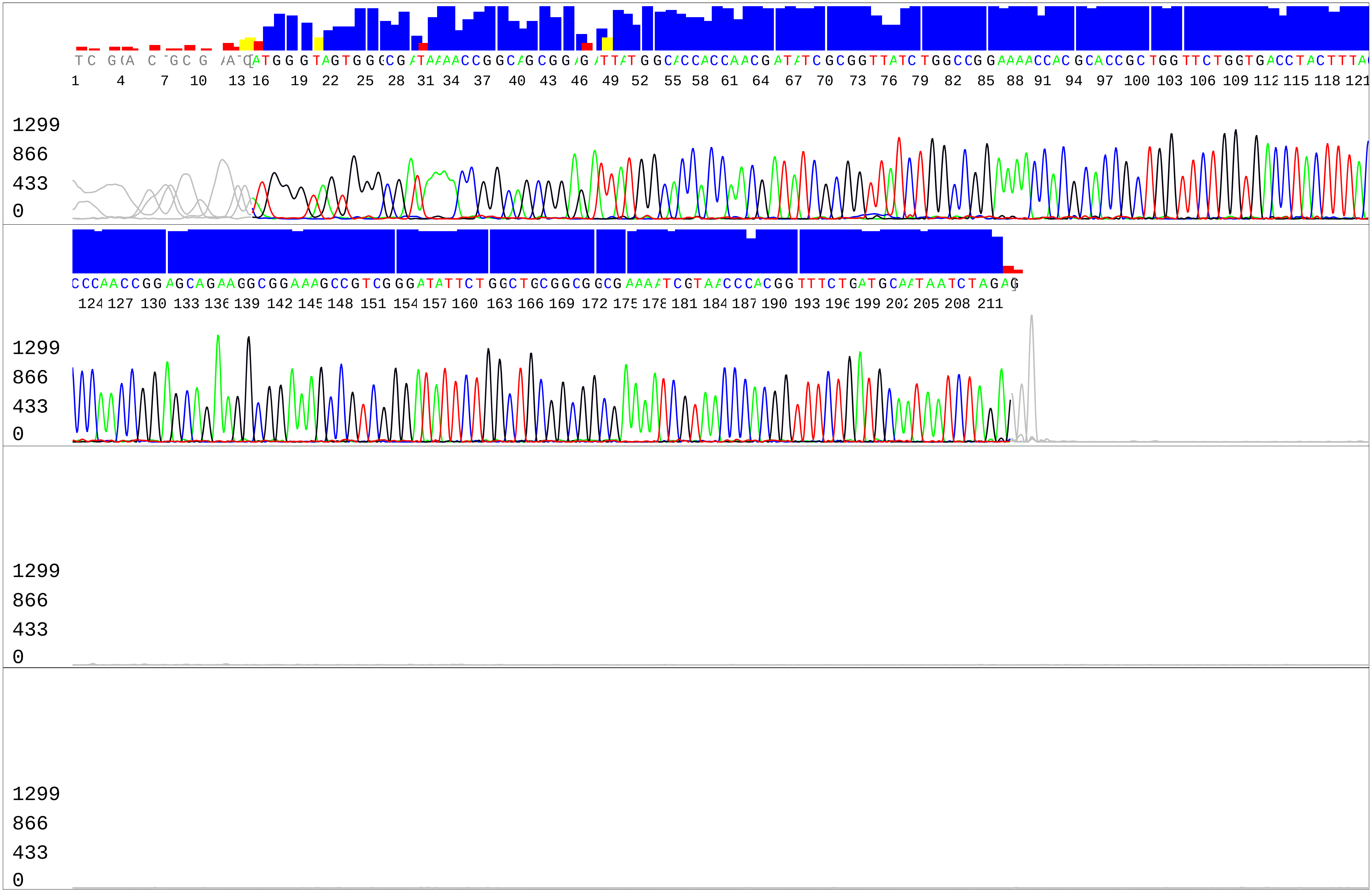

Printed on: Wed Mar 12,2014 09:06AM, AST

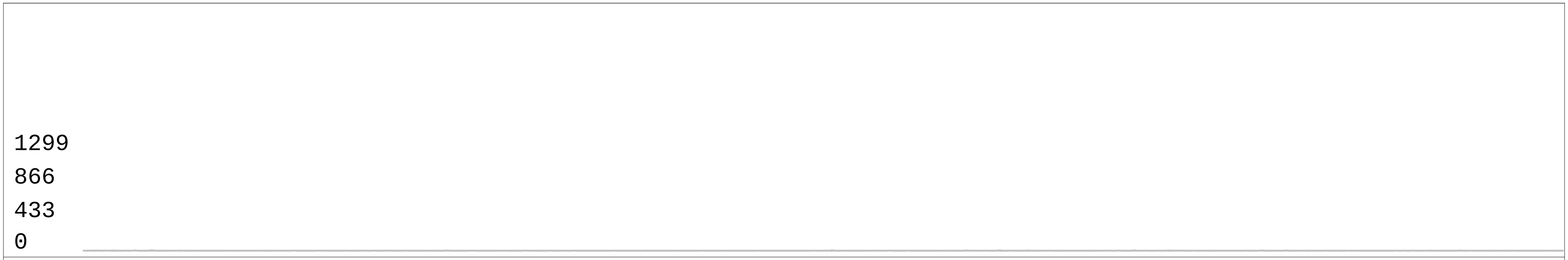
1299
866
433
0

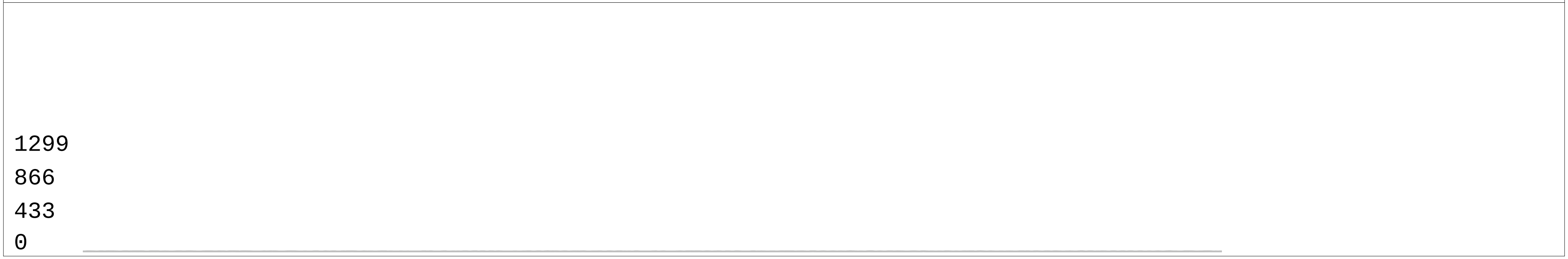
1299
866
433
0
