# Supplementary material for: The characterization and antibiotic resistance profiles of clinical Escherichia coli O25b-B2-ST131 isolates in Kuwait
Source: BMC Microbiol. 2014 Aug 28;14:214. doi: 10.1186/s12866-014-0214-6 (PMC4159528; doi:10.1186/s12866-014-0214-6)

```
  1  GCCTAATGTT TACGGTGATG GCGACGCTAC CCCTGCTATT TAGCAGCGCA ACGCTGCATG CGCAGGCGAA   70
 71  CAGCGTGCAA CAGCAGCTGG AAGCCCTGGA GAAAAGTTCG GGAGGTCGGC TTGGCGTTGC GCTGATTAAC  140
141  ACCGCCGATA ATTCGCAGAT TCTCTACCGT GCCGATGAAC GTTTTGCGAT GTGCAGTACC AGTAAGGTGA  210
211  TGGCGGCCGC GGCGGTGCTT AAACAGAGCG AGAGCGATAA GCACCTGCTA AATCAGCGCG TTGAAATCAA  280
281  GAAGAGCGAC CTGGTTAACT ACAATCCCAT TGCGGAGAAA CACGTTAACG GCACGATGAC GCTGGCTGAG  350
351  CTTGGCGCAG CGGCGCTGCA GTATAGCGAC AATACTGCCA TGAATAAGCT GATTGCCCAT CTGGGTGGTC  420
421  CCGATAAAGT GACGGCGTTT GCTCGCTCGT TGGGTGATGA GACCTTCCGT CTGGACAGAA CCGAGCCCAC  490
491  GCTCAATACC GCCATTCCAG GCGACCCGCG TGATACCACC ACGCCGCTCG CGATGGCGCA GACCCTGAAA  560
561  AATCTGACGC TGGGTAAAGC GCTGGCGGAA ACTCAGCGGG CACAGTTGGT GACGTGGCTT AAGGGCAATA  630
631  CTACCGGTAG CGCGAGCATT CGGGCGGGTC TGCCGAAATC ATGGGTAGTG GGCGATAAAA CCGGCAGCGG  700
701  ACATTATGGC ACCACCAACG ATATCGCGGT                                              730
```

Applied Biosystems

S/N G:97 A:52 T:32 C:40
KB.bcp
KB 1.4.0 Cap:1

S5_3130POP7_v3.1_2014-03-11
S5
KB_3130_POP7_BDTv3.mob
Pts 1835 to 11071 Pk1 Loc:1812
Version 5.3 HiSQV Bases: 701

Inst Model/Name 3100/3130GeneticAnalyzer-19348-006
Mar 11,2014 11:26PM, AST
Mar 11,2014 11:48PM, AST
Spacing:13.27 Pts/Panel1500
Plate Name: Febine

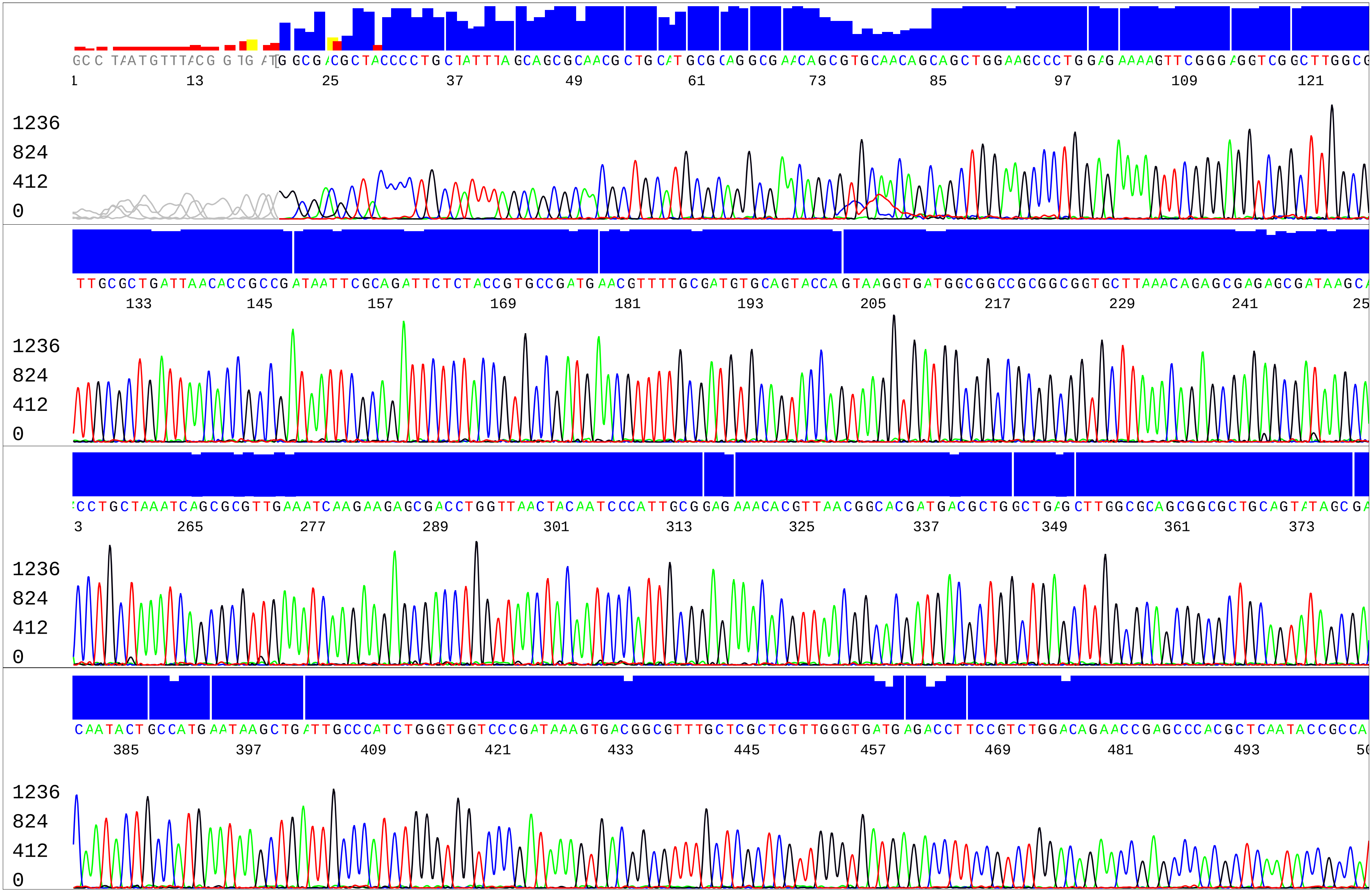

S5_3130POP7_v3.1_2014-03-11
S5
KB_3130_POP7_BDTv3.mob
Pts 1835 to 11071 Pk1 Loc:1812
Version 5.3   HiSQV Bases: 701

Inst Model/Name 3100/3130GeneticAnalyzer-19348-006
Mar 11,2014 11:26PM, AST
Mar 11,2014 11:48PM, AST
Spacing:13.27 Pts/Panel1500
Plate Name: Febine

S/N G:97 A:52 T:32 C:40
KB.bcp
KB 1.4.0   Cap:1

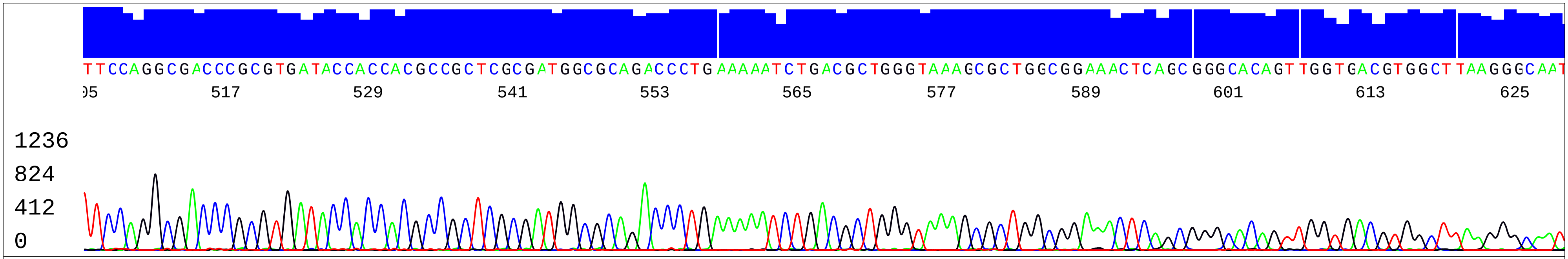

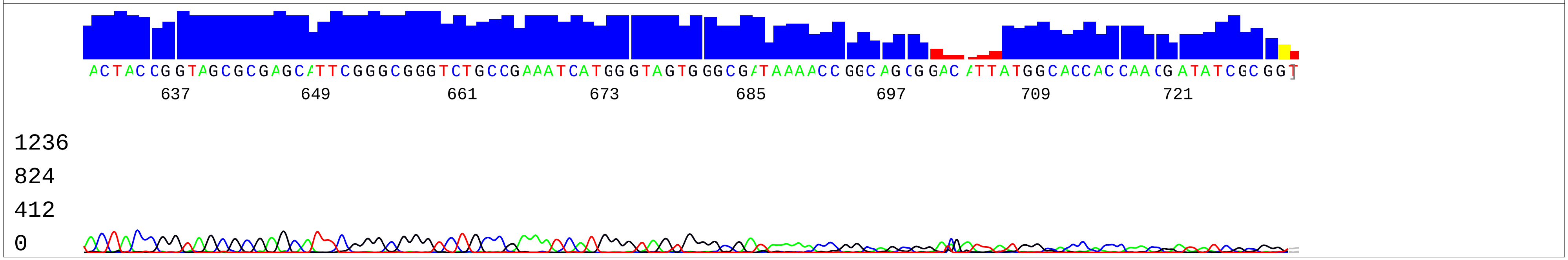

Supplement: Additional file 1: Table S1. — Specimen types and Demographics of E. coli O25b-B2-ST131 isolates. Samples from pus, skin and wound have been illustrated under soft tissue. [file 12866_2014_214_MOESM1_ESM.zip › 12866_2014_214_MOESM1_ESM/12866_2014_214_add8.pdf]
