# Supplementary material for: The characterization and antibiotic resistance profiles of clinical Escherichia coli O25b-B2-ST131 isolates in Kuwait
Source: BMC Microbiol. 2014 Aug 28;14:214. doi: 10.1186/s12866-014-0214-6 (PMC4159528; doi:10.1186/s12866-014-0214-6)

Applied Biosystems

S/N G:84 A:37 T:36 C:41
KB.bcp
KB 1.4.0 Cap:2

S6_3130POP7_v3.1_2014-03-11
S6
KB_3130_POP7_BDTv3.mob
Pts 1840 to 11071 Pk1 Loc:1817
Version 5.3 HiSQV Bases: 696

Inst Model/Name 3100/3130GeneticAnalyzer-19348-006
Mar 11,2014 11:26PM, AST
Mar 11,2014 11:48PM, AST
Spacing:13.42
Plate Name: Febine

```
  1  GGATTAATTT TCCGCCGCCG CAGCCAGAAT ATCCCGACGG CTTTCCGCCT TCTGCTCCGG TTGGGTAAAG   70
 71  TAGGTCACCA GAACCAGCGG TGCGTGGTTT TCCGGCCAGA TAACCGCGAT ATCGTTGGTG GTGCCATAAT  140
141  CTCCGCTGCC GGTTTTATCG CCCACTACCC ATGATTTCGG CAGACCCGCC CGAATGCTCG CGCTACCGGT  210
211  AGTATTGCCC TTAAGCCACG TCACCAACTG TGCCCGCTGA GTTTCCGCCA GCGCTTTACC CAGCGTCAGA  280
281  TTTTTCAGGG TCTGCGCCAT CGCGAGCGGC GTGGTGGTAT CACGCGGGTC GCCTGGAATG GCGGTATTGA  350
351  GCGTGGGCTC GGTTCTGTCC AGACGGAAGG TCTCATCACC CAACGAGCGA GCAAACGCCG TCACTTTATC  420
421  GGGACCACCC AGATGGGCAA TCAGCTTATT CATGGCAGTA TTGTCGCTAT ACTGCAGCGC CGCTGCGCCA  490
491  AGCTCAGCCA GCGTCATCGT GCCGTTAACG TGTTTCTCCG CAATGGGATT GTAGTTAACC AGGTCGCTCT  560
561  TCTTGATTTC AACGCGCTGA TTTAGCAGGT GCTTATCGCT CTCGCTCTGT TTAAGCACCG CCGCGGCCGC  630
631  CATCACCTTA CTGGTACTGC ACATCGCAAA ACGTTCATCG GCACGGTAGA GAATCTGCGA ATTATCGGGC  700
701  GGGTGTTAAT CAGCGCAACG CCAAGCCGAC CTTCCC                                        736
```

Applied Biosystems

S/N G:84 A:37 T:36 C:41
KB.bcp
KB 1.4.0 Cap:2

S6_3130POP7_v3.1_2014-03-11
S6
KB_3130_POP7_BDTv3.mob
Pts 1840 to 11071 Pk1 Loc:1817
Version 5.3 HiSQV Bases: 696

Inst Model/Name 3100/3130GeneticAnalyzer-19348-006
Mar 11,2014 11:26PM, AST
Mar 11,2014 11:48PM, AST
Spacing:13.42 Pts/Panel1500
Plate Name: Febine

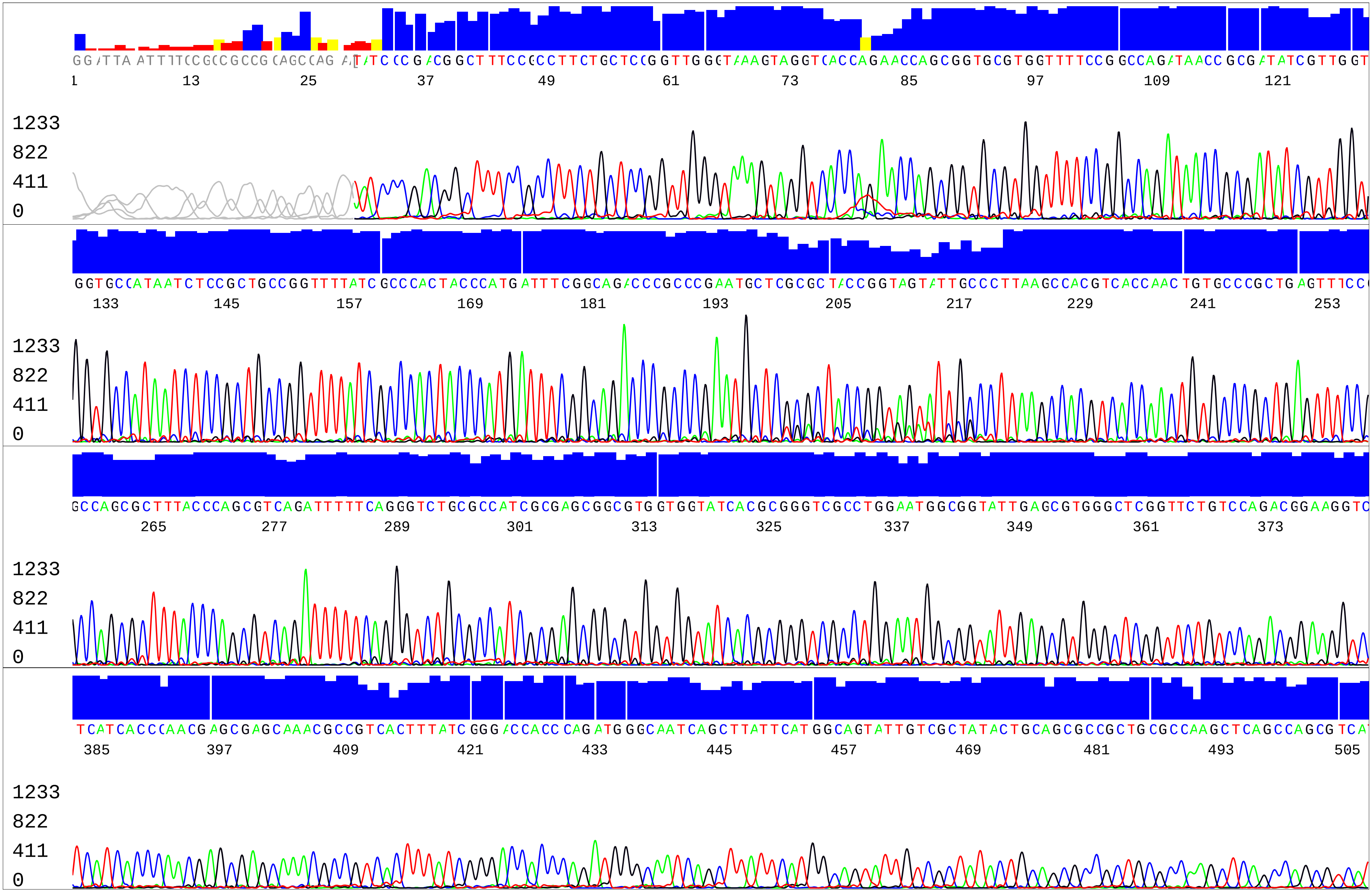

Applied Biosystems

S/N G:84 A:37 T:36 C:41
KB.bcp
KB 1.4.0 Cap:2

S6_3130POP7_v3.1_2014-03-11
S6
KB_3130_POP7_BDTv3.mob
Pts 1840 to 11071 Pk1 Loc:1817
Version 5.3 HiSQV Bases: 696

Inst Model/Name 3100/3130GeneticAnalyzer-19348-006
Mar 11,2014 11:26PM, AST
Mar 11,2014 11:48PM, AST
Spacing:13.42 Pts/Panel1500
Plate Name: Febine

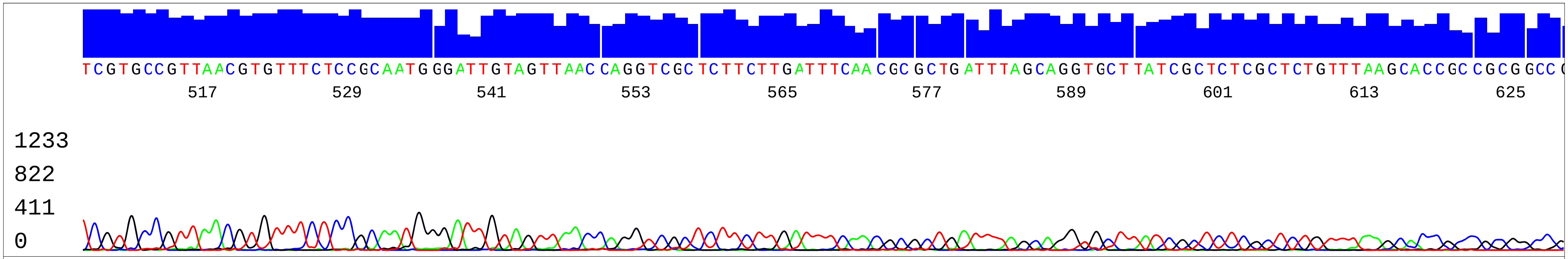

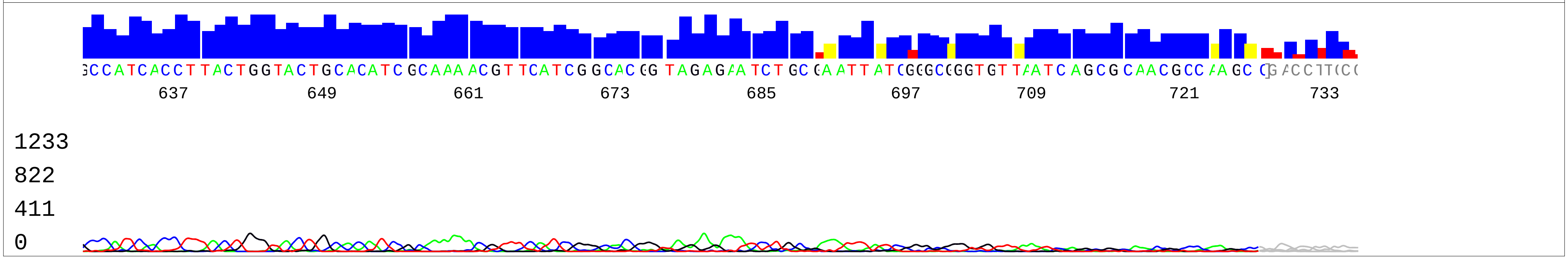

Supplement: Additional file 1: Table S1. — Specimen types and Demographics of E. coli O25b-B2-ST131 isolates. Samples from pus, skin and wound have been illustrated under soft tissue. [file 12866_2014_214_MOESM1_ESM.zip › 12866_2014_214_MOESM1_ESM/12866_2014_214_add9.pdf]
