# Supplementary figures and images for: The characterization and antibiotic resistance profiles of clinical Escherichia coli O25b-B2-ST131 isolates in Kuwait
Source: BMC Microbiol. 2014 Aug 28;14:214. doi: 10.1186/s12866-014-0214-6 (PMC4159528; doi:10.1186/s12866-014-0214-6)

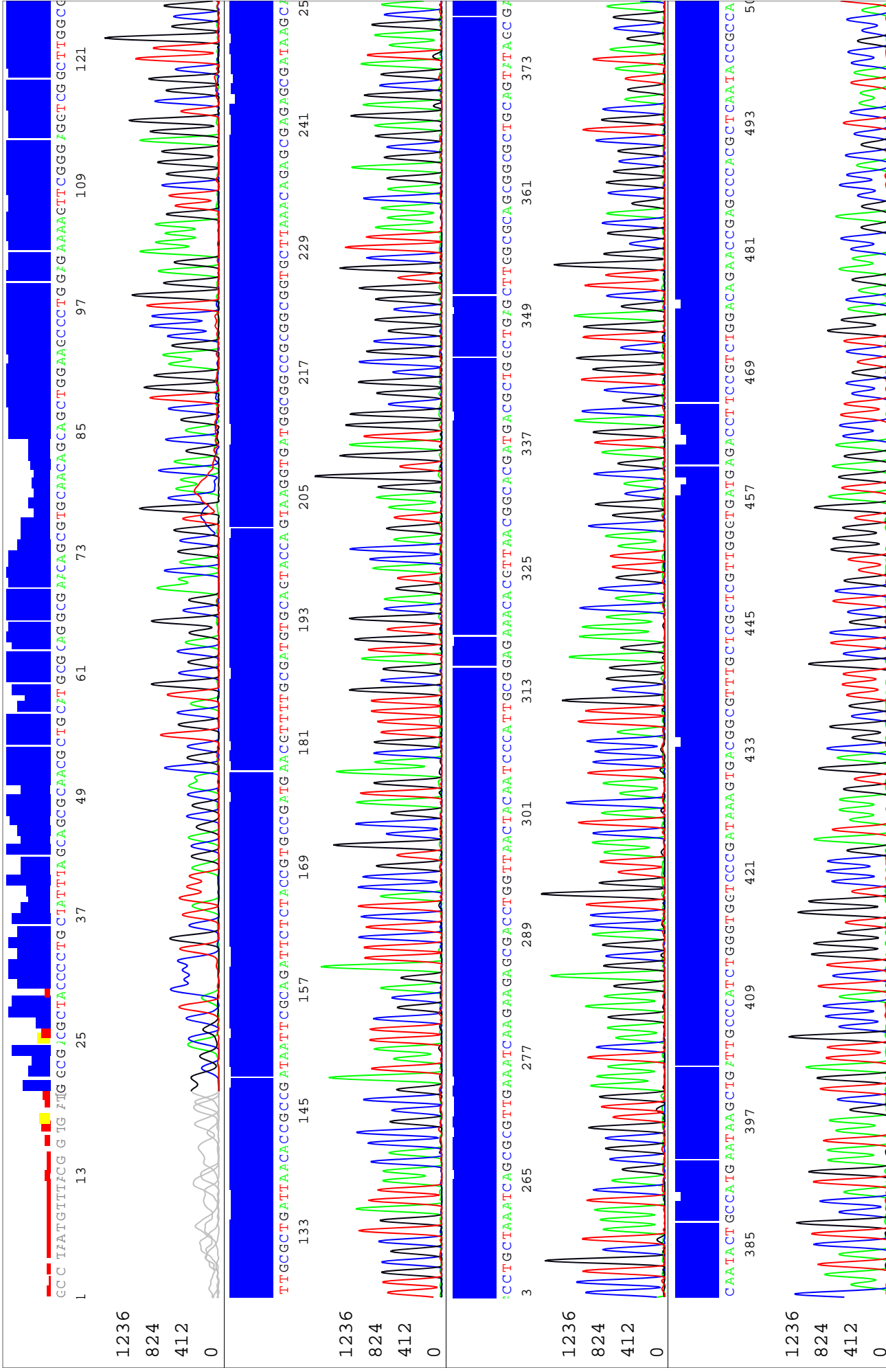

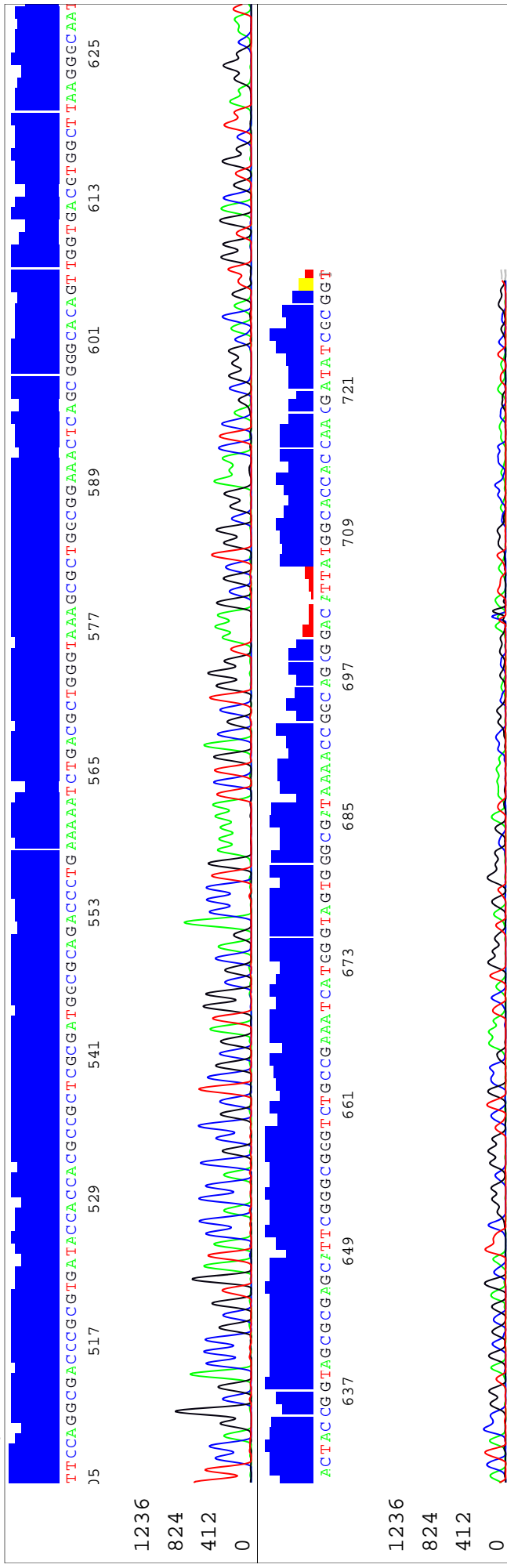

Supplement: Additional file 1: Table S1. — Specimen types and Demographics of E. coli O25b-B2-ST131 isolates. Samples from pus, skin and wound have been illustrated under soft tissue. [file 12866_2014_214_MOESM1_ESM.zip › 12866_2014_214_MOESM1_ESM/12866_2014_214_add12.pdf]

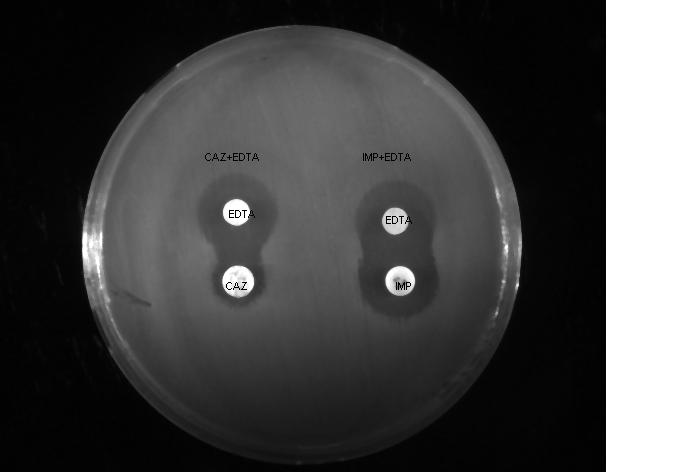

Supplement: Additional file 1: Table S1. — Specimen types and Demographics of E. coli O25b-B2-ST131 isolates. Samples from pus, skin and wound have been illustrated under soft tissue. [file 12866_2014_214_MOESM1_ESM.zip › 12866_2014_214_MOESM1_ESM/12866_2014_214_add36.jpeg]

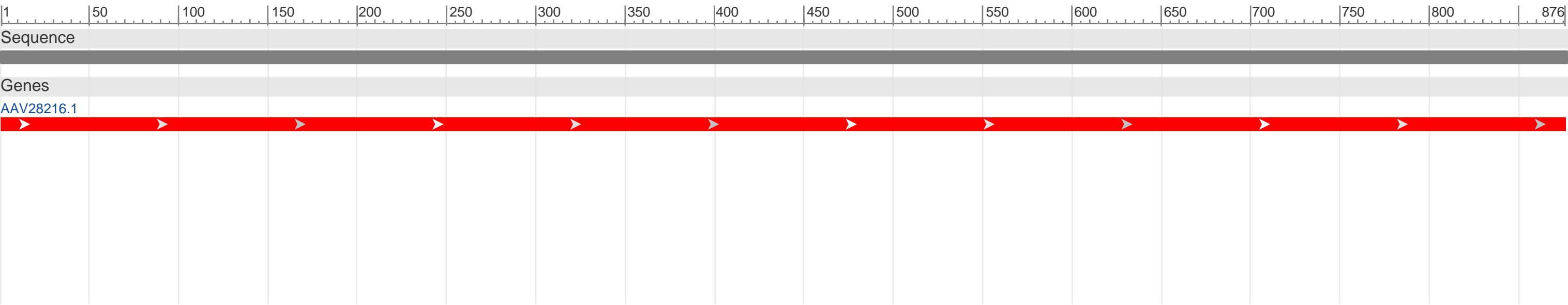

Supplement: Additional file 1: Table S1. — Specimen types and Demographics of E. coli O25b-B2-ST131 isolates. Samples from pus, skin and wound have been illustrated under soft tissue. [file 12866_2014_214_MOESM1_ESM.zip › 12866_2014_214_MOESM1_ESM/12866_2014_214_add4.pdf]
